# Gut microbiota-derived imidazole propionate predicts cardiometabolic risk in patients with coronary artery disease

**DOI:** 10.1093/eurheartj/ehaf661

**Published:** 2025-08-30

**Authors:** Florian A Wenzl, Peizhi Wang, Florian Kahles, Katharina R Beck, Evangelos Giannitsis, Slayman Obeid, Francesco Bruno, Xinmin S Li, David Nanchen, Luca Liberale, Maria A Smolle, Victor Schweiger, Roland Klingenberg, Robert Manka, Barbara E Stähli, Christian Templin, Maximilian König, Jinqing Yuan, Mustafa Yildirim, Michael Lehrke, Arnold von Eckardstein, Nikolaus Marx, François Mach, Lorenz Räber, Hugo A Katus, Elisabeth Steinhagen-Thiessen, Ilja Demuth, John Deanfield, Peter Libby, Stanley L Hazen, Arash Haghikia, Ulf Landmesser, Fredrik Bäckhed, Thomas F Lüscher

**Affiliations:** Center for Molecular Cardiology, University of Zürich, Wagistreet 12, 8952 Schlieren - Zurich, Switzerland; National Health Service England, Wellington House, 133-135 Waterloo Road, London SE1 8UG,United Kingdom; Department of Cardiovascular Sciences, University of Leicester, University Road LE1 7RH, Leicester, United Kingdom; Department of Clinical Sciences, Karolinska Institute, 171 77 Stockholm, Sweden; Center for Molecular Cardiology, University of Zürich, Wagistreet 12, 8952 Schlieren - Zurich, Switzerland; Department of Cardiology, State Key Laboratory of Complex Severe and Rare Diseases, Peking Union Medical College Hospital, Chinese Academy of Medical Sciences and Peking Union Medical College, Beijing, China; Department of Cardiology, National Clinical Research Center for Cardiovascular Diseases, Fuwai Hospital, National Center for Cardiovascular Diseases, Chinese Academy of Medical Sciences and Peking Union Medical College, Beijing, China; Department of Internal Medicine I, University Hospital Aachen, Aachen, Germany; Wallenberg Laboratory, Department of Molecular and Clinical Medicine and Sahlgrenska Center for Cardiovascular and Metabolic Research, University of Gothenburg, Gothenburg, Sweden; Department of Cardiology, University Hospital Heidelberg, Heidelberg, Germany; Division of Cardiology, Department of Medicine, Basel Cantonal Hospital, Basel, Switzerland; Division of Cardiology, Cardiovascular and Thoracic Department, Molinette Hospital, Città della Salute e della Scienza, Turin, Italy; Heart Division, Royal Brompton and Harefield Hospitals, Sydney Street, London SW3 6NP, United Kingdom; Departments of Cardiovascular and Metabolic Sciences, and Cardiovascular Medicine, Lerner Research Institute, Cleveland Clinic, Cleveland, OH, USA; Center for Microbiome and Human Health, Lerner Research Institute, Cleveland Clinic, Cleveland, OH, USA; Center for Primary Care and Public Health (Unisanté), University of Lausanne, Lausanne, Switzerland; First Clinic of Internal Medicine, Department of Internal Medicine, University of Genoa, Genoa, Italy; IRCCS Ospedale Policlinico San Martino Genoa, Italian Cardiovascular Network, Genoa, Italy; Comprehensive Cancer Center Graz, Medical University of Graz, Neue Stiftingtalstraße 6, Graz 8010, Austria; Department of Cardiology, University Hospital Zurich, Zurich, Switzerland; Department of Cardiology, Angiology and Intensive Care Medicine, Deutsches Herzzentrum der Charité, Berlin, Germany; Department of Cardiology, Krankenhaus Nordwest GmbH, Frankfurt, Germany; Department Medical Clinic I, Cardiology and Angiology, Justus-Liebig-University, Giessen, Germany; Department of Cardiology, University Heart Center, University Hospital Zurich, Zurich, Switzerland; Institute of Diagnostic and Interventional Radiology, University Hospital Zurich, University of Zurich, Zurich, Switzerland; Department of Cardiology, University Heart Center, University Hospital Zurich, University of Zurich, Zurich Switzerland; Center for Molecular Cardiology, University of Zürich, Wagistreet 12, 8952 Schlieren - Zurich, Switzerland; Department of Internal Medicine B, University Medicine Greifswald, Greifswald, Germany; Department of Internal Medicine and Geriatrics, University Medicine Greifswald, Greifswald, Germany; Department of Cardiology, National Clinical Research Center for Cardiovascular Diseases, Fuwai Hospital, National Center for Cardiovascular Diseases, Chinese Academy of Medical Sciences and Peking Union Medical College, Beijing, China; Department of Cardiology, University Hospital Heidelberg, Heidelberg, Germany; Department of Internal Medicine I, University Hospital Aachen, Aachen, Germany; Institute of Clinical Chemistry, University Hospital Zurich and University of Zurich, Zurich, Switzerland; Department of Internal Medicine I, University Hospital Aachen, Aachen, Germany; Department of Cardiology, Geneva University Hospital, Geneva, Switzerland; Department of Cardiology, Cardiovascular Center, University Hospital Bern, Bern, Switzerland; Department of Cardiology, University Hospital Heidelberg, Heidelberg, Germany; Friede Springe-Cardiovascular Prevention Center at Charité, Charité-Universitätsmedizin Berlin, Berlin, Germany; Department of Endocrinology and Metabolic Diseases (Including Division of Lipid Metabolism), Charité-Universitätsmedizin Berlin, Corporate Member of the Freie Universität Berlin and Humboldt-Universität zu Berlin, Berlin, Germany; Department of Endocrinology and Metabolic Diseases (Including Division of Lipid Metabolism), Charité-Universitätsmedizin Berlin, Corporate Member of the Freie Universität Berlin and Humboldt-Universität zu Berlin, Berlin, Germany; BCRT—Berlin Institute of Health Center for Regenerative Therapies, Charité—Universitätsmedizin Berlin, Berlin, Germany; Institute of Cardiovascular Science, University College London, London, United Kingdom; Division of Cardiovascular Medicine, Department of Medicine, Brigham and Women's Hospital, Harvard Medical School, Boston, MA, USA; Departments of Cardiovascular and Metabolic Sciences, and Cardiovascular Medicine, Lerner Research Institute, Cleveland Clinic, Cleveland, OH, USA; Center for Microbiome and Human Health, Lerner Research Institute, Cleveland Clinic, Cleveland, OH, USA; Department of Cardiovascular Medicine, Heart, Vascular and Thoracic Institute, Cleveland Clinic, Cleveland, OH, USA; Friede Springe-Cardiovascular Prevention Center at Charité, Charité-Universitätsmedizin Berlin, Berlin, Germany; Department of Cardiology, Angiology and Intensive Care Medicine, Deutsches Herzzentrum der Charité, Campus Benjamin Franklin, Berlin, Germany; German Center for Cardiovascular Research (DZHK), Partner Site Berlin, Berlin, Germany; Berlin Institute of Health at Charité-Universitätsmedizin Berlin, Berlin, Germany; University Hospital St. Josef-Hospital Bochum, Cardiology and Rhythmology, Ruhr University Bochum, Bochum, Germany; Friede Springe-Cardiovascular Prevention Center at Charité, Charité-Universitätsmedizin Berlin, Berlin, Germany; Department of Cardiology, Angiology and Intensive Care Medicine, Deutsches Herzzentrum der Charité, Campus Benjamin Franklin, Berlin, Germany; German Center for Cardiovascular Research (DZHK), Partner Site Berlin, Berlin, Germany; Berlin Institute of Health at Charité-Universitätsmedizin Berlin, Berlin, Germany; Wallenberg Laboratory, Department of Molecular and Clinical Medicine and Sahlgrenska Center for Cardiovascular and Metabolic Research, University of Gothenburg, Gothenburg, Sweden; Department of Clinical Physiology, Region Västra Götaland, Sahlgrenska University Hospital, Gothenburg, Sweden; Novo Nordisk Foundation Microbiome Health Initiative, Hellerup, Denmark; National Food Institute, Technical University of Denmark, Kongens Lyngby, Denmark; Center for Molecular Cardiology, University of Zürich, Wagistreet 12, 8952 Schlieren - Zurich, Switzerland; Heart Division, Royal Brompton and Harefield Hospitals, Sydney Street, London SW3 6NP, United Kingdom; National Heart and Lung Institute, Imperial College London, Dovehouse Street, London SW3 5NP, United Kingdom; School of Cardiovascular Medicine and Sciences, King's College London, Strand, London WC2R 2LS, United Kingdom

**Keywords:** Imidazole propionate, Gut microbiota, Cardiometabolic risk, Coronary artery disease

## Abstract

**Background and Aims:**

The gut microbiota is a modulator of cardiometabolic disease. Circulating imidazole propionate (ImP) is a microbiota-derived proatherogenic amino acid metabolite modulating the inflammatory response of myeloid cells, endothelial function, and glucose metabolism. This study examined the prognostic value of ImP in patients with coronary artery disease (CAD).

**Methods:**

Circulating ImP levels were measured in independent prospective cohorts of patients with acute coronary syndrome (ACS; Swiss ACS cohort *n* = 4787, Swiss cardiac magnetic resonance imaging cohort *n* = 150, German ACS cohort *n* = 1428) and chronic coronary syndrome (CCS; German CCS cohort *n* = 701). Major adverse cardiovascular events (MACE), defined as the first occurrence of a composite of death, non-fatal myocardial infarction, or non-fatal stroke after admission, were the primary endpoint. Cox models, accounting for established risk factors including the gut-derived cardiovascular risk factor trimethylamine N-oxide, were used to evaluate the predictive value of ImP.

**Results:**

Circulating ImP was associated with more advanced CAD and with cardiometabolic characteristics including diabetes and elevated high-sensitivity C-reactive protein. High ImP was an independent predictor of MACE [Swiss ACS cohort: hazard ratio (HR) per log2 increase 1.22, 95% confidence interval (CI) 1.10–1.35, *P* < .001; German ACS cohort: HR 2.34, 95% CI 1.46–3.76, *P* < .001; German CCS cohort: HR 1.32, 95% CI 1.13–1.53, *P* < .001)] and of mortality (Swiss ACS cohort: HR 1.34, 95% CI 1.17–1.54, *P* < .001; German ACS cohort: HR 2.38, 95% CI 1.48–3.82, *P* < .001; German CCS cohort: HR 1.50, 95% CI 1.14–1.98, *P* = .004) after adjustment for established risk factors. Imidazole propionate provided predictive value beyond trimethylamine N-oxide (Swiss ACS cohort: HR 1.30, 95% CI 1.05–1.61, *P* = .014; German CCS cohort: HR 1.31, 95% CI 1.12–1.53, *P* = .001).

**Conclusions:**

Gut microbiota-derived ImP predicted MACE in patients with CAD independently of traditional risk factors and holds promise as a therapeutic target. Imidazole propionate may refine risk stratification for personalized secondary prevention strategies.

## Structured Graphical Abstract

**Structured Graphical Abstract:**
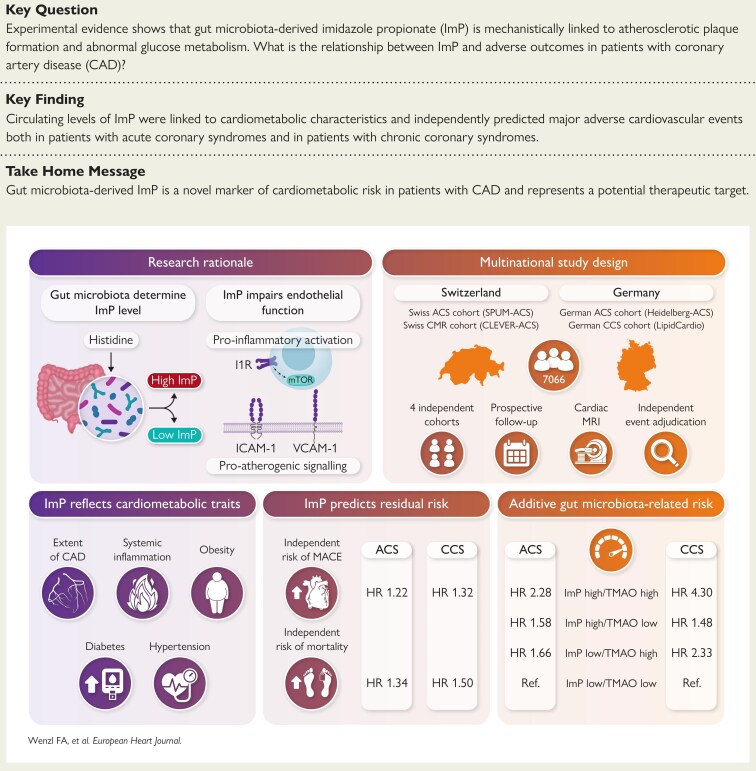
Gut microbiota-derived imidazole propionate (ImP) was identified as a novel marker of cardiometabolic risk in patients with coronary artery disease. Circulating ImP levels were measured in four independent cohorts of patients with acute (ACS) and chronic coronary syndrome (CCS) from Switzerland and Germany. Imidazole propionate results from microbial histidine metabolism and has been mechanistically linked to inflammatory activation of myeloid and endothelial cells driving atherogenesis. Circulating levels of ImP were associated with cardiometabolic traits and independently predicted major adverse cardiovascular events in patients with ACS and CCS. Imidazole propionate added to the gut microbiota-related risk beyond the established microbiota-derived metabolite trimethylamine N-oxide (TMAO). Joint groups of high (≥75th percentile; ImP ≥ 24.4 nanomolar [nM]) vs low (<75th percentile; ImP < 24.4 nM) circulating levels of ImP and TMAO were analysed. Values refer to hazard ratios per log2 increase in ImP level (bottom centre) and to hazard ratios using the ImP low/TMAO low group as reference (bottom right) in the fully adjusted model. Major adverse cardiovascular events were defined as a composite of death, non-fatal myocardial infarction, and non-fatal stroke after admission. ACS, acute coronary syndrome; CAD, coronary artery disease; CCS, chronic coronary syndrome; CMR, cardiac magnetic resonance imaging; HR, hazard ratio; ICAM-1, intercellular adhesion molecule 1; ImP, imidazole propionate; I1R, imidazoline-1 receptor; MACE, major adverse cardiovascular events; MRI, magnetic resonance imaging; mTOR, mechanistic target of rapamycin; TMAO, trimethylamine N-oxide; VCAM-1, vascular cell adhesion molecule 1.

**See the editorial comment for this article ‘Gut microbial metabolites and cardiometabolic disorders: new targets for prevention and treatment’, by L. Qi, https://doi.org/10.1093/eurheartj/ehaf844.**

## Introduction

Despite substantial advances in the control of traditional drivers of atherosclerosis, coronary artery disease (CAD) remains the leading cause of death in adults worldwide.^1,2^ With the evolution of risk factor profiles, attention has shifted increasingly to non-traditional cardiovascular risk factors.^3,4^

Imidazole propionate (ImP) is a gut microbiota-derived proatherogenic histidine metabolite^5–7^ that impairs endothelial function^8^ and glucose metabolism.^5,9^ At a molecular level, ImP induces inflammatory activation of myeloid and endothelial cells, hampers endothelial repair, and promotes atherogenesis. Circulating levels of ImP are primarily determined by the composition of the gut microbiota.^5,6^ Indeed, ImP is microbially produced by gut bacteria that are abundant in patients with type 2 diabetes^9,10^ and in individuals with subclinical coronary atherosclerosis.^11^

Imidazole propionate may thus constitute a novel therapeutic target for modulating cardiometabolic risk^12^ and holds potential to refine cardiovascular risk stratification. Clinical studies suggest that circulating ImP is associated with systemic inflammation,^10^ diabetes,^10^ heart failure,^13^ and subclinical atherosclerosis in asymptomatic volunteers.^7^ A recent study^8^ reported elevated ImP levels in subjects with CAD, yet extensive clinical investigations are lacking.

Here, we aim to assess the relationship between ImP and clinical outcomes in patients with CAD including acute coronary syndromes (ACSs) and chronic coronary syndromes (CCSs).

## Methods

### Study design and participants

We aimed to encompass the full spectrum of CAD including patients presenting with acute and stable clinical conditions. We used prospective ACS and CCS cohorts from Switzerland and Germany (see Supplementary data online, *Figures S1* and *S2*).

In the investigator-initiated prospective multicenter Swiss ACS cohort (SPUM-ACS Biomarker Study, ClinicalTrials.gov Identifier: NCT01000701), a total of 4787 patients with ACS were recruited from 8 December 2009 until 31 December 2017.^14–23^ Diagnoses of ACS were independently confirmed by expert cardiologists at local study facilities.^14,17^ Participants received guideline-directed therapy regimens, as reported previously.^17^ To investigate the coronary microcirculation, we analysed patients with ACS, enrolled in the Controlled Level EVERolimus in Acute Coronary Syndromes (CLEVER-ACS; ClinicalTrials.gov Identifier: NCT01529554) study from 26 November 2014 to 29 October 2021 undergoing cardiac magnetic resonance (CMR) imaging at 30-day follow-up (Swiss CMR cohort; *n* = 150).

The prospective German ACS cohort (Heidelberg II-ACS study) includes 1428 patients with ACS presenting to Heidelberg University Hospital, Heidelberg, Germany, between 14 September 2010 and 21 July 2014. Patients in this cohort were analysed using a case–cohort design, permitting unbiased assessment of exposure–outcome relationships, while reducing measurement costs. The design of case–cohort analyses has been reported previously,^24,25^ and a detailed description is provided in the Supplementary data online, *Supplementary material*. In brief, we selected a random sample of patients (i.e. the subcohort) from the pool of eligible participants. We then also selected all individuals with incident events who were not part of the subcohort. The final selection of patients (*n* = 250) consists of the subcohort and participants with incident events outside the subcohort.

The prospective German CCS cohort (LipidCardio Study, German Clinical Trial Register Identifier: DRKS00020915) included 701 consecutive patients with CCS undergoing elective diagnostic cardiac catheterization at the Department of Cardiology, Campus Benjamin Franklin, Charité-Universitätsmedizin, Berlin, Germany, between 27 October 2016 and 8 March 2018. Chronic coronary syndrome diagnoses were confirmed by angiographic evidence of obstructive CAD (≥50% stenosis).

The cohort profiles and detailed inclusion and exclusion criteria of the involved cohorts have been reported previously^26^ and are summarized in the Supplementary data online, *Supplementary material*.

### Imidazole propionate measurements

Blood samples were obtained at the time of presentation without a dedicated fasting interval and stored at −80°C. Imidazole propionate and its precursor molecules, histidine and *trans*-urocanate, were measured centrally using ultra-high-performance liquid chromatography coupled to tandem mass spectrometry. Samples were prepared and analysed at the University of Gothenburg, Gothenburg, Sweden, using an established method with minor modifications.^10^ In brief, 25 μL of sample volume was extracted with six volumes of acetonitrile containing internal standards (100 nanomolar [nM] ImP-^13^C_3_ and urocanate-^13^C_3_, AstraZeneca, Cambridge, United Kingdom, and 1 micromolar [µM] L-histidine D_5_, ^15^N_3,_ Cambridge Isotope Laboratories, Inc., Andover, MA, USA) before drying under a nitrogen stream. The samples were then reconstituted in 5% HCl (37%) in 1-butanol for *n*-butyl ester derivatization at 70°C for 40 min, dried, and finally reconstituted in 150 μL water/acetonitrile (9:1). Imidazole propionate, urocanate, and histidine levels were quantified using multiple reaction monitoring of the transitions 197.2/81.2, 195.2/93.0, and 213.2/110.1, respectively, and internal standards of the transitions 200.2/82.0, 198.2/95.0, and 220.3/118.1, respectively. Investigators performing the measurements were blinded to sample allocation and patient data other than the deidentified barcode label.

### Cardiac magnetic resonance imaging

In Switzerland, patients with ACS included in the CLEVER-ACS study underwent CMR imaging at 30-day follow-up according to a pre-specified study protocol. In brief, within 24 h after onset of chest pain, patients with proximal occlusion of a coronary artery underwent primary percutaneous coronary intervention (PCI) with placement of a drug-eluting stent in the culprit lesion.^27^ The CMR imaging protocol at 30-day follow-up included T1- and T2-weighted sequences for tissue characterization and calculation of ventricular volumes, function, and mass.^27^ Fifteen minutes after administration of a gadolinium chelate (0.20 mmol/kg of body weight), an inversion recovery fast-gradient echo imaging sequence was used to visualize scar tissue.^27^ The extent of coronary microvascular obstruction was quantified by contouring the dark core areas manually in the core of the necrotic zone.^27^ Imaging data analysis was performed in blinded fashion by expert physicians in the CMR Core Laboratory at the University Hospital Zurich, Zurich, Switzerland, employing dedicated heart imaging software (GTVolume, Gyrotools Ltd).

### Study endpoints, follow-up, and outcome adjudication

Major adverse cardiovascular events (MACE), defined as the first occurrence of a composite of death, non-fatal myocardial infarction, or non-fatal stroke after admission, were the primary endpoint. Mortality was analysed as an additional endpoint. A prediction horizon of 1 year (365 days) for the ACS cohorts and of 3 years (1095 days) for the German CCS cohort was used.

In Switzerland, patients with ACS were followed up at hospital discharge, at 30 days (via phone call) and at 1 year (clinical visit). Baseline and event data were collected by study personnel at the respective study centers via a centralized web-based data entry system (CARDIOBASE, Clinical Trial Unit and Department of Cardiology, University Hospital Bern, Bern, Switzerland, and Webspirit Systems GmbH, Ulm, Germany).^14^ Events were assessed based on pre-specified adjudication forms by an independent clinical endpoint committee composed of three certified experienced cardiologists, who were blinded to patients’ baseline characteristics.^14^ At each study site, a committee of expert cardiologists supervised data collection. In the German ACS cohort, patients were followed up via telephone call and/or a questionnaire forwarded by post or e-mail, as previously described.^28^ All deaths (retrieved via local resident registry records) and non-fatal adverse cardiovascular events were recorded.^28^ In the German CCS cohort, patients were followed up via phone call at 3 years after study enrolment^26^ to assess non-fatal adverse cardiovascular events.^26^ Mortality was retrieved from the obligatory registry office.^26^

### Statistical analyses

Continuous variables are presented as median and interquartile range.^14^ Categorical data are reported as counts and valid percentages.^14^ Continuous variables were compared with the Mann–Whitney *U* test for comparisons across two groups and the Kruskal–Wallis test for comparisons across three or more groups. Trends across independent groups were tested using the Jonckheere–Terpstra test. We used partial Spearman’s rank correlation to examine the association between pairs of continuous variables while adjusting for additional variables.^29^

The association of ImP with cardiometabolic characteristics was examined in the pooled CAD patient population (data from the Swiss ACS cohort and the German CCS cohort) to increase the number of patients in the analyses, as well as stratified by ACS and CCS status. Given the different clinical implications, risk modelling was stratified according to presentation with ACS and presentation with CCS. The association of circulating ImP with clinical outcomes was studied in complete case Cox proportional hazards regression models,^17,28,30^ using log2-transformed ImP levels,^14,31,32^ i.e. relative effect estimates correspond to a doubling in ImP levels. In addition, patients were divided according to ImP quartile (Q) and biomarker levels analysed on a categorical scale.^10,31^ Quartile cut-offs were derived from the largest cohort and applied to all cohorts (Q1: 9.4 nM; Q2: 9.4–14.3 nM; Q3: 14.3–24.4 nM; Q4: >24.4 nM; Supplementary data online). In a sensitivity analysis, cohort-specific ImP cut-offs were explored (Supplementary data online, *Table S19*).

Cumulative incidence curves were plotted, and differences between curves were examined using log-rank statistics. We constructed smoothed hazard ratio (HR) plots with three knots across the ImP distribution. Multivariable analyses were conducted using a stepwise approach adjusting for predefined covariables, based on sample size and clinical considerations (see Supplementary data online). Imidazole propionate levels were analysed in (i) crude, (ii) sex-, age-, and diabetes-adjusted, and (iii) fully adjusted models. In the fully adjusted model, covariables included age, sex, diabetes, high-sensitivity troponin T (hs-TnT), high-sensitivity C-reactive protein (hs-CRP), revascularization, ACS type (if applicable), body mass index, and estimated glomerular filtration rate. Analyses on mortality in patients with ACS were additionally adjusted for the GRACE 2.0 score for 1-year mortality.^33^ We further evaluated the predictive value of circulating ImP after additionally adjusting for its precursor molecules histidine and urocanate (see Supplementary data online, *Tables S15* and *S16*). Additionally, we accounted for ethnicity (only available in German CCS cohort), lipoprotein(a) [Lp(a)] levels, history of chronic liver disease, and leucocyte count in the Swiss ACS and in the German CCS cohorts (see Supplementary data online, *Tables S15* and *S16*). The proportional hazards assumption was checked in the fully adjusted model using Schoenfeld residuals (see Supplementary data online). In a subgroup of patients from the Swiss ACS cohort and the German CCS cohort with available trimethylamine N-oxide (TMAO) levels,^13,34^ the incremental predictive value of ImP beyond TMAO was assessed by additionally adjusting for TMAO. Moreover, we analysed joint groups of high (≥ Q4 cut-off) vs low (< Q4 cut-off) levels of ImP and TMAO yielding four strata: Low ImP/Low TMAO, Low ImP/High TMAO, High ImP/Low TMAO, and High ImP/High TMAO. Patients with CMR imaging at 30-day follow-up were stratified into two groups according to baseline ImP levels (i.e. Q4 vs Q1–3), and coronary microvascular obstruction was compared between groups using analysis of covariance adjusted for age, sex, diabetes, hypertension, low-density lipoprotein cholesterol, glucose, and pre-procedural coronary Thrombolysis in Myocardial Infarction flow grade. We conducted several subgroup analyses to assess the consistency of the results with respect to different population characteristics including sex, age, diabetes, proton pump inhibitor use, and dietary habits (only available in German CCS cohort). In addition, rehospitalization rates were examined in an exploratory analysis (see Supplementary data online, *Figure S14*). *P*-values and confidence intervals (CIs) are two-sided. The study was conducted in accordance with the principles of the STROBE statement.^35^ Statistical analyses were performed with R software version 4.3 or later and Stata version 16.1 or later. A detailed description of the statistical analyses is provided in the Supplementary data online.

## Results

### Circulating imidazole propionate is linked to the extent of coronary artery disease

**Figure 1:**
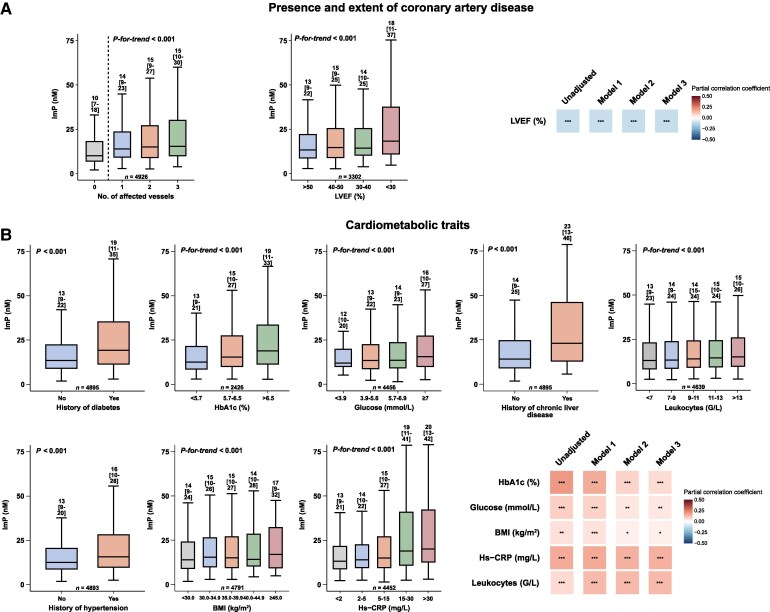
Circulating imidazole propionate is associated with extent of coronary artery disease and cardiometabolic traits. Circulating imidazole propionate levels according to extent of coronary artery disease (*A*) and cardiometabolic traits (*B*). Data are represented as boxplots and correlation plots. Medians with interquartile ranges are displayed on top of each box. Spearman’s partial correlation coefficients and *P*-values were calculated using partial correlation adjusted for Model 1: age and sex, Model 2: Model 1 plus diabetes status, and Model 3: Model 2 plus hypercholesterolaemia. False discovery rate-adjusted using Benjamini–Hochberg correction. *P < .05; **P < .01. Values in the group with 0 affected vessels refer to the reference population without coronary artery disease. The results are based on pooled data from the Swiss Acute Coronary Syndrome cohort and the German Chronic Coronary Syndrome cohort. See also Supplementary data online, *Table S13*.

A total of 7066 patients were enrolled in Switzerland and Germany (Swiss ACS cohort *n* = 4787, Swiss CMR cohort *n* = 150, German ACS cohort *n* = 1428, German CCS cohort *n* = 701; see Supplementary data online, *Figures S1*). Baseline and treatment characteristics are summarized in *Tables 1* and *2* and Supplementary data online, *Tables S1*–*S12,*  *S20*. Circulating ImP levels were significantly elevated in patients with CAD (*Figure 1A*). Moreover, high ImP was associated with more advanced atherosclerotic disease, as determined by a higher number of affected coronary arteries, lower left ventricular ejection fraction, and more severe symptoms of heart failure (*Figure 1A*; Supplementary data online, *Figure S9*). These findings were consistent across clinical presentation with ACS and CCS (see Supplementary data online, *Figures S7* and *S9*) but absent or less pronounced for the precursor molecules histidine and urocanate (see Supplementary data online, *Figures S6*, *S8*, and *S10* and *Table S13*). Imidazole propionate levels were comparable across different times of the day (see Supplementary data online, *Figures S3* and *S4*).

**Table 1:** Baseline characteristics of patients with acute or chronic coronary syndromes

	Swiss ACS cohort (*n* = 4318)	German ACS cohort (*n* = 1428)	German CCS cohort (*n* = 577)
Age (years)	63 (54–73)	68 (57–76)	76 (67–81)
Female	881/4318 (20.4%)	440/1428 (30.8%)	124/577 (21.5%)
BMI (kg/m²)	26.6 (24.2–29.4)	27.1 (25.0–30.5)^a^	26.9 (24.5–30.0)
Body surface area (m²)	1.9 (1.8–2.1)		2.0 (1.8–2.1)
Heart rate (b.p.m.)	75 (66–86)	74 (65–85)	
Systolic blood pressure (mm Hg)	128 (112–143)	150 (137–166)	134 (121–149)
Signs and symptoms of heart failure (ACS: Killip class, CCS: NYHA class)			
I	3682/4204 (87.6%)	1365/1428 (95.6%)	93/555 (16.8%)
II	343/4204 (8.2%)	50/1428 (3.5%)	294/555 (53.0%)
III	85/4204 (2.0%)	11/1428 (0.8%)	166/555 (29.9%)
IV	94/4204 (2.2%)	2/1428 (0.1%)	2/555 (0.4%)
Smoking	1649/4254 (38.8%)	449/1371 (32.8%)	98/577 (17.0%)
Medical history			
Diabetes	753/4318 (17.4%)	319/1414 (22.6%)	178/577 (30.8%)
Pre-diabetes^b#^	630/2153 (29.3%)	194/670 (29.0%)	168/577 (29.1%)
Hypertension	2413/4316 (55.9%)	1099/1417 (77.6%)	477/577 (82.7%)
Hyperlipoproteinaemia	2757/4317 (63.9%)	838/1369 (61.2%)	378/577 (65.5%)
Myocardial infarction	524/4310 (12.2%)	344/1402 (24.5%)	405/576 (70.3%)
Family history of CAD	1056/4259 (24.8%)	491/1352 (36.3%)	161/478 (33.7%)
Prior PCI	644/4317 (14.9%)	498/1400 (35.6%)	
Prior CABG	169/4318 (3.9%)	157/1399 (11.2%)	62/577 (10.7%)
Cerebrovascular disease	160/4318 (3.7%)		74/577 (12.8%)
Peripheral artery disease	251/4318 (5.8%)		30/577 (5.2%)
Heart failure	54/4316 (1.3%)	294/1348 (21.8%)	
Chronic liver disease	25/4318 (0.6%)	3/139 (2.2%)^a^	12/577 (2.1%)
Dialysis	21/4318 (0.5%)		
Clinical chemistry and haematology			
Leucocyte count (G/L)	9.6 (7.4–12.2)	7.6 (5.6–13.3)^a^	7.4 (6.0–8.9)
hs-CRP (mg/L)	3 (1–7)	4 (2–12)	2 (1–7)
Total cholesterol (mg/dL)	190 (159–220)	189 (172–206)^a^	153 (129–187)
LDL-C (mg/dL)	120 (92–149)	91 (79–106)^a^	85 (65–115)
HDL-C (mg/dL)	44 (36–53)	47 (43–50)^a^	46 (38–57)
Lipoprotein (a) (mg/dL)	6.4 (2.5–21.8)		8.3 (2.0–34.9)
Triglycerides (mg/dL)	88 (60–136)	148 (109–185)^a^	121 (91–176)
HbA1c (%)	5.8 (5.5–6.3)	5.8 (5.5–6.3)	5.8 (5.5–6.3)
Glucose (mmol/L)	6.4 (5.6–7.9)	6.6 (5.7–8.2)	5.9 (5.2–7.7)
NT-proBNP (ng/L)	334 (108–1158)	586 (164–2440)	427 (134–1238)
hs-TnT (ng/L)	196 (59–642)	46 (9–220)	15 (9–30)
Creatinine (mg/dL)	0.9 (0.7–1.0)	0.9 (0.8–1.1)^a^	1.0 (0.8–1.2)
eGFR (mL/min/1.73 m^2^)	93 (77–103)	86 (76–96)	73 (59–90)
ImP metabolism			
ImP (nM)	14 (9–24)	13 (9–20)^a^	13 (8–26)
Urocanate (nM)	44 (30–66)	128 (81–203)^a^	47 (33–84)
Histidine (µM)	66 (57–76)	88 (68–103)^a^	62 (53–70)
Baseline medication			
Aspirin	1230/2779 (44.3%)	33/139 (23.7%)^a^	381/577 (66.0%)
P2Y12 inhibitor	321/1915 (16.8%)		192/577 (33.3%)
Beta-blocker	978/2779 (35.2%)	22/139 (15.8%)^a^	371/577 (64.3%)
ACE inhibitor/ARB	1475/2759 (53.5%)	100/139 (71.9%)^a^	447/577 (77.5%)
Vitamin K antagonist/DOAC	171/2777 (6.2%)	19/139 (13.7%)^a^	148/577 (25.6%)
Diuretic	674/2773 (24.3%)	50/139 (36.0%)^a^	247/577 (42.8%)
Insulin	214/2777 (7.7%)	35/139 (25.2%)^a^	57/577 (9.9%)
Oral antidiabetics	503/2777 (18.1%)	4/139 (2.9%)^a^	108/577 (18.7%)
Immunosuppressive drugs	119/2777 (4.3%)		
PPI	643/2777 (23.2%)	32/139 (23.0%)^a^	247/577 (42.8%)

Categorical data are shown as numbers and valid percentages (%). Continuous data are presented as median and interquartile range (IQR).

ACE, angiotensin-converting enzyme; ACS, acute coronary syndrome; ARB, angiotensin II receptor blocker; BMI, body mass index; CABG, coronary artery bypass grafting; CAD, coronary artery disease; CCS, chronic coronary syndrome; DOAC, direct oral anticoagulant; eGFR, estimated glomerular filtration rate; HbA1c, haemoglobin A1c; HDL-C, high-density lipoprotein cholesterol; hs-CRP, high-sensitivity C-reactive protein; hs-TnT, high-sensitivity troponin T; ImP, imidazole propionate; LDL-C, low-density lipoprotein cholesterol; NT-proBNP, N-terminal pro B-type natriuretic peptide; NYHA, New York Heart Association; PCI, percutaneous coronary intervention; PPI, proton pump inhibitor.

^a^Values refer to the subcohort.

^b#^HbA1c of 5.7%–6.4% in individuals without a history of diabetes.

**Table 2:** Treatment characteristics of patients with acute or chronic coronary syndromes

	Swiss ACS cohort (*n* = 4318)	German ACS cohort (*n* = 1428)	German CCS cohort (*n* = 577)
Revascularization strategy during baseline visit			
PCI	3949/4318 (91.5%)	843/1283 (65.7%)	350/577 (60.7%)
CABG	127/4318 (2.9%)		
Symptom-to-door time (minutes)	180 (105–420)	480 (120–2160)	
No. of affected vessels^a^			
One	3371/4095 (82.3%)	209/1283 (16.3%)	135/577 (23.4%)
Two	563/4095 (13.7%)	237/1283 (18.5%)	194/577 (33.6%)
Three	161/4095 (3.9%)	724/1283 (56.4%)	248/577 (43.0%)
Left ventricular ejection fraction (%)			
>50	1380/2725 (50.6%)	420/1361 (30.9%)	437/577 (75.7%)
50–40	858/2725 (31.5%)	418/1361 (30.7%)	81/577 (14.0%)
40–30	314/2725 (11.5%)	349/1361 (25.6%)	38/577 (6.6%)
<30	173/2725 (6.3%)	174/1361 (12.8%)	21/577 (3.6%)
Discharge medication			
Aspirin	4233/4273 (99.1%)	1259/1384 (91.0%)	
P2Y12 inhibitor	4070/4163 (97.8%)	989/1393 (71.0%)	
Beta-blocker	3339/4269 (78.2%)	1227/1393 (88.1%)	
ACE inhibitor/ARB	3749/4271 (87.8%)	1218/1393 (87.4%)	
Vitamin K antagonist/DOAC	303/4318 (7.0%)	147/1391 (10.6%)	
Diuretic	997/4272 (23.3%)		
Insulin	277/4270 (6.5%)	108/1392 (7.8%)	
Oral antidiabetics	503/4270 (11.8%)	175/1392 (12.6%)	
Immunosuppressive drugs	112/4272 (2.6%)		
PPI	1258/4272 (29.4%)	40/139 (15.8%)^c^	
Outcomes^b^			
MACE	314/4318 (7.3%)	117/1428 (8.2%)	164/577 (28.42%)
Mortality	148/4318 (3.4%)	75/1428 (5.3%)	47/577 (8.15%)

Categorical data are shown as numbers and valid percentages (%). Continuous data are presented as median and interquartile range (IQR).

ACE, angiotensin-converting enzyme; ACS, acute coronary syndrome; ARB, angiotensin II receptor blocker; CABG, coronary artery bypass grafting; CCS, chronic coronary syndrome; DOAC, direct oral anticoagulant; MACE, major adverse cardiovascular events; PCI, percutaneous coronary intervention; PPI, proton pump inhibitor.

^a^Vessels with significant coronary stenosis in Heidelberg II-ACS and LipidCardio. In Heidelberg II-ACS, no significant coronary stenosis was defined in 113 patients.

^b^1-year outcomes for ACS and to 3-year outcomes for CCS included in the analyses.

^c^Values refer to the subcohort.

### High imidazole propionate associates with cardiometabolic characteristics

Given the established effects of ImP on glucose metabolism, we studied its relationship to cardiometabolic characteristics of patients with CAD. High ImP was associated with high body mass index, systemic inflammation, impaired glucose metabolism, and hypertension (*Figure 1B*; Supplementary data online, *Figure S11*). These results were similarly observed in patients with ACS and in patients with CCS (see Supplementary data online, *Figures S7* and *S9*) but were absent or less pronounced for the precursor molecules histidine and urocanate (see Supplementary data online, *Figures S8* and *S10*).

### High imidazole propionate is associated with coronary microvascular obstruction

**Figure 2:**
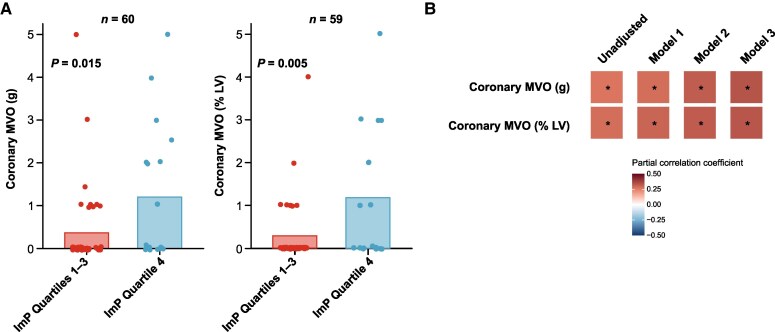
Imidazole propionate associates with coronary microvascular obstruction. (*A*) Coronary microvascular obstruction in patients undergoing primary percutaneous coronary intervention according to imidazole propionate (Q1: 0–9.4 nM; Q2: 9.4–14.3 nM; Q3: 14.3–24.4 nM; Q4: >24.4 nM). Bars are mean values. (*B*) Correlation of ImP and coronary microvascular obstruction. Spearman’s partial correlation coefficients and *P*-values were calculated using partial correlations adjusted for Model 1: age, sex, and diabetes status, Model 2: Model 1 plus hypertension, low-density lipoprotein cholesterol, and glucose, and Model 3: Model 2 plus pre-procedural coronary flow based on thrombolysis in myocardial infarction classification. False discovery rate-adjusted using Benjamini–Hochberg correction. *P < .05; **P < .01. The results in all figure panels are based on data from the Swiss Cardiac Magnetic Resonance cohort. See also Supplementary data online, *Table S14* and *Figure S12*

Considering the intricate involvement of ImP in central processes of myocardial microcirculation in preclinical experiments including endothelial dysfunction, regulation of endothelium-dependent vascular tone, leucocyte function and adhesion, and osmotic regulation, we performed an exploratory analysis to examine a potential association of ImP with CMR-assessed microvascular obstruction. High ImP levels were associated with increased coronary microvascular obstruction at 30 days (*Figure 2*; Supplementary data online, *Table S14*). These results remained consistent on multivariable adjustment but were not observed for the precursor molecules histidine and urocanate (see Supplementary data online, *Figure S12*).

### High imidazole propionate predicts cardiovascular outcomes beyond established risk factors and GRACE 2.0

**Figure 3:**
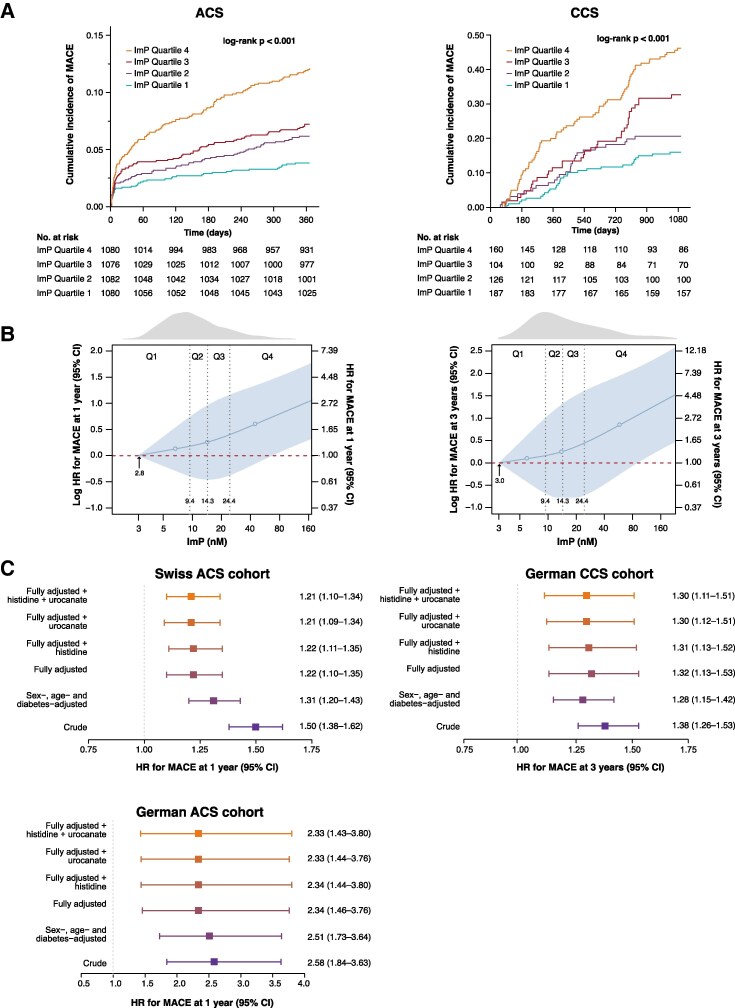
Imidazole propionate predicts cardiovascular outcomes in patients with acute and chronic coronary syndromes. (*A*) Cumulative event curves for major adverse cardiovascular events, defined as a composite of death, non-fatal myocardial infarction, and non-fatal stroke after admission, of patients presenting with acute (left) and chronic (right) coronary syndrome stratified by circulating imidazole propionate level (Q1: 0–9.4 nM; Q2: 9.4–14.3 nM; Q3: 14.3–24.4 nM; Q4: >24.4 nM). (*B*) Restricted cubic spline plot showing the adjusted hazard ratio for major adverse cardiovascular events at 1 and 3 years according to imidazole propionate level in patients with acute (left; *n* = 3966) and chronic (right; *n* = 385) coronary syndromes, respectively. The solid line indicates the predicted hazard ratio with the color band signifying the corresponding 95% confidence interval. Three knots were fixed at the 10th, 50th, and 90th percentile of the imidazole propionate distribution. Note that hazard ratios are shown in log scale (left *Y* axis) and as untransformed values (right *Y* axis). The dashed horizontal line indicates the reference. Dashed vertical lines mark quartile cut-offs (Q1: 0–9.4 nM; Q2: 9.4–14.3 nM; Q3: 14.3–24.4 nM; Q4: >24.4 nM). (*C*) Crude and adjusted hazard ratios for major adverse cardiovascular events at 1 and 3 years in patients with acute (left) and chronic (right) coronary syndromes, respectively. Cox proportional hazards regression models accounted for established risk factors and precursor molecules of imidazole propionate in a stepwise fashion. Squares indicate point estimates of the hazard ratio per log2 increase and line lengths equal corresponding 95% confidence intervals. Plots in (*A*) and (*B*) are based on data from the Swiss Acute Coronary Syndrome cohort and the German Chronic Coronary Syndrome cohort, respectively. Patient numbers and event counts for (*C*) are summarized in the Supplementary data online, *Table S15*.

During the observation period, there were a total of 595 MACE and 270 deaths (*Table 2*). Cumulative incidence curves showed a substantial increase in MACE and mortality in patients with high ImP with HR plots indicating a dose–response relationship (*Figure 3*). After multivariable adjustment, high ImP levels were associated with increased risk of MACE (Swiss ACS cohort: fully adjusted HR per log2 increase 1.22, 95% CI 1.10–1.35, *P* < .001, fully adjusted HR Q4 vs Q1–3, 1.33, 95% CI 1.03–1.72, P = .032; German ACS cohort: fully adjusted HR per log2 increase, 2.34, 95% CI 1.46–3.76, P < .001, fully adjusted HR Q4 vs Q1–3, 7.97, 95% CI 2.70–23.52, P < .001; German CCS cohort: fully adjusted HR per log2 increase, 1.32, 95% CI 1.13–1.53, P < .001, fully adjusted HR Q4 vs Q1–3 1.83, 95% CI 1.22–2.74, *P* = .004; Supplementary data online, *Table S15*). Likewise, high plasma ImP emerged as a strong independent predictor of all-cause mortality (Swiss ACS cohort: fully adjusted + GRACE 2.0 HR per log2 increase 1.24, 95% CI 1.06–1.46, *P* = .007, fully adjusted HR + GRACE 2.0 Q4 vs Q1–3 1.47, 95% CI 0.96–2.26, *P* = .076; German ACS cohort: fully adjusted + GRACE 2.0 HR per log2 increase 2.32, 95% CI 1.46–3.41, *P* < .001, fully adjusted HR + GRACE 2.0 Q4 vs Q1–3 4.84, 95% CI 1.42–16.47, *P* = .012; German CCS cohort: fully adjusted HR per log2 increase 1.50, 95% CI 1.14–1.98, *P* = .004, fully adjusted HR Q4 vs Q1–3 2.01, 95% CI 0.96–4.19, *P* = .063; Supplementary data online, *Table S16*). These findings were consistent across patient subgroups (see Supplementary data online, *Tables S17* and *S18* and *Figure S5*). Similar results were obtained when additionally adjusting for Lp(a), liver disease, leucocyte count, and the precursor molecules of ImP (see Supplementary data online, *Tables S15* and *S16*).

### Imidazole propionate adds to microbiota-related risk beyond trimethylamine N-oxide

**Figure 4:**
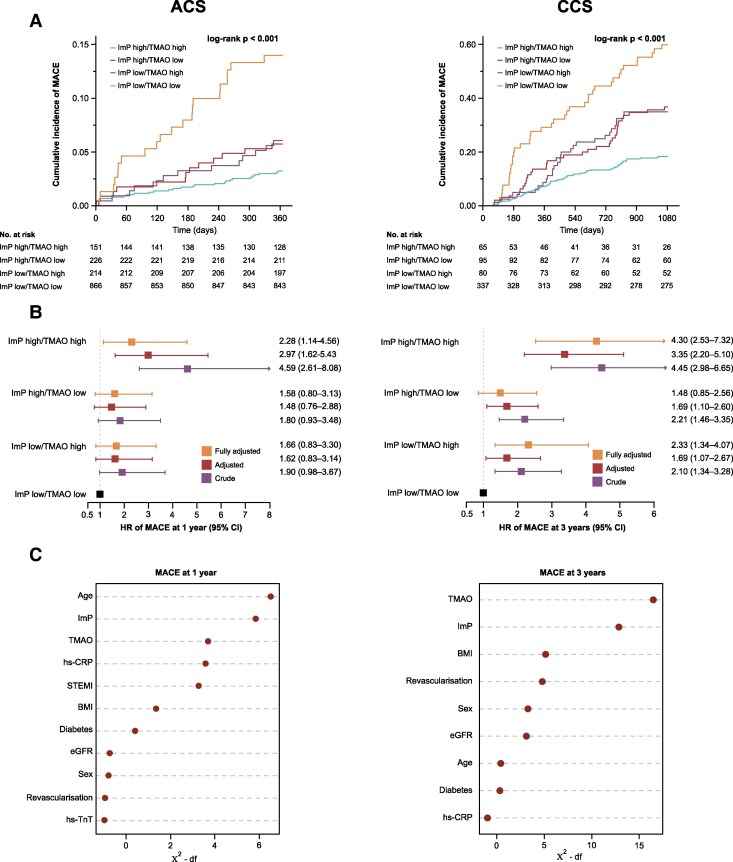
Imidazole propionate predicts adverse cardiovascular outcomes beyond trimethylamine N-oxide. (*A*) Cumulative event curves for major adverse cardiovascular events, defined as a composite of death, non-fatal myocardial infarction, and non-fatal stroke after admission in patients presenting with acute coronary syndrome and chronic coronary syndrome according to joint groups of high (≥75th percentile; Q4: >24.4 nM) vs low (<75th percentile; Q1–Q3: 0–24.4 nM) levels of imidazole propionate and trimethylamine N-oxide (TMAO). (*B*) Multivariable-adjusted hazard ratio and 95% confidence interval for major adverse cardiovascular events at 1 year in patients with acute coronary syndrome and at 3 years in patients with chronic coronary syndrome, according to imidazole propionate quartile (Q1: 0–9.4 nM; Q2: 9.4–14.3 nM; Q3: 14.3–24.4 nM; Q4: >24.4 nM). Squares indicate point estimates and line lengths equal corresponding 95% confidence intervals. (*C*) Feature importance for the prediction of major adverse cardiovascular events measured by partial Wald χ² minus the degrees of freedom. The results are based on 1281 patients from the Swiss Acute Coronary Syndrome cohort and 385 patients from the German Chronic Coronary Syndrome cohort in the fully adjusted model.

Imidazole propionate provided incremental predictive value for MACE beyond TMAO (Swiss ACS cohort: fully adjusted + TMAO HR per log2 increase 1.30, 95% CI 1.05–1.61, *P* = .014; German CCS cohort: fully adjusted + TMAO HR per log2 increase 1.31, 95% CI 1.12–1.53, *P* = .001; Supplementary data online, *Table S21*). When stratifying patients into joint groups according to both ImP and TMAO level, individuals with high levels of both markers exhibited excessive risk of MACE (*Figure 4A*). After multivariable adjustment, joint elevation of ImP and TMAO conferred a more than two-fold increased risk of MACE in patients with ACS and CCS (Swiss ACS cohort: fully adjusted HR 2.28, 95% CI 1.14–4.56, *P* = .020; German CCS cohort: fully adjusted HR 4.30, 95% CI 2.53–7.32, *P* < .001; *Figure 4B*). Circulating ImP ranked among the strongest predictors of MACE (*Figure 4C*). These findings were similarly observed for mortality (see Supplementary data online, *Figure S13* and *Table S22*).

## Discussion

Here, we show for the first time that the gut microbiota-derived amino acid metabolite ImP is linked to cardiometabolic characteristics and independently predicts adverse outcomes in patients with ACS and CCS beyond established risk markers (*Structured Graphical Abstract*).

Despite advancements in the management of patients with CAD over the past decades, there is considerable residual cardiovascular risk attributed to non-traditional risk factors such as inflammation, disturbed sleep, air pollution, physical inactivity, environmental stress, and microbial dysbiosis. Imidazole propionate is produced from urocanate, a metabolite of the essential amino acid histidine, by the microbial enzyme urocanate reductase.^6^ Previous studies have shown that circulating ImP levels are primarily determined by the composition and metabolism of gut microbiota, rather than histidine intake.^10^

At the cellular level, ImP induces imidazoline-1 receptor signalling^7^ and inhibits insulin-receptor signalling,^5,9^ in turn leading to activation of inflammation-associated pathways in macrophages, fibroblasts and endothelial cells,^8^ impaired endothelial cell regeneration after vascular injury, reduced endothelium-dependent arterial relaxation, impaired glucose metabolism,^5,6^ and increased atherogenesis.^8^ Notably, ImP promotes the expression of leucocyte adhesion molecules for leucocyte–endothelial cell interactions, leading to an inflammatory milieu at the endothelial interface and increased immune cell infiltration, a hallmark of atherosclerosis.^34^ The atherogenic effects of ImP involve different molecular pathways compared with TMAO.^36–38^

In line with previous mechanistic studies, our results demonstrate that ImP is associated with signs of cardiometabolic dysregulation and more extensive coronary atherosclerosis in patients with CAD. An exploratory analysis of a small cohort of patients with ACS undergoing primary PCI followed up with CMR indicated that patients with high ImP also had more extensive coronary microvascular obstruction. These findings align well with preclinical studies showing that ImP can influence key processes related to the microcirculation including endothelial cell regeneration, regulation of the endothelium-dependent vascular tone,^8^ leucocyte adhesion, and osmotic regulation. This suggests the possibility that ImP may contribute to the individual susceptibility to microvascular dysfunction in response to ischaemia–reperfusion injury,^39^ as encountered in patients with ACS undergoing primary PCI. In contrast, no such associations were observed for the precursor molecules histidine and urocanate.

Our study affirms that inter-individual heterogeneity in the gut microbiome has important implications for clinical risk stratification. Circulating ImP was strongly associated with both MACE and all-cause mortality. These findings were consistent across patients with ACS and CCS, geographies, subgroups, and follow-up periods. Notably, high ImP conferred incremental gut microbiota-related risk above and beyond TMAO, an established gut-derived cardiovascular risk factor. Patients with high levels of both metabolites had more than double the risk of MACE after accounting for established risk factors. Indeed, elevation of both ImP and TMAO may characterize a novel gut microbiota-related signature associated with increased cardiovascular risk. Compared with established risk factors, ImP ranked among the strongest predictors of MACE holding promise to refine risk stratification for personalized secondary prevention strategies. Moreover, the absence of these findings for precursor molecules of ImP supports a potential causal contribution of ImP to adverse outcomes. Currently, no cut-off value for elevated ImP is established in the general population or in patients with CAD. In this study, we observed particularly high risk of adverse events in patients in the highest ImP quartile (i.e. >24 nM).

The biological actions of ImP and its complex interplay with cardiometabolic disease are the subject of ongoing investigations. While more studies are needed to decipher its precise role in atherogenesis and identify thresholds for optimal risk stratification, future treatment approaches^8^ may target microbial ImP production by inhibiting the microbial enzyme urocanate reductase to modulate cardiometabolic risk.^12^ The management of traditional cardiovascular risk factors has significantly improved outcomes in the past decades, yet the current drug armamentarium lacks strategies directed at modulating the microbiome and its metabolites. The potential integration of the gut–heart axis^40^ in future treatment approaches holds promise for more personalized and effective secondary prevention.

In conclusion, our results identify circulating ImP as a novel biomarker of cardiometabolic risk in patients with CAD and a potential therapeutic target for secondary prevention.

### Strengths and limitations

This study has several strengths. First, the rigorous multinational design including external validation of the results in independent, well-characterized cohorts from different countries confirmed the external validity of the findings and accounts for regional differences in treatment practices, patient characteristics, and gut microbiota composition. Second, the SPUM-ACS Biomarker Study comprises one of the largest and best characterized cohorts of patients with ACS worldwide. Its prospective multicenter design together with a contemporary patient population undergoing guideline-based treatment approaches grants for high quality of the collected data in SPUM-ACS. Moreover, the central assessment of established biomarkers including hs-TnT and hs-CRP in the core laboratory at the University Hospital Zurich, Zurich, Switzerland, using a validated assay minimizes the impact of analytical variability. Third, assessment bias is prevented by external adjudication of clinical outcomes during follow-up by an independent clinical event adjudication committee. Further, we centralized blinded measurement of ImP according to established protocols enhancing the technical validity of the measurements and the comparability of the results. Finally, we also measured the precursor molecules of ImP to inform hypothesis generation regarding potential causal relationships.

Our study also has limitations. First, there were a limited number of events at 1 year in the validation cohort. However, the results were consistent across endpoints and at 3 years considering a higher number of events. Second, for external validation of our results in patients with ACS, we did not measure ImP in all patients of the German ACS cohort due to the use of a case–cohort design. Nonetheless, the results of the case–cohort analysis are statistically robust and provide valid approximations of full-cohort analyses. Third, a limited number of female patients (less than a third across all cohorts) were included, and ethnicity was only available in the German CCS cohort. Of note, we considered these variables in specific subgroup analyses and adjusted models. Nevertheless, further studies are required to validate our findings in more ethnically diverse cohorts, accounting for differences in dietary patterns across geographies and ethnicities. In addition, due to limited availability of TMAO, analyses including this variable could only be performed in a subcohort of patients. These analyses should therefore be interpreted with great caution yet are consistent with the main results. Further, CMR data were only available in a small subset of patients with ACS, precluding any definitive conclusions, and should be interpreted as hypothesis-generating. Next, the pooled analyses in *Figure 1* do not account for cohort-specific data properties. However, similar results were observed in the individual cohorts. Finally, drug dosages were not routinely recorded, and there was limited availability of gastrointestinal, hepatic, and host immune system-related parameters in the included cohorts.

## Conclusions

Circulating levels of ImP are linked to cardiometabolic traits and independently predict adverse outcomes in patients with ACS and CCS. Imidazole propionate represents a novel biomarker of cardiometabolic risk and a potential therapeutic target in patients with CAD. Our findings highlight the increasing importance of non-traditional risk factors and may open new avenues for targeting the gut–heart axis to address residual cardiovascular risk.

## Supplementary Material

ehaf661_Supplementary_Data

## Declarations

### Data Availability

The data underlying this article cannot be shared publicly due to data protection regulations related to the different patient cohorts involved in this study. Requests for the data and additional documents related to this study should be made to the corresponding authors.

### Pre-registered Clinical Trial Number

The pre-registered identifiers are ClinicalTrials.gov Identifier NCT01000701 (SPUM-ACS), ClinicalTrials.gov Identifier NCT01529554 (CLEVER-ACS), and German Clinical Trial Register Identifier DRKS00020915 (LipidCardio study).

## References

[1] Vos T, Lim SS, Abbafati C, Abbas KM, Abbasi M, Abbasifard M, et al Global burden of 369 diseases and injuries in 204 countries and territories, 1990-2019: a systematic analysis for the Global Burden of Disease Study 2019. Lancet 2020;396:1204–22. 10.1016/S0140-6736(20)30925-9 33069326 PMC7567026

[2] Nabel EG, Braunwald E. A tale of coronary artery disease and myocardial infarction. N Engl J Med 2012;366:54–63. 10.1056/NEJMra1112570 22216842

[3] Libby P . The changing landscape of atherosclerosis. Nature 2021;592:524–33. 10.1038/s41586-021-03392-8 33883728

[4] Zaman S, Wasfy JH, Kapil V, Ziaeian B, Parsonage WA, Sriswasdi S, et al The lancet commission on rethinking coronary artery disease: moving from ischaemia to atheroma. Lancet 2025;405:1264–312. 10.1016/S0140-6736(25)00055-8 40179933 PMC12315672

[5] Koh A, Molinaro A, Ståhlman M, Khan MT, Schmidt C, Mannerås-Holm L, et al Microbially produced imidazole propionate impairs insulin signaling through mTORC1. Cell 2018;175:947–61.e17. 10.1016/j.cell.2018.09.055 30401435

[6] Venskutonytė R, Koh A, Stenström O, Khan MT, Lundqvist A, Akke M, et al Structural characterization of the microbial enzyme urocanate reductase mediating imidazole propionate production. Nat Commun 2021;12:1347. 10.1038/s41467-021-21548-y 33649331 PMC7921117

[7] Mastrangelo A, Robles-Vera I, Mañanes D, Galán M, Femenía-Muiña M, Redondo-Urzainqui A, et al Imidazole propionate is a driver and therapeutic target in atherosclerosis. Nature 2025. 10.1038/s41586-025-09263-w PMC12408353 40670786

[8] Nageswaran V, Carreras A, Reinshagen L, Beck KR, Steinfeldt J, Henricsson M, et al Gut microbial metabolite imidazole propionate impairs endothelial cell function and promotes the development of atherosclerosis. Arterioscler Thromb Vasc Biol 2025;45:823–39. 10.1161/ATVBAHA.124.322346 40143816 PMC12017598

[9] Koh A, Mannerås-Holm L, Yunn NO, Nilsson PM, Ryu SH, Molinaro A, et al Microbial imidazole propionate affects responses to metformin through p38γ-dependent inhibitory AMPK phosphorylation. Cell Metab 2020;32:643–53.e4. 10.1016/j.cmet.2020.07.012 32783890 PMC7546034

[10] Molinaro A, Bel Lassen P, Henricsson M, Wu H, Adriouch S, Belda E, et al Imidazole propionate is increased in diabetes and associated with dietary patterns and altered microbial ecology. Nat Commun 2020;11:5881. 10.1038/s41467-020-19589-w 33208748 PMC7676231

[11] Sayols-Baixeras S, Dekkers KF, Baldanzi G, Jönsson D, Hammar U, Lin YT, et al Streptococcus species abundance in the gut is linked to subclinical coronary atherosclerosis in 8973 participants from the SCAPIS cohort. Circulation 2023;148:459–72. 10.1161/CIRCULATIONAHA.123.063914 37435755 PMC10399955

[12] Xu Q, Wang W, Li Y, Liu Y, Liu Y. Imidazole propionate in type 2 diabetes mellitus and cardiovascular diseases: a mini review. Front Immunol 2024;15:1454210. 10.3389/fimmu.2024.1454210 39267755 PMC11390552

[13] Molinaro A, Nemet I, Bel Lassen P, Chakaroun R, Nielsen T, Aron-Wisnewsky J, et al Microbially produced imidazole propionate is associated with heart failure and mortality. JACC Heart Fail 2023;11:810 21. 10.1016/j.jchf.2023.03.008 37115134 PMC12512386

[14] Wenzl FA, Bruno F, Kraler S, Klingenberg R, Akhmedov A, Ministrini S, et al Dipeptidyl peptidase 3 plasma levels predict cardiogenic shock and mortality in acute coronary syndromes. Eur Heart J 2023;44:3859–71. 10.1093/eurheartj/ehad545 37632743

[15] Wenzl FA, Kraler S, Ambler G, Weston C, Herzog SA, Räber L, et al Sex-specific evaluation and redevelopment of the GRACE score in non-ST-segment elevation acute coronary syndromes in populations from the UK and Switzerland: a multinational analysis with external cohort validation. Lancet 2022;400:744–56. 10.1016/S0140-6736(22)01483-0 36049493

[16] Davies A, Wenzl FA, Li XS, Winzap P, Obeid S, Klingenberg R, et al Short and medium chain acylcarnitines as markers of outcome in diabetic and non-diabetic subjects with acute coronary syndromes. Int J Cardiol 2023;389:131261. 10.1016/j.ijcard.2023.131261 37574027 PMC13238935

[17] Kraler S, Wenzl FA, Georgiopoulos G, Obeid S, Liberale L, von Eckardstein A, et al Soluble lectin-like oxidized low-density lipoprotein receptor-1 predicts premature death in acute coronary syndromes. Eur Heart J 2022;43:1849–60. 10.1093/eurheartj/ehac143 35567560

[18] Wenzl FA, Luscher TF. Application of a sex-specific GRACE score in practice—authors’ reply. Lancet 2023;401:23. 10.1016/S0140-6736(22)02457-6 36610768

[19] Kraler S, Wenzl FA, Vykoukal J, Fahrmann JF, Shen MY, Chen DY, et al Low-density lipoprotein electronegativity and risk of death after acute coronary syndromes: a case-cohort analysis. Atherosclerosis 2023;376:43–52. 10.1016/j.atherosclerosis.2023.05.014 37285778

[20] Bruno F, Adjibodou B, Obeid S, Kraler SC, Wenzl FA, Akhtar MM, et al Occlusion of the infarct-related coronary artery presenting as acute coronary syndrome with and without ST-elevation: impact of inflammation and outcomes in a real-world prospective cohort. Eur Heart J Qual Care Clin Outcomes 2023;9:564–74. 10.1093/ehjqcco/qcad027 37197909

[21] Winzap PA, Kraler S, Obeid S, Wenzl FA, Templin C, Klingenberg R, et al Initial systolic blood pressure associates with systemic inflammation, myocardial injury, and outcomes in patients with acute coronary syndromes. Eur Heart J Acute Cardiovasc Care 2023;12:437–50. 10.1093/ehjacc/zuad047 37155643

[22] Kraler S, Balbi C, Vdovenko D, Lapikova-Bryhinska T, Camici GG, Liberale L, et al Circulating GDF11 exacerbates myocardial injury in mice and associates with increased infarct size in humans. Cardiovasc Res 2023;119:2729–42. 10.1093/cvr/cvad153 37742057 PMC10757585

[23] Bruno F, Wenzl FA, De Filippo O, Kraler S, Giacobbe F, Roffi M, et al Safety and effectiveness of glycoprotein IIb/IIIa inhibitors in acute coronary syndromes: insights from the SPUM-ACS study. Eur Heart J Cardiovasc Pharmacother 2024;10:391–402. 10.1093/ehjcvp/pvae024 38604747

[24] Narula S, Yusuf S, Chong M, Ramasundarahettige C, Rangarajan S, Bangdiwala SI, et al Plasma ACE2 and risk of death or cardiometabolic diseases: a case-cohort analysis. Lancet 2020;396:968–76. 10.1016/S0140-6736(20)31964-4 33010842 PMC7529405

[25] O'Brien KM, Lawrence KG, Keil AP. The case for case-cohort: an applied epidemiologist's guide to reframing case-cohort studies to improve usability and flexibility. Epidemiology 2022;33:354–61. 10.1097/EDE.0000000000001469 35383643 PMC9172927

[26] König M, Joshi S, Leistner DM, Landmesser U, Sinning D, Steinhagen-Thiessen E, et al Cohort profile: role of lipoproteins in cardiovascular disease-the LipidCardio study. BMJ Open 2019;9:e030097. 10.1136/bmjopen-2019-030097 PMC6731918 31481563

[27] Stähli BE, Klingenberg R, Heg D, Branca M, Manka R, Kapos I, et al Mammalian target of rapamycin inhibition in patients with ST-segment elevation myocardial infarction. J Am Coll Cardiol 2022;80:1802–14. 10.1016/j.jacc.2022.08.747 36049557

[28] Stamatelopoulos K, Mueller-Hennessen M, Georgiopoulos G, Lopez-Ayala P, Sachse M, Vlachogiannis NI, et al Cathepsin S levels and survival among patients with non-ST-segment elevation acute coronary syndromes. J Am Coll Cardiol 2022;80:998–1010. 10.1016/j.jacc.2022.05.055 36049808

[29] Qiu X, Yang J, Hu X, Li J, Zhao M, Ren F, et al Association between hearing ability and cortical morphology in the elderly: multiparametric mapping, cognitive relevance, and neurobiological underpinnings. EBioMedicine 2024;104:105160. 10.1016/j.ebiom.2024.105160 38788630 PMC11140565

[30] Stamatelopoulos K, Mueller-Hennessen M, Georgiopoulos G, Sachse M, Boeddinghaus J, Sopova K, et al Amyloid-β (1-40) and mortality in patients with non-ST-segment elevation acute coronary syndrome: a cohort study. Ann Intern Med 2018;168:855–65. 10.7326/M17-1540 29799975

[31] Wenzl FA, Wang P, Arrigo M, Parenica J, Jones DJL, Bruno F, et al Proenkephalin improves cardio-renal risk prediction in acute coronary syndromes: the KID-ACS score. Eur Heart J 2025;46:38–54. 10.1093/eurheartj/ehae602 39215600 PMC11695896

[32] Grund B, Sabin C. Analysis of biomarker data: logs, odds ratios, and receiver operating characteristic curves. Curr Opin HIV AIDS 2010;5:473–9. 10.1097/COH.0b013e32833ed742 20978390 PMC3157029

[33] Fox KA, Fitzgerald G, Puymirat E, Huang W, Carruthers K, Simon T, et al Should patients with acute coronary disease be stratified for management according to their risk? Derivation, external validation and outcomes using the updated GRACE risk score. BMJ Open 2014;4:e004425. 10.1136/bmjopen-2013-004425 PMC3931985 24561498

[34] Li XS, Obeid S, Klingenberg R, Gencer B, Mach F, Räber L, et al Gut microbiota-dependent trimethylamine N-oxide in acute coronary syndromes: a prognostic marker for incident cardiovascular events beyond traditional risk factors. Eur Heart J 2017;38:814–24. 10.1093/eurheartj/ehw582 28077467 PMC5837488

[35] von Elm E, Altman DG, Egger M, Pocock SJ, Gotzsche PC, Vandenbroucke JP, et al The strengthening the reporting of observational studies in epidemiology (STROBE) statement: guidelines for reporting observational studies. Lancet 2007;370:1453–7. 10.1016/S0140-6736(07)61602-X 18064739

[36] Seldin MM, Meng Y, Qi H, Zhu W, Wang Z, Hazen SL, et al Trimethylamine N-oxide promotes vascular inflammation through signaling of mitogen-activated protein kinase and nuclear factor-κB. J Am Heart Assoc 2016;5:e002767. 10.1161/JAHA.115.002767. 26903003 PMC4802459

[37] Witkowski M, Witkowski M, Friebel J, Buffa JA, Li XS, Wang Z, et al Vascular endothelial tissue factor contributes to trimethylamine N-oxide-enhanced arterial thrombosis. Cardiovasc Res 2022;118:2367–84. 10.1093/cvr/cvab263 34352109 PMC9890461

[38] Zhang X, Li Y, Yang P, Liu X, Lu L, Chen Y, et al Trimethylamine-N-oxide promotes vascular calcification through activation of NLRP3 (nucleotide-binding domain, leucine-rich-containing family, pyrin domain-containing-3) inflammasome and NF-κB (nuclear factor κB) signals. Arterioscler Thromb Vasc Biol 2020;40:751–65. 10.1161/ATVBAHA.119.313414 31941382

[39] Galli M, Niccoli G, De Maria G, Brugaletta S, Montone RA, Vergallo R, et al Coronary microvascular obstruction and dysfunction in patients with acute myocardial infarction. Nat Rev Cardiol 2024;21:283–98. 10.1038/s41569-023-00953-4 38001231

[40] Tang WH, Hazen SL. The gut microbiome and its role in cardiovascular diseases. Circulation 2017;135:1008–10. 10.1161/CIRCULATIONAHA.116.024251 28289004 PMC5354081

