# Supplementary material for: Gut microbiota-derived imidazole propionate predicts cardiometabolic risk in patients with coronary artery disease
Source: Eur Heart J. 2025 Aug 30;47(31):4259–73. doi: 10.1093/eurheartj/ehaf661 (PMC13472685; doi:10.1093/eurheartj/ehaf661)
Supplement: Supplementary file 1 [file ehaf661_supplementary_data.pdf]

-- SUPPLEMENTARY APPENDIX --

Gut microbiota-derived imidazole propionate predicts cardiometabolic risk in patients with coronary artery disease

Florian A. Wenzl,[1-4#*] Peizhi Wang,[1,5,6#] Florian Kahles,[7#] Katharina R. Beck,[8#] Evangelos Giannitsis,[9] Slayman Obeid,[10] Francesco Bruno,[11,12] Xinmin S. Li,[13,14] David Nanchen,[15] Luca Liberale,[16,17] Maria A. Smolle,[18] Victor Schweiger,[19,20] Roland Klingenberg,[21,22] Robert Manka,[23,24] Barbara E. Stähli,[25] Christian Templin,[1,26] Maximilian König,[27] Jinqing Yuan,[6] Mustafa Yildirim,[9] Michael Lehrke,[7] Arnold von Eckardstein,[28] Nikolaus Marx,[7] François Mach,[29] Lorenz Räber,[30] Hugo A. Katus,[9] Elisabeth Steinhagen-Thiessen,[31,32] Ilja Demuth,[32,33] John Deanfield,[34] Peter Libby,[35] Stanley L. Hazen,[13,14,36] Arash Haghikia,[31,37-40†] Ulf Landmesser,[31,37–39†] Fredrik Bäckhed,[8,41–43†] and Thomas F. Lüscher,[1,12,44,45†*] on behalf of the SPUM-ACS, CLEVER-ACS, Heidelberg II-ACS, and LipidCardio Investigators

1 Center for Molecular Cardiology, University of Zürich, Schlieren, Switzerland.
2 National Health Service England, Wellington House, 133-135 Waterloo Road, London SE1 8UG, United Kingdom.
3 Department of Cardiovascular Sciences, University of Leicester, University Road LE1 7RH, Leicester, United Kingdom.
4 Department of Clinical Sciences, Karolinska Institute, 171 77 Stockholm, Sweden.
5 Department of Cardiology, State Key Laboratory of Complex Severe and Rare Diseases, Peking Union Medical College Hospital, Chinese Academy of Medical Sciences and Peking Union Medical College, Beijing, China.
6 Department of Cardiology, National Clinical Research Center for Cardiovascular Diseases, Fuwai Hospital, National Center for Cardiovascular Diseases, Chinese Academy of Medical Sciences and Peking Union Medical College, Beijing, China.
7 Department of Internal Medicine I, University Hospital Aachen, Pauwelsstraße 30, 52074 Aachen, Germany.
8 Wallenberg Laboratory, Department of Molecular and Clinical Medicine and Sahlgrenska Center for Cardiovascular and Metabolic Research, University of Gothenburg, 413 45 Gothenburg, Sweden.
9 Department of Cardiology, University Hospital Heidelberg, Im Neuenheimer Feld 410, 69120 Heidelberg, Germany.
10 Division of Cardiology, Department of Medicine, Basel Cantonal Hospital, Basel, Switzerland.
11 Division of Cardiology, Cardiovascular and Thoracic Department, Molinette Hospital, Città della Salute e della Scienza, Corso Bramante 88, 10126 Turin, Italy.
12 Heart Division, Royal Brompton and Harefield Hospitals, Sydney Street, London, United Kingdom.
13 Departments of Cardiovascular and Metabolic Sciences, and Cardiovascular Medicine, Lerner Research Institute, Cleveland Clinic, Cleveland, OH, USA.
14 Center for Microbiome and Human Health, Lerner Research Institute, Cleveland Clinic, Cleveland, OH, USA.
15 Center for Primary Care and Public Health (Unisanté), University of Lausanne, Lausanne, Switzerland.
16 First Clinic of Internal Medicine, Department of Internal Medicine, University of Genoa, 6 Viale Benedetto XV, 16132, Genoa, Italy.
17 IRCCS Ospedale Policlinico San Martino, Genoa - Italian Cardiovascular Network, 10 Largo Benzi, 16132, Genoa, Italy.
18 Comprehensive Cancer Center Graz, Medical University of Graz, Neue Stiftingtalstraße 6, 8010 Graz, Austria.
19 Department of Cardiology, University Hospital Zurich, Rämistrasse 100, 8091 Zurich Switzerland.
20 Department of Cardiology, Angiology and Intensive Care Medicine, Deutsches Herzzentrum der Charité, Augustenburger Platz 1, 13353 Berlin, Germany.
21 Department of Cardiology, Krankenhaus Nordwest GmbH, Steinbacher Hohl 2-26, 60488 Frankfurt, Germany.
22 Department Medical Clinic I, Cardiology and Angiology, Justus-Liebig-University, Giessen, Germany
23 Department of Cardiology, University Heart Center, University Hospital Zurich, Zurich, Switzerland.
24 Institute of Diagnostic and Interventional Radiology, University Hospital Zurich, University of Zurich, Zurich, Switzerland.
25 Department of Cardiology, University Heart Center, University Hospital Zurich, University of Zurich, Zurich Switzerland.
26 Department of Internal Medicine B, University Medicine Greifswald, Greifswald, Germany.
27 Department of Internal Medicine and Geriatrics, University Medicine Greifswald, Greifswald, Germany.
28 Institute of Clinical Chemistry, University Hospital Zurich and University of Zurich, Zurich, Switzerland.
29 Department of Cardiology, Geneva University Hospital, Geneva, Switzerland.
30 Department of Cardiology, Cardiovascular Center, University Hospital Bern, Bern, Switzerland.
31 Friede Springe-Cardiovascular Prevention Center at Charité, Charité-Universitätsmedizin Berlin, Berlin, Germany.
32 Department of Endocrinology and Metabolic Diseases (including Division of Lipid Metabolism), Charité-Universitätsmedizin Berlin, corporate member of the Freie Universität Berlin and Humboldt-Universität zu Berlin, Berlin, Germany.
33 BCRT - Berlin Institute of Health Center for Regenerative Therapies, Charité - Universitätsmedizin Berlin, Berlin, Germany.
34 Institute of Cardiovascular Science, University College London, London United Kingdom.
35 Division of Cardiovascular Medicine, Department of Medicine, Brigham and Women's Hospital, Harvard Medical School, Boston, MA, USA.
36 Department of Cardiovascular Medicine, Heart, Vascular and Thoracic Institute, Cleveland Clinic, Cleveland, OH, USA.
37 Department of Cardiology, Angiology and Intensive Care Medicine, Deutsches Herzzentrum der Charité, Campus Benjamin Franklin, Berlin, Germany.
38 German Center for Cardiovascular Research (DZHK), Partner Site Berlin, Berlin, Germany.
39 Berlin Institute of Health at Charité-Universitätsmedizin Berlin, Berlin, Germany.
40 University Hospital St. Josef-Hospital Bochum, Cardiology and Rhythmology, Ruhr University Bochum, Bochum, Germany.
41 Department of Clinical Physiology, Region Västra Götaland, Sahlgrenska University Hospital, Gothenburg, Sweden.
42 Novo Nordisk Foundation Microbiome Health Initiative, Hellerup, Denmark.
43 National Food Institute, Technical University of Denmark, Kongens Lyngby, Denmark
44 National Heart and Lung Institute, Imperial College London, Dovehouse Street, London SW3 5NP, United Kingdom.
45 School of Cardiovascular Medicine and Sciences, King's College London, Strand, London WC2R 2LS, United Kingdom.

#Contributed equally *Corresponding authors †Jointly directed the study

Running Title: Imidazole propionate and cardiometabolic risk

Correspondence:

Florian A. Wenzl, MD FESC
E-mail: florian.wenzl@uzh.ch
Phone: +41 76 749 42 50

Thomas F. Lüscher, MD FRCP FESC
E-mail: cardio@tomluescher.ch
Phone: +41 44 250 40 97

Table of Contents

Table S1: Baseline characteristics of patients with ACS (Swiss ACS cohort) stratified by imidazole propionate quartiles ... 6
Table S2: Treatment characteristics of patients with ACS (Swiss ACS cohort) stratified by imidazole propionate quartiles 9
Table S3: Baseline characteristics of patients in case-cohort analysis of German ACS cohort ... 11
Table S4: Treatment characteristics of patients in case-cohort analysis of German ACS cohort ... 13
Table S5: Baseline characteristics of patients in case-cohort analysis of German ACS cohort stratified by imidazole propionate quartiles ... 14
Table S6: Treatment characteristics of patients in case-cohort analysis of German ACS cohort stratified by imidazole propionate quartiles ... 17
Table S7: Baseline characteristics of patients in Swiss CMR cohort ... 19
Table S8: Treatment characteristics of patients in Swiss CMR cohort ... 21
Table S9: Baseline characteristics of patients with CCS (German CCS cohort) stratified by imidazole propionate quartiles ... 22
Table S10: Treatment characteristics of patients with CCS (German CCS cohort) stratified by imidazole propionate quartiles ... 25
Table S11: Baseline characteristics of patients with ACS (Swiss ACS cohort and German ACS cohort) and CCS (German CCS cohort) in fully adjusted model ... 27
Table S12: Treatment characteristics of patients with ACS (Swiss ACS cohort and German ACS cohort) and CCS (German CCS cohort) in fully adjusted model ... 30
Table S13: Correlation of imidazole propionate, histidine, and urocanate levels with baseline characteristics in patients with CAD (pooled data from Swiss ACS cohort and German CCS cohort) ... 32
Table S14: Correlation of imidazole propionate, histidine, and urocanate levels with coronary microvascular obstruction at 30 days after primary percutaneous coronary intervention ... 35
Table S15: Association of circulating imidazole propionate with MACE ... 36
Table S16: Association of circulating imidazole propionate with mortality ... 38
Table S17: Association of circulating imidazole propionate with MACE in subgroups ... 40
Table S18: Association of circulating imidazole propionate with mortality in subgroups ... 42
Table S19: Sensitivity analysis using cohort-specific quartile thresholds ... 45
Table S20: Summary of missing data according to study cohort ... 47
Table S21: Association of imidazole propionate with MACE beyond TMAO ... 49
Table S22: Association of imidazole propionate with mortality beyond TMAO ... 50
Figure S1: Study flow chart ... 51
Figure S2: Analytical flow chart ... 52
Figure S3: Imidazole propionate levels across different times of the day in Swiss ACS cohort ... 53
Figure S4: Imidazole propionate levels across different times of the day in German CCS cohort ... 54
Figure S5: Imidazole propionate distribution stratified by clinical subgroup ... 55
Figure S6: Histidine and urocanate levels in patients with CAD (pooled data from Swiss ACS cohort and German CCS cohort) ... 56
Figure S7: Imidazole propionate levels in patients with ACS (Swiss ACS cohort) ... 57
Figure S8: Histidine and urocanate levels in patients with ACS (Swiss ACS cohort) ... 58
Figure S9: Imidazole propionate levels in patients with CCS (German CCS cohort) ... 59
Figure S10: Histidine and urocanate levels in patients with CCS (German CCS cohort) ... 60
Figure S11: Association of imidazole propionate levels with differential blood count in patients with ACS (Swiss ACS cohort) ... 61
Figure S12: Association of histidine and urocanate levels and coronary microvascular obstruction in patients with ACS . 62
Figure S13: Combined association of imidazole propionate and TMAO with mortality in patients with ACS (Swiss ACS cohort) and in patients with CCS (German CCS cohort) ... 63
Figure S14: Rehospitalisations according to imidazole propionate levels ... 64
Figure S15: STROBE Statement ... 65
References ... 67

Description of the statistical analyses (detailed)

Patient cohorts

We aimed to encompass the full spectrum of coronary artery disease (CAD) including patients presenting with acute and stable clinical conditions. We used prospective acute coronary syndrome (ACS) and chronic coronary syndrome (CCS) cohorts from Switzerland and Germany (***Suppl. Figure 1***).

The SPUM-ACS Biomarker Study (ClinicalTrials.gov Identifier: NCT01000701; i.e., Swiss ACS cohort) constitutes an investigator-initiated, prospective, multicenter cohort study. It included patients who presented with ACS at one of the participating university hospitals in Switzerland between Dec 2009 and Dec 2017. The study is characterized by a rigorous methodological framework with phenotypic profiling, a comprehensive biobank, and clinical follow-up. Patients were included if aged ≥ 18 years, presenting with the main diagnosis of ACS, and having been admitted within 5 days (preferentially within 72 h) from onset of symptoms.[1] Patients who were unable to provide informed consent, had a severe physical disability, or had a non-cardiac life expectancy < 12 months were excluded.[2] Ethylenediaminetetraacetic acid (EDTA) blood samples were obtained at baseline prior to any intervention (time point 1).[1 2] Physicians were advised to adhere to guideline-directed treatment regimens.

The CLEVER-ACS (Controlled Level Everolimus in Acute Coronary Syndromes, ClinicalTrials.gov Identifier: NCT01529554; i.e., Swiss cardiac magnetic resonance [CMR] cohort) study is a prospective, randomized, multicenter, international, double-blinded, placebo-controlled trial, originally designed to assess the effects of everolimus on myocardial infarction (MI) size of patients with ACS via cardiac magnetic resonance (CMR) imaging.[3,4] Patients with ST-elevation MI (STEMI) owing to proximal occlusion of a coronary artery were screened between 0 and 5 days following percutaneous coronary intervention (PCI) in a 1:1 ratio. Inclusion criteria were: age between 18 and 90 years; MI as first event; chest pain over at least 10 minutes; ST-segment elevations > 1mm in a t least 2 adjacent leads; new left bundle branch block (LBBB) or posterior MI with ST-segment depression > 1 mm in at least 2 adjacent leads; angiographic signs of occlusion of either the left circumflex coronary artery (LCX), the right coronary artery (RCA), the left anterior descending coronary artery (LAD), or the mid segment of the LAD or RCA when the latter reached the left ventricular apex; PCI with implantation of a drug-eluting stent in the culprit lesion within 24 hours from symptom onset; ability and willingness to provide informed consent and adhere to the study protocol, as described previously.[3] Patients were excluded in case of contraindications to receive the study intervention, contraindications to CMR or contrast agent administered during CMR, use of immunosuppressants up to 4 weeks prior to index STEMI, mechanical complications related to MI, multivessel coronary artery disease necessitating revascularization strategies of a non-culprit artery during the trial period, a CI scheduled within 30 days, major elective surgery planned during study duration, estimated glomerular filtration rate (eGFR) < 30 mL/min, known malignancy (except for cured malignancy or in remission for > 5 years), suspected/known noncompliance, substance abuse (drugs, alcohol), known chronic infection, pregnancy or lactation, participation in another trial with an investigational product within 30 days or 5 terminal half-lives, patients of childbearing age not willing to use reliable contraception for the duration of the study, a positive PCR (polymerase chain reaction) test for severe acute respiratory syndrome-coronavirus-2 (SARS-CoV2), or, since Sept 2020, at least one positive answer to the symptoms/contact SARS-CoV-2 questionnaire.[3] Active recruitment took place between Nov 2014 and Oct 2021 at 8 participating centers in Switzerland and Germany. Overall, 150 patients could be randomized (75:75). Patients either received oral everolimus or a matching placebo, each for 5 days, as described previously.[3] All patients received drug-eluting stents and evidence-based concomitant therapies. Blood samples were obtained a baseline and at discharge. In the current study, all patients with available blood sample and information on CMR-assessed coronary microvascular obstruction were included (n = 60; 8 co-enrolled in SPUM-ACS) und blood samples from the earliest available time point during hospitalisation was used. When calculating the overall patient count across all cohorts involved in this study, we did not account for potential co-enrolment of patients in CLEVER-ACS and SPUM-ACS.

The Heidelberg II-ACS study (i.e., German ACS cohort) enrolled consecutive patients treated for ACS at the Heidelberg University Hospital, Heidelberg, Germany, between Jun 2009 ad Apr 2014.[5] Two independent cariologists retrospectively adjudicated the diagnosis of NSTE-ACS using all available clinical information. In case of disagreement, a third independent cardiologists was involved.[5] Myocardial infarctions were diagnosed using high-sensitivity cardiac troponin T (hs-cTnT), aligning with the universal MI definition (i.e., increase or decrease in hs-cTNT levels, at least one hs-cTnT > 99th percentile, clinical evidence of ischaemia).[5,6] Blood samples were obtained at baseline.[5] Treating physicians were advised to administer guidelines-based therapy to patients.[5]

The LipidCardio study (German Clinical Trial Register Identifier: DRKS00020915; German CCS cohort) included patients ≥ 18 years undergoing cardiac catheterization between Oct 2016 and Mar 2018 at the Department of Cardiology, Campus Benjamin Franklin, Charité-Universitätsmedizin Berlin, Berlin, Germany. Patients with troponin-positive ACS and inability to provide informed consent were excluded.[7] Blood samples (EDTA) were obtained at time of cardiac catheterization (after administration of heparin), either comprising arterial blood drawn from the radial or femoral artery sheath or venous blood drawn from a peripheral intravenous access.[7] EDTA blood samples were immediately processed and stored at -80°C until further use.[7] Patient demographics and information on medications at admission, alcohol/drug use, smoking habit, physical ability, family history, previous diagnoses of chronic conditions, previous angiographic findings and revascularization strategies were documented.[7] Further, patients underwent a screening test for cognitive impairment (i.e., mini-mental state examination [MMSE]), as described previously.[7] Patients with confirmed CCS, defined as angiographic evidence of obstructive CAD (≥50% stenosis), were included in the current study.

Metabolite measurements

ImP and its precursor molecules, histidine and trans-urocanate, were measured centrally using ultra-high performance liquid chromatography coupled to tandem mass spectrometry. We analysed EDTA plasma samples in SPUM-ACS and German CCS and serum samples in German ACS and CLEVER-ACS.

The cohorts were measured at University of Gothenburg, Gothenburg, Sweden using an internal standard (ImP-$^{13}C_3$) for sample preparation. Samples were prepared and analyzed using a previously established method with minor modifications. In brief, 25 µL of plasma samples were extracted with 6 volumes of acetonitrile containing internal standards (100 nM ImP-$^{13}C_3$ and urocanate-$^{13}C_3$, AstraZeneca, Cambridge, United Kingdom and 1 µmol/L L-histidine D_5, $^{15}N_3$, Cambridge Isotope Laboratories, Inc. Andover, MA) before drying under a nitrogen stream. The samples were then reconstituted in 5% HCl (37%) in 1-butanol for n-butyl ester derivatization at 70°C for 40 min dried and finally reconstituted in 150 µL water:acetonitrile (9:1). ImP, urocanate and histidine levels were quantified using multiple reaction monitoring of the transitions 197.2/81.2, 195.2/93.0, 213.2/110.1 and internal standards of the transitions 200.2/82.0, 198.2/95.0 and 220.3/118.1. Investigators performing the measurements were blinded to sample allocation and patient data other than the deidentified barcode label.

In a subgroup of patients from the SPUM-ACS and in patients from German CCS, circulating levels of the trimethylamine N-oxide (TMAO) were measured, as described previously.[8,9]

Case-cohort analyses

Given the resource-intensive nature of the measurements, patients from the prospective German ACS cohort were analysed using a case-cohort design, which permits unbiased assessment of exposure–outcome relationships, while reducing costs incurred by the measurement. The design of case-cohort analyses has been reported previously.[10,11] From the pool of eligible participants, we randomly selected 10% of patients (i.e., the subcohort). This subcohort contains patients with an incident event ('cases') and patients without an incident event of interest ('noncases'). We then also selected all individuals with incident events who were not part of the subcohort. The final selection of patients (n = 250) who were included in the analyses consists of the subcohort and participants with incident events outside the subcohort. Therefore, patients included in the analyses fall into three groups, which are attributed different weights on weighted Cox proportional hazards regression analysis: cases in subcohort, non-cases in subcohort, and cases not in subcohort. Cases in the sub-cohort receive a weight of 1/10% from the start of follow-up until just before their event time and then a weight of 1 at the exact time of their event; non-cases in the sub-cohort are weighted as 1/10% for their entire follow-up time; and cases not in the sub-cohort receive a weight of 1 from just before their event time to their event.[11]

Follow-up and outcomes

Major adverse cardiovascular events (MACE), defined as the first occurrence of a composite of death, nonfatal myocardial infarction, or nonfatal stroke after admission, were the primary endpoint. Mortality was analysed as an additional endpoint. An event horizon of 1 year (365 days) and of 3 years (1095 days), was used in ACS and CCS cohorts, respectively. In the Swiss ACS cohort (SPUM-ACS), patients with ACS were followed up at three time points (hospital discharge; 30 days [phone call], 1 year [clinical visit]). Specifically trained study personnel at each study center collected baseline and event data via a centralized web-based data entry system (CARDIOBASE, Clinical Trial Unit and Department of Cardiology, University Hospital Bern, Bern, Switzerland, and Webspirit Systems GmbH, Ulm, Germany).[1] An independent clinical endpoint committee consisting of three certified expert cardiologists (who were blinded to patients' baseline characteristics) used pre-specified adjudication forms to assess fatal events. A committee consisting of expert cardiologists from each study center monitored data collection. In the German ACS cohort, patients were followed up via telephone call and/or a questionnaire forwarded by postal or e-mail, as previously described.[5] All deaths (retrieved via local residents' registry) and nonfatal adverse cardiovascular events were recorded.[5] In the German CCS cohort, patients were followed-up via phone call or a questionnaire sent by post at 3 years after study enrolment[7] to assess nonfatal adverse cardiovascular events.[7] Mortality was retrieved from the obligatory registry office.[7]

Association of imidazole propionate with patient characteristics

Continuous variables were compared with the Mann-Whitney U test for comparisons across two groups and the Kruskal–Wallis test for comparisons across three or more groups. Trends across independent groups were tested using the Jonckheere-Terpstra test. We used partial Spearman rank-correlation to examine the association between pairs of continuous variables while adjusting for additional variables.[12] For comparison, the precursor molecules of ImP, histidine and urocanate, were analysed following a similar approach. Patients from CLEVER-ACS and from the case-cohort analysis of German ACS were not included in any analyses on the association of ImP and its precursors with patient characteristics, given the specific selection criteria of these populations. Body surface area was calculated according to Du Bois and Du Bois.[13]

Predictive value of imidazole propionate

The association of circulating ImP with clinical outcomes was studied in complete case Cox proportional hazards regression models. We used restricted cubic splines with 3 knots placed at the at the 10th, 50th, and 90th percentiles across ImP distribution to produce a smooth curve and to investigate a potential nonlinear association of continuous ImP values with the outcomes. We compared Cox models with ImP modelled as an untransformed continuous variable to those with ImP modelled as RCS term and tested for a nonlinear relationship between ImP and log(HR) using the likelihood ratio test. We observed a nonlinear association between ImP and the main outcomes in the smoothed plot of log(HR) versus ImP. We log2-transformed ImP values to achieve a linear fit (see ***Figure 3 B***), i.e., relative effect estimates correspond to a doubling in ImP levels.[1,14,15] In addition, patients were divided according to ImP quartile (Q) for analyses on a categorical scale.[14,16] Quartile cut-offs were based on the ImP distribution in the largest cohort

(i.e., SPUM-ACS) and applied to all cohorts: Q1–Q2 9.4 nM; Q2–Q3 14.3 nM; Q3–Q4 24.4 nM. In a sensitivity analysis, cohort-specific ImP cut offs were explored. Multivariable analyses were conducted using a stepwise approach adjusting for predefined covariables, based on sample size and clinical considerations.

In the Swiss ACS cohort (SPUM-ACS), the fully adjusted model included age, sex, diabetes, high-sensitivity troponin T (hs-TnT), high-sensitivity C-reactive protein (hs-CRP), revascularisation, ACS type, body mass index (BMI) and estimated glomerular filtration rate (eGFR). Due to the smaller sample size and missingness, the fully adjusted models in the German ACS cohort included included age, sex, diabetes, high-sensitivity troponin T (hs-TnT), high-sensitivity C-reactive protein (hs-CRP), revascularisation, ACS type, and estimated glomerular filtration rate (eGFR) and in the German CCS cohort age, sex, diabetes, high-sensitivity C-reactive protein (hs-CRP), revascularisation, body mass index (BMI) and estimated glomerular filtration rate (eGFR). Analyses on mortality in patients with ACS were additionally adjusted for the GRACE 2.0 score for 1-year mortality.[17] Moreover, we evaluated the predictive value of circulating ImP when additionally adjusting for its precursor molecules histidine and urocanate (***Tables S15 and S16***). Furthermore, we additionally accounted for ethnicity (only available in German CCS cohort), lipoprotein(a) [Lp(a)] levels, history of chronic liver disease, and leukocyte count in the Swiss ACS and in the German CCS cohorts. The proportional hazards assumption was checked in the fully adjusted model using Schoenfeld residuals. In case of non-proportional hazards respective variables were included as stratification variables in the final models, for which no major violations were observed. We did several subgroup analyses in the Swiss ACS and the German CCS cohorts to assess the consistency of the results with respect to different population characteristics including sex, age, diabetes, proton pump inhibitor use, and dietary habits (only available in German CCS cohort). In the German CCS cohort, dietary habits were recorded using a questionnaire on the frequency of the consumption of fruit and vegetables (including apples, tomatoes, carrots, grapefruit, brussels sprouts, banana, cauliflower, ginger, tomatoes, curly kale, orange, red cabbage, apple, potatoes, asparagus, and carrots). The frequency at which each fruit and vegetable was consumed was assigned a score ranging from 1 (i.e., lowest frequency) to 5 (i.e., highest frequency) and the median frequency across all fruit and vegetables was calculated. We performed subgroup analyses in patients above and below the median fruit and vegetable consumption score. In a subset of patients from the Swiss ACS cohort with TMAO levels[8,18] and in the German CCS cohort the incremental predictive value of ImP beyond TMAO was assessed by additionally adjusting for TMAO. Furthermore, we formed TMAO quartiles (Q) in each cohort and analysed joint groups of high (≥ Q4 cut-off) *vs.* low (< Q4 cut-off) levels of ImP and TMAO yielding four strata: Low ImP/ Low TMAO, Low ImP/High TMAO, High ImP/Low TMAO, and High ImP/High TMAO. Variable importance was visualized as partial χ^2 minus degrees of freedom in the fully adjusted model with log-transformed continuous variables without stratification variables.

Software packages
The software environment in R for the performed analyses was created by the R packages: *hmisc, dplyr, readxl, stringr, ggplot2, ggsci, ggpubr, scales, ppcor, reshape2, purrr, survival, survminer, lubridate, tidyr, rms, broom, forestplot, tableone, gtsummary, smoothHR, and clinfun.*

Table S1: Baseline characteristics of patients with ACS (Swiss ACS cohort) stratified by imidazole propionate quartiles

	**All Patients (*n* = 4318)**	**ImP Quartile 1 < 9.4 nM (*n* = 1080)**	**ImP Quartile 2 9.4 – 14.3 nM (*n* = 1082)**	**ImP Quartile 3 14.3 – 24.4 nM (*n* = 1076)**	**ImP Quartile 4 > 24.4 nM (*n* = 1080)**
Age (years)	63 (54–73)	59 (52–68)	62 (53–70)	64 (54–73)	68 (59–78)
Female	881/4318 (20.4%)	317/1080 (29.4%)	187/1082 (17.3%)	160/1076 (14.9%)	217/1080 (20.1%)
BMI (kg/m²)	26.6 (24.2–29.4)	26.3 (24.0–28.7)	26.5 (24.2–29.3)	26.6 (24.3–29.7)	27.0 (24.2–30.1)
Body surface area (m²)	1.9 (1.8–2.1)	1.9 (1.8–2.0)	1.9 (1.8–2.1)	1.9 (1.8–2.1)	1.9 (1.8–2.1)
Heart rate (bpm)	75 (66–86)	75 (65–85)	75.0 (66–86)	75.0 (65–85)	77 (67–88)
Systolic blood pressure (mm Hg)	128 (112–143)	127 (111–142)	127 (112–142)	128 (113–145)	129 (113–144)
Signs and symptoms of heart failure (ACS: Killip class, CCS: NYHA class)					
I	3682/4204 (87.6%)	955/1045 (91.4%)	941/1052 (89.4%)	928/1051 (88.3%)	858/1056 (81.2%)
II	343/4204 (8.2%)	64/1045 (6.1%)	77/1052 (7.3%)	80/1051 (7.6%)	122/1056 (11.6%)
III	85/4204 (2.0%)	13/1045 (1.2%)	13/1052 (1.2%)	20/1051 (1.9%)	39/1056 (3.7%)
IV	94/4204 (2.2%)	13/1045 (1.2%)	21/1052 (2.0%)	23/1051 (2.2%)	37/1056 (3.5%)
Smoking	1649/4254 (38.8%)	458/1071 (42.8%)	438/1069 (41.0%)	400/1054 (38.0%)	353/1060 (33.3%)
Medical history					
Diabetes	753/4318 (17.4%)	137/1080 (12.7%)	133/1082 (12.3%)	188/1076 (17.5%)	295/1080 (27.3%)
Prediabetes[#]	630/2153 (29.3%)	136/493 (27.6%)	169/514 (32.9%)	176/539 (32.7%)	149/607 (24.5%)
Hypertension	2413/4316 (55.9%)	522/1080 (48.3%)	534/1082 (49.4%)	627/1074 (58.4%)	730/1080 (67.6%)
Hyperlipoproteinemia	2757/4317 (63.9%)	685/1080 (63.4%)	704/1082 (65.1%)	680/1076 (63.2%)	688/1079 (63.8%)
Myocardial infarction	524/4310 (12.2%)	104/1079 (9.6%)	113/1079 (10.5%)	132/1073 (12.3%)	175/1079 (16.2%)
Family history of CAD	1056/4259 (24.8%)	306/1068 (28.7%)	280/1069 (26.2%)	239/1058 (22.6%)	231/1064 (21.7%)
Prior PCI	644/4317 (14.9%)	117/1080 (10.8%)	147/1082 (13.6%)	161/1076 (15.0%)	219/1079 (20.3%)
Prior CABG	169/4318 (3.9%)	23/1080 (2.1%)	32/1082 (3.0%)	38/1076 (3.5%)	76/1080 (7.0%)

Cerebrovascular disease	160/4318 (3.7%)	31/1080 (2.9%)	34/1082 (3.1%)	37/1076 (3.4%)	58/1080 (5.4%)
Peripheral artery disease	251/4318 (5.8%)	51/1080 (4.7%)	50/1082 (4.6%)	48/1076 (4.5%)	102/1080 (9.4%)
Heart failure	54/4316 (1.3%)	5/1080 (0.5%)	11/1081 (1.0%)	13/1075 (1.2%)	25/1080 (2.3%)
Chronic liver disease	25/4318 (0.6%)	3/1080 (0.3%)	6/1082 (0.6%)	6 /1076 (0.6%)	10/1080 (0.9%)
Dialysis	21/4318 (0.5%)	0/1080 (0.0%)	1/1082 (0.1%)	2/1076 (0.2%)	18/1080 (1.7%)
Clinical chemistry and haematology					
Leukocyte count (G/L)	9.6 (7.4–12.2)	9.2 (7.2–11.6)	9.7 (7.6–12.1)	9.8 (7.5–12.5)	9.7 (7.7–12.4)
hs-CRP (mg/L)	3 (1–7)	2 (1–6)	2 (1–6)	3 (1–7)	4 (1–14)
Total cholesterol (mg/dL)	190 (159–220)	197 (168–227)	191 (166–220)	186 (159–217)	175 (143–209)
LDL-C (mg/dL)	120 (92–149)	128 (102–155)	124 (97–150)	118 (91–146)	109 (79–140)
HDL-C (mg/dL)	44 (36–53)	45 (37–55)	45 (38–54)	43 (36–53)	43 (35–51)
Lipoprotein (a) (mg/dL)	6.4 (2.5–21.8)	7.6 (2.5–23.4)	8.2 (2.5–23.6)	5.4 (2.5–16.7)	6.1 (2.5–22.2)
Triglycerides (mg/dL)	88 (60–136)	89 (56–140)	88 (60–132)	88 (61–135)	89 (61–137)
HbA1c (%)	5.8 (5.5–6.3)	5.6 (5.4–6.0)	5.7 (5.5–6.1)	5.8 (5.5–6.3)	5.9 (5.6–6.7)
Glucose (mmol/L)	6.4 (5.6–7.9)	6.3 (5.5–7.6)	6.3 (5.6–7.6)	6.5 (5.6–8.0)	6.7 (5.7–8.4)
NT-proBNP (ng/L)	334 (108–1158)	237 (81–696)	259 (90–838)	324 (119–1117)	660 (171–2378)
hs-TnT (ng/L)	196 (59–642)	147 (51–512)	207 (64–617)	192 (56–629)	247 (70–823)
Creatinine (mg/dL)	0.9 (0.7–1.0)	0.8 (0.7–0.9)	0.9 (0.8–1.0)	0.9 (0.8–1.1)	1.0 (0.8–1.2)
eGFR (mL/min/1.73m^2)	93 (77–103)	99 (89–107)	95 (83–104)	91 (75–102)	80 (58–97)
ImP metabolism					
ImP (nM)	14 (9–24)	7 (6–8)	12 (11–13)	18 (16–21)	39 (30–60)
Urocanate (nM)	44 (30–66)	41 (28–60)	44 (30–66)	43 (30–66)	48 (31–71)
Histidine (µM)	66 (57–76)	65 (56–75)	66 (57–76)	67 (58–77)	66 (57–76)
Baseline medication					
Aspirin	1230/2779 (44.3%)	250/619 (40.4%)	279/636 (43.9%)	304/694 (43.8%)	397/830 (47.8%)
P2Y12 inhibitor	321/1915 (16.8%)	53/440 (12.0%)	75/434 (17.3%)	77/470 (16.4%)	116/571 (20.3%)

Beta-blocker	978/2779 (35.2%)	183/619 (29.6%)	189/636 (29.7%)	267/694 (38.5%)	339/830 (40.8%)
ACE inhibitor/ARB	1475/2759 (53.5%)	289/613 (47.1%)	321/632 (50.8%)	374/691 (54.1%)	491/823 (59.7%)
Vitamin K antagonist/DOAC	171/2777 (6.2%)	23/619 (3.7%)	27/635 (4.3%)	51/694 (7.3%)	70/829 (8.4%)
Diuretic	674/2773 (24.3%)	118/619 (19.1%)	126/635 (19.8%)	145/692 (21.0%)	285/827 (34.5%)
Insulin	214/2777 (7.7%)	37/619 (6.0%)	40/635 (6.3%)	46/694 (6.6%)	91/829 (11.0%)
Oral antidiabetics	503/2777 (18.1%)	96/619 (15.5%)	93/635 (14.6%)	131/694 (18.9%)	183/829 (22.1%)
Immunosuppressive drugs	119/2777 (4.3%)	23/619 (3.7%)	22/635 (3.5%)	22/694 (3.2%)	52/829 (6.3%)
PPI	643/2777 (23.2%)	108/619 (17.4%)	136/635 (21.4%)	154/694 (22.2%)	245/829 (29.6%)

Categorical data are shown as numbers and valid percentages (%). Continuous data are presented as median and interquartile range (IQR). ACE, angiotensin-converting enzyme; ACS, acute coronary syndrome; ARB, angiotensin II receptor blocker; BMI, body mass index; CABG, coronary artery bypass grafting; CAD, coronary artery disease; CCS, chronic coronary syndrome; DOAC, direct oral anticoagulant; eGFR, estimated glomerular filtration rate; HbA1c, hemoglobin A1c; HDL-C, high-density lipoprotein cholesterol; hs-CRP, high-sensitivity C-reactive protein; hs-TnT, high-sensitivity troponin T; ImP, imidazole propionate; LDL-C, low-density lipoprotein cholesterol; NT-proBNP, N-terminal pro B-type natriuretic peptide; NYHA, New York Heart Association; PCI, percutaneous coronary intervention; PPI, proton pump inhibitor. *Values refer to the subcohort #defined as HbA1c of 5.7–6.4% in individuals without a history of diabetes.

Table S2: Treatment characteristics of patients with ACS (Swiss ACS cohort) stratified by imidazole propionate quartiles

	**All Patients (*n* = 4318)**	**ImP Quartile 1 < 9.4 nM (*n* = 1080)**	**ImP Quartile 2 9.4 – 14.3 nM (*n* = 1082)**	**ImP Quartile 3 14.3 – 24.4 nM (*n* = 1076)**	**ImP Quartile 4 > 24.4 nM (*n* = 1080)**
Revascularization strategy during baseline visit					
PCI	3949/4318 (91.5%)	1010/1080 (93.5%)	1000/1082 (92.4%)	972/1076 (90.3%)	971/1080 (89.9%)
CABG	127/4318 (2.9%)	19/1080 (1.8%)	33/1082 (3.0%)	34/1076 (3.4%)	41/1080 (3.8%)
Symptom-to-door time (minutes)	180 (105–420)	186 (105–476)	175 (105–383)	180 (100–410)	193 (105–445)
No. of affected vessels†					
One	3371/4095 (82.3%)	885/1030 (85.9%)	866/1035 (83.7%)	816/1010 (80.8%)	804/1020 (78.8%)
Two	563/4095 (13.7%)	119/1030 (11.6%)	124/1035 (12.0%)	158/1010 (15.6%)	162/1020 (15.9%)
Three	161/4095 (3.9%)	26/1030 (2.5%)	45/1035 (4.3%)	36/1010 (3.6%)	54/1020 (5.3%)
Left ventricular ejection fraction (%)					
> 50	1380/2725 (50.6%)	376/686 (54.8%)	350/692 (50.6%)	353/695 (50.8%)	301/652 (46.2%)
50–40	858/2725 (31.5%)	228/686 (33.2%)	194/692 (28.0%)	218/695 (31.4%)	218/652 (33.4%)
40–30	314/2725 (11.5%)	56/686 (8.2%)	103/692 (14.9%)	82/695 (11.8%)	73/652 (11.2%)
< 30	173/2725 (6.3%)	26/686 (3.8%)	45/692 (6.5%)	42/695 (6.0%)	60/652 (9.2%)
Discharge medication					
Aspirin	4233/4273 (99.1%)	1072/1077 (99.5%)	1068/1077 (99.2%)	1051/1064 (98.8%)	1042/1055 (98.8%)
P2Y12 inhibitor	4070/4163 (97.8%)	1037/1056 (98.2%)	1020/1046 (97.5%)	1016/1032 (98.4%)	997/1029 (96.9%)
Beta-blocker	3339/4269 (78.2%)	813/1075 (75.6%)	863/1077 (80.1%)	831/1063 (78.2%)	832/1054 (78.9%)
ACE inhibitor/ARB	3749/4271 (87.8%)	942/1077 (87.5%)	937/1077 (87.0%)	948/1063 (89.2%)	922/1054 (87.5%)
Vitamin K antagonist/DOAC	303/4318 (7.0%)	47/1080 (4.4%)	63/1082 (5.8%)	83/1076 (7.7%)	110/1080 (10.2%)
Diuretic	997/4272 (23.3%)	155/1077 (14.4%)	227/1077 (21.1%)	239/1063 (22.5%)	376/1055 (35.6%)
Insulin	277/4270 (6.5%)	45/1076 (4.2%)	48/1077 (4.5%)	69/1063 (6.5%)	115/1054 (10.9%)
Oral antidiabetics	503/4270 (11.8%)	97/1077 (9.0%)	96/1077 (8.9%)	129/1062 (12.1%)	181/1054 (17.2%)

Immunosuppressive drugs	112/4272 (2.6%)	19/1077 (1.8%)	20/1077 (1.9%)	20/1063 (1.9%)	53/1055 (5.0%)
PPI	1258/4272 (29.4%)	241/1077 (22.4%)	276/1077 (25.6%)	334/1063 (31.4%)	407/1055 (38.6%)
Outcomes§					
MACE	314/4318 (7.3%)	41/1080 (3.8%)	66/1082 (6.1%)	77/1076 (7.2%)	130/1080 (12.0%)
Mortality	148/4318 (3.4%)	13/1080 (1.2%)	21/1082 (1.9%)	33/1076 (3.1%)	81/1080 (7.5%)

Categorical data are shown as numbers and valid percentages (%). Continuous data are presented as median and interquartile range (IQR). ACE, angiotensin-converting enzyme; ACS, acute coronary syndrome; ARB, angiotensin II receptor blocker; CABG, coronary artery bypass grafting; CCS, chronic coronary syndrome; DOAC, direct oral anticoagulant; MACE, major adverse cardiovascular events; PCI, percutaneous coronary intervention; PPI, proton pump inhibitor. †Refers to vessels with significant coronary stenosis. §Refers to 1-year outcomes.

Table S3: Baseline characteristics of patients in case-cohort analysis of German ACS cohort

	**Subcohort (*n* = 139)**	**Subjects with incident MACE at 1 year (*n* = 117)**
Age (years)	68 (57 – 77)	77 (69 – 83)
Female	33/139 (23.7%)	40/117 (34.2%)
BMI (kg/m^2)	27.1 (25.0–30.5)	28.0 (24.7–30.5)
Heart rate (bpm)	71 (62 – 80)	82 (70 – 92)
Systolic blood pressure (mm Hg)	149 (133 – 164)	148 (127 – 164)
STEMI	34/139 (24.5%)	24/117 (20.5%)
Smoking	42/132 (31.8%)	38/108 (35.2%)
Medical history		
Diabetes	26/138 (18.8%)	43/112 (38.4%)
Prediabetes[#]	21/72 (29.2%)	8/41 (19.5%)
Hypertension	108/139 (77.7%)	97/113 (85.5%)
Hypercholesterolemia	92/135 (68.2%)	67/107 (62.6%)
Myocardial infarction	32/135 (23.7%)	38/110 (34.5%)
Family history of CAD	52/131 (39.7%)	20/102 (19.6%)
Prior PCI	51/135 (37.8%)	44/107 (41.1%)
Prior CABG	12/136 (8.8%)	21/109 (19.3%)
Cerebrovascular disease	..	..
Peripheral artery disease	..	..
Heart failure	17/131 (13.0%)	26/102 (25.5%)
Chronic liver disease	3/139 (2.2%)	3/117 (2.6%)
Dialysis	..	..
Clinical chemistry and haematology		
Leukocyte count (G/L)	7.6 (5.6–13.3)	7.9 (6.2–13.4)
hs-CRP (mg/L)	2.5 (2.0–10.6)	13.7 (2.9–54.0)
Total cholesterol (mg/dL)	189 (172–206)	188 (164–201)
LDL-C (mg/dL)	91 (79–106)	97 (84–109)
HDL-C (mg/dL)	47 (43–50)	44 (42–47)
Lipoprotein (a) (mg/dL)	..	..
Triglycerides (mg/dL)	148 (109–185)	147 (119–187)
HbA1c (%)	5.8 (5.4–6.2)	6.6 (5.8–7.4)
Glucose (mmol/L)	6.3 (5.4–7.4)	8.0 (6.0–9.5)
NT-proBNP (ng/L)	504 (153–2312)	2446.0 (832.0–7308.0)
hs-TnT (ng/L)	28 (7–182)	173.0 (45.0–955.0)
Creatinine (mg/dL)	0.9 (0.8–1.1)	1.1 (0.9–1.5)
eGFR (mL/min/1.73m^2)	88 (76–97)	72.9 (63.6–84.9)

ImP metabolism		
ImP (nM)	13.1 (9.3 – 20.0)	34.6 (17.8 – 59.9)
Urocanate (nM)	127.7 (81 – 203.1)	146.1 (98.9 – 242.2)
Histidine (µM)	87.9 (67.8 – 102.9)	77.3 (64.8 – 93.8)
Baseline medication		
Aspirin	33/139 (23.7%)	40/117 (34.2%)
P2Y12 inhibitor	..	..
Beta-blocker	22/139 (15.8%)	10/117 (8.6%)
ACE inhibitor/ARB	100/139 (71.9%)	80/117 (68.4%)
Vitamin K antagonist/DOAC	19/139 (13.7%)	16/117 (13.7%)
Diuretic	50/139 (36.0%)	41/117 (35.0%)
Insulin	35/139 (25.2%)	28/117 (23.9%)
Oral antidiabetics	4/139 (2.9%)	7/117 (6.0%)
Immunosuppressive drugs	..	..
PPI	32/139 (23.0%)	29/117 (24.8%)

Note that the subcohort includes 6 subjects with incident MACE and 3 subjects with incident death (see Table S4). Categorical data are shown as numbers and valid percentages (%). Continuous data are presented as median and interquartile range (IQR). ACE, angiotensin-converting enzyme; ACS, acute coronary syndrome; ARB, angiotensin II receptor blocker; BMI, body mass index; CABG, coronary artery bypass grafting; CAD, coronary artery disease; DOAC, direct oral anticoagulant; eGFR, estimated glomerular filtration rate; HDL-C, high-density lipoprotein cholesterol; hs-CRP, high-sensitivity C-reactive protein; hs-TnT, high-sensitivity troponin T; ImP, imidazole propionate; LDL-C, low-density lipoprotein cholesterol; MACE, major adverse cardiovascular events; NT-proBNP, N-terminal pro B-type natriuretic peptide; PCI, percutaneous coronary intervention; PPI, proton pump inhibitor; STEMI, ST-segment elevation myocardial infarction. #defined as HbA1c of 5.7–6.4% in individuals without a history of diabetes. Eight patients were excluded due to insufficient sample volume (see Suppl. figure 1).

Table S4: Treatment characteristics of patients in case-cohort analysis of German ACS cohort

	**Subcohort (*n* = 139)**	**Subjects with incident MACE at 1 year (*n* = 117)**
Revascularization strategy during baseline visit		
PCI	81/126 (64.3%)	74/101 (73.3%)
CABG	..	..
Symptom-to-door time (minutes)	..	..
No. of affected vessels†		
One	21/126 (16.7%)	11/101 (10.9%)
Two	29/126 (23.0%)	12/101 (11.9%)
Three	64/126 (50.8%)	76/101 (75.3%)
Left ventricular ejection fraction (%)		
> 50	47/134 (35.1%)	19/110 (17.3%)
50 – 40	43/134 (32.1%)	23/110 (20.9%)
40 – 30	31/134 (23.1%)	29/110 (26.4%)
< 30	13/134 (9.7%)	39/110 (35.4%)
Discharge medication		
Aspirin	125/138 (90.6%)	100/115 (87.0%)
P2Y12 inhibitor	102/139 (73.4%)	73/116 (62.9%)
Beta-blocker	124/139 (89.2%)	101/116 (87.1%)
ACE inhibitor/ARB	121/139 (87.1%)	102/116 (87.9%)
Vitamin K antagonist/DOAC	18/139 (12.9%)	16/116 (13.8%)
Diuretic	..	..
Insulin	14/139 (10.1%)	11/116 (9.5%)
Oral antidiabetics	22/139 (15.8%)	19/116 (16.4%)
Immunosuppressive drugs	..	..
PPI	40/139 (28.8%)	33/117 (28.2%)
Outcomes		
MACE at 1 year	6/139 (4.3%)	117/117 (100.0%)
Mortality at 1 year	3/139 (2.2%)	75/117 (64.1%)

Note that the subcohort includes 6 subjects with incident MACE and 3 subjects with incident death. Categorical data are shown as numbers and valid percentages (%). Continuous data are presented as median and interquartile range (IQR). ACE, angiotensin-converting enzyme; ACS, acute coronary syndrome; ARB, angiotensin II receptor blocker; CABG, coronary artery bypass graft; DOAC, direct oral anticoagulant; MACE, major adverse cardiovascular events; PCI, percutaneous coronary intervention; PPI, proton pump inhibitor. †Refers to vessels with significant coronary stenosis. In the subcohort and in subjects with incident MACE at 1 year, no significant coronary stenosis was defined in 12 patients and 2 patients, respectively. Eight patients were excluded due to insufficient sample volume (see Suppl. figure 1).

Table S5: Baseline characteristics of patients in case-cohort analysis of German ACS cohort stratified by imidazole propionate quartiles

	**All Patients in Subcohort (*n* = 139)**	**ImP Quartile 1 < 9.4 nM (*n* = 37)**	**ImP Quartile 2 9.4 – 14.3 nM (*n* = 44)**	**ImP Quartile 3 14.3 – 24.4 nM (*n* = 35)**	**ImP Quartile 4 > 24.4 nM (*n* = 23)**
Age (years)	68 (57–77)	59 (53–67)	69 (59–76)	72 (59–77)	77 (68–83)
Female	33/139 (23.7%)	12/37 (32.4%)	11/44 (25.0%)	4/35 (11.4%)	6/23 (26.1%)
BMI (kg/m²)	27.1 (25.0–30.5)	27.1 (25.1–29.6)	27.0 (25.1–31.7)	27.7 (24.6–29.4)	27.0 (23.7-31.9)
Body surface area (m²)	..	..	..	..	..
Heart rate (bpm)	71 (62–80)	73 (66–84)	71 (61–77)	70 (61–79)	72 (68–86)
Systolic blood pressure (mm Hg)	149 (133–164)	150 (135–167)	157 (140–172)	140 (121–152)	142 (115–152)
Signs and symptoms of heart failure (ACS: Killip class, CCS: NYHA class)					
I	134/139 (96.4%)	35/37 (94.6%)	44/44 (100%)	32/35 (91.4%)	23/23 (100%)
II	4/139 (2.9%)	2/37 (5.4%)	0/44 (0.0%)	2/35 (5.7%)	0/23 (0.0%)
III	0/139 (0.0%)	0/37 (0.0%)	0/44 (0.0%)	0/35 (0.0%)	0/23 (0.0%)
IV	1/139 (0.7%)	0/37 (0.0%)	0/44 (0.0%)	1/35 (2.9%)	0/23 (0.0%)
Smoking	42/132 (31.8%)	9/36 (25.0%)	14/42 (33.3%)	12/33 (36.4%)	7/21 (33.3%)
Medical history					
Diabetes	26/138 (18.8%)	3/37 (8.1%)	8/44 (18.2%)	7/34 (20.6%)	8/23 (34.8%)
Prediabetes[#]	21/72 (29.2%)	2/11 (18.2%)	10/31 (32.3%)	7/18 (38.9%)	2/12 (16.7%)
Hypertension	108/139 (77.7%)	25/37 (67.6%)	35/44 (79.6%)	27/35 (77.1%)	21/23 (91.3%)
Hyperlipoproteinemia	92/135 (68.2%)	21/36 (58.3%)	29/42 (69.1%)	25/34 (73.5%)	17/23 (73.9%)
Myocardial infarction	32/135 (23.7%)	7/36 (19.4%)	7/43 (16.3%)	11/34 (32.4%)	7/22 (31.8)
Family history of CAD	52/131 (39.7%)	17/36 (47.2%)	16/41 (39.0%)	14/34 (41.2%)	5/20 (25.0%)
Prior PCI	51/135 (37.8%)	12/37 (32.4%)	14/43 (32.6%)	14/33 (42.4%)	11/22 (50.0%)
Prior CABG	12/136 (8.8%)	4/37 (10.8%)	1/43 (2.3%)	2/34 (5.9%)	5/22 (22.7%)

Cerebrovascular disease	..	..	..	..	..
Peripheral artery disease	..	..	..	..	..
Heart failure	17/131 (13.0%)	3/36 (8.3%)	3/43 (7.0%)	6/33 (18.2%)	5/19 (26.3%)
Chronic liver disease	3/139 (2.2%)	0/37 (0.0%)	2/44 (4.6%)	0/35 (0%)	1/23 (4.4%)
Dialysis	..	..	..	..	..
Clinical chemistry and haematology					
Leukocyte count (G/L)	7.6 (5.6–13.3)	7.6 (5.8–9.5)	7.2 (5.6–12.4)	7.6 (5.6–14.0)	7.9 (6.5–16.3)
hs-CRP (mg/L)	2.5 (2.0–10.6)	2.0 (2.0–9.2)	2.0 (2.0–4.8)	2.2 (2.0–12.9)	10.8 (2.9–61.4)
Total cholesterol (mg/dL)	189 (172–206)	195 (165–211)	194 (181–205)	179 (169–196)	195 (181–213)
LDL-C (mg/dL)	91 (79–106)	91 (85–103)	85 (78–104)	92 (76–105)	95 (87–109)
HDL-C (mg/dL)	47 (43–50)	44 (40–50)	47 (43–48)	48 (44–54)	48 (45–50)
Lipoprotein (a) (mg/dL)	..	..	..	..	..
Triglycerides (mg/dL)	148 (109–185)	150 (138–185)	147 (103–186)	141 (105–179)	159 (121–188)
HbA1c (%)	5.8 (5.4–6.2)	5.6 (5.4–5.9)	5.8 (5.4–6.2)	5.8 (5.3–6.2)	6.1 (5.6–6.8)
Glucose (mmol/L)	6.3 (5.4–7.4)	5.9 {5.3–6.9)	5.9 (5.2–6.8)	7.0 (6.0–8.7)	6.5 (5.4–7.2)
NT-proBNP (ng/L)	504 (153–2312)	182 (96–638)	384 (135–779)	2260 (343–7672)	3225 (498–6194)
hs-TnT (ng/L)	28 (7–182)	18 (6–237)	18 (4–94)	50 (11–193)	70 (19–245)
Creatinine (mg/dL)	0.9 (0.8–1.1)	0.8 (0.7–0.9)	0.9 (0.7–1.0)	1.0 (0.8–1.3)	1.3 (1.0–2.2)
eGFR (mL/min/1.73m^2)	88 (76–97)	96 (88–101)	89 (80–98)	80 (72–89)	67 (60–88)
ImP metabolism					
ImP (nM)	13 (9–20)	8 (7–9)	12 (10–13)	17 (16–21)	36 (29–94)
Urocanate (nM)	128 (81–204)	128 (69–204)	148 (94–228)	122 (81–164)	157 (75–164)
Histidine (µM)	88 (68–103)	81 (70–105)	92 (75–109)	81 (67–98)	78 (64–102)
Baseline medication					
Aspirin	33/139 (23.7%)	5/37 (13.5%)	14/44 (31.8%)	8/35 (22.9%)	6/23 (26.1%)
P2Y12 inhibitor	..	..	..	..	..

Beta-blocker	22/139 (15.8%)	7/37 (18.9%)	4/44 (9.1%)	7/35 (20.0%)	4/23 (17.4%)
ACE inhibitor/ARB	100/139 (71.9%)	25/37 (67.6%)	29/44 (65.9%)	28/35 (80.0%)	18/23 (78.3%)
Vitamin K antagonist/DOAC	19/139 (13.7%)	7/37 (18.9%)	4/44 (9.1%)	5/35 (14.3%)	3/23 (13.0%)
Diuretic	50/139 (36.0%)	9/37 (24.3%)	17/44 (38.6%)	14/35 (40.0%)	10/23 (43.5%)
Insulin	35/139 (25.2%)	4/37 (10.8%)	13/44 (29.6%)	11/35 (31.4%)	7/23 (30.4%)
Oral antidiabetics	4/139 (2.9%)	0/37 (0.0%)	1/44 (2.3%)	1/35 (2.9%)	2/23 (8.7%)
Immunosuppressive drugs	..	..	..	..	..
PPI	32/139 (23.0%)	8/37 (21.6%)	11/44 (25.0%)	10/35 (28.6%)	3/23 (13.0%)

Categorical data are shown as numbers and valid percentages (%). Continuous data are presented as median and interquartile range (IQR). ACE, angiotensin-converting enzyme; ACS, acute coronary syndrome; ARB, angiotensin II receptor blocker; BMI, body mass index; CABG, coronary artery bypass grafting; CAD, coronary artery disease; CCS, chronic coronary syndrome; DOAC, direct oral anticoagulant; eGFR, estimated glomerular filtration rate; HbA1c, hemoglobin A1c; HDL-C, high-density lipoprotein cholesterol; hs-CRP, high-sensitivity C-reactive protein; hs-TnT, high-sensitivity troponin T; ImP, imidazole propionate; LDL-C, low-density lipoprotein cholesterol; NT-proBNP, N-terminal pro B-type natriuretic peptide; NYHA, New York Heart Association; PCI, percutaneous coronary intervention; PPI, proton pump inhibitor. #defined as HbA1c of 5.7–6.4% in individuals without a history of diabetes.

Table S6: Treatment characteristics of patients in case-cohort analysis of German ACS cohort stratified by imidazole propionate quartiles

	**All Patients in Subcohort (*n* = 139)**	**ImP Quartile 1 < 9.4 nM (*n* = 37)**	**ImP Quartile 2 9.4 – 14.3 nM (*n* = 44)**	**ImP Quartile 3 14.3 – 24.4 nM (*n* = 35)**	**ImP Quartile 4 > 24.4 nM (*n* = 23)**
Revascularization strategy during baseline visit					
PCI	81/126 (63.3%)	19/34 (55.9%)	23/39 (59.0%)	22/33 (66.7%)	17/20 (85.0%)
CABG	..	..	..	..	..
Symptom-to-door time (minutes)	..	..	..	..	..
No. of affected vessels†					
One	21/126 (16.7%)	6/34 (17.7%)	5/39 (12.8%)	6/33 (18.2%)	4/20 (20.0%)
Two	29/126 (23.0%)	12/34 (35.3%)	11/39 (28.1%)	3/33 (9.1%)	3/20 (15.0%)
Three	64/126 (50.8%)	12/34 (35.3%)	18/39 (46.2%)	22/33 (66.7%)	12/20 (60.0%)
Left ventricular ejection fraction (%)					
> 50	47/134 (35.1%)	10/34 (29.4%)	19/44 (43.2%)	11/35 (31.4%)	7/21 (33.3%)
50–40	43/134 (32.1%)	13/34 (38.3%)	15/44 (34.1%)	10/35 (28.6%)	5/21 (23.8%)
40–30	31/134 (23.1%)	10/34 (29.4%)	8/44 (18.2%)	9/35 (25.7%)	4/21 (19.1%)
< 30	13/134 (9.7%)	1/34 (2.9%)	2/44 (4.5%)	5/35 (14.3%)	5/21 (23.8%)
Discharge medication					
Aspirin	125/138 (90.6%)	32/37 (86.5%)	39/43 (90.7%)	32/35 (91.4%)	22/23 (96.7%)
P2Y12 inhibitor	102/139 (73.4%)	27/37 (73.0%)	30/44 (68.2%)	27/35 (771.%)	18/23 (78.3%)
Beta-blocker	124/139 (89.2%)	32/37 (86.5%)	40/44 (90.9%)	31/35 (88.6%)	21/23 (91.3%)
ACE inhibitor/ARB	121/139 (87.1%)	31/37 (83.3%)	38/44 (86.4%)	32/35 (91.4%)	20/23 (87.0%)
Vitamin K antagonist/DOAC	18/139 (13.0%)	2/37 (5.4%)	8/44 (18.2%)	7/35 (20.0%)	1/23 (4.4%)
Diuretic	..	..	..	..	..
Insulin	14/139 /10.1%)	6/37 (16.2%)	5/44 (11.4%)	2/35 (5.7%)	1/23 (4.4%)
Oral antidiabetics	22/139 (15.8%)	9/37 (24.3%)	8/44 (18.2%)	1/35 (2.9%)	4/23 (17.4%)

Immunosuppressive drugs	..	..	..	..	..
PPI	40/139 (28.8%)	9/37 (24.3%)	12/44 (27.3%)	13/35 (37.1%)	6/23 (26.1%)
Outcomes§					
MACE	6/139 (4.3%)	1/37 (2.7%)	0/44 (0.0%)	3/35 (8.6%)	2/23 (8.7%)
Mortality	3/139 (2.2%)	0/37 (0.0%)	0/44 (0.0%)	2/35 (5.7%)	1/23 (4.4%)

Categorical data are shown as numbers and valid percentages (%). Continuous data are presented as median and interquartile range (IQR). ACE, angiotensin-converting enzyme; ACS, acute coronary syndrome; ARB, angiotensin II receptor blocker; CABG, coronary artery bypass grafting; CCS, chronic coronary syndrome; DOAC, direct oral anticoagulant; ImP, imidazole propionate; MACE, major adverse cardiovascular events; PCI, percutaneous coronary intervention; PPI, proton pump inhibitor. †Refers to vessels with significant coronary stenosis. In the subcohort, no significant coronary stenosis was defined in 12 patients. §Refers to 1-year outcomes.

Table S7: Baseline characteristics of patients in Swiss CMR cohort

	**All patients** (***n*** **= 60)**	**ImP Quartile 1–3 0–24.4 nM** (***n*** **= 42)**	**ImP Quartile 4 > 24.4 nM** (***n*** **= 18)**
Age (years)	59.9 (54.6 – 68.6)	58.9 (53.1 – 67.5)	64.0 (58.0 – 71.6)
Female	7/60 (11.7%)	4/42 (9.5%)	3/18 (16.7%)
BMI (kg/m^2)	26.2 (24.6 – 30.0)	26.2 (24.9 – 29.8)	25.5 (24.3 – 30.0)
Heart rate (bpm)	70 (65 – 81)	70 (65 – 76)	77 (66 – 90)
Systolic blood pressure (mm Hg)	114 (107 – 127)	113 (104 – 123)	117 (110 – 127)
Smoking	30/60 (50.0%)	24/42 (57.1%)	6/18 (33.3%)
Medical history			
Diabetes	4/60 (6.7%)	2/42 (4.8%)	2/18 (11.1%)
Hypertension	22/60 (36.7%)	15/42 (35.7%)	7/18 (38.9%)
Hypercholesterolaemia	28/60 (46.7%)	19/42 (45.2%)	9/18 (50.0%)
Myocardial infarction	0/60 (0.0%)	0/42 (0.0%)	0/18 (0.0%)
Family history of CAD	14/60 (23.3%)	10/42 (23.8%)	4/18 (22.2%)
Prior PCI	1/60 (1.7%)	0/42 (0.0%)	1/18 (5.6%)
Prior CABG	1/60 (1.7%)	1/42 (2.4%)	0/18 (0.0%)
Cerebrovascular disease	1/60 (1.7%)	1/42 (2.4%)	0/18 (0.0%)
Peripheral artery disease	0/60 (0.0%)	0/42 (0.0%)	0/18 (0.0%)
Heart failure	0/60 (0.0%)	0/42 (0.0%)	0/18 (0.0%)
Chronic liver disease	1/60 (1.7%)	0/42 (0.0%)	1/18 (5.6%)
Dialysis	0/60 (0.0%)	0/42 (0.0%)	0/18 (0.0%)
Clinical chemistry and haematology			
Leukocyte count (G/L)	9.6 (7.9 – 13.3)	9.8 (7.9 – 13.2)	8.9 (8.0 – 13.0)
Total cholesterol (mg/dL)	174.0 (154.7 – 193.4)	177.1 (162.4 – 197.2)	162.4 (150.8 – 181.7)
LDL-C (mg/dL)	108.3 (85.1 – 135.3)	116.0 (92.8 – 139.2)	92.8 (84.1 – 109.2)
HDL-C (mg/dL)	44.1 (38.0 – 53.8)	45.6 (38.3 – 51.4)	41.8 (37.5 – 58.0)
Triglycerides (mg/dL)	115.6 (89.2 – 150.3)	116.0 (93.0 – 149.7)	108.9 (73.5 – 170.9)
Glucose (mmol/L)	6.1 (5.5 – 8.1)	6.0 (5.5 – 7.4)	7.7 (5.4 – 8.4)
NT-proBNP (ng/L)	914 (482 – 2071)	1078 (442 – 1855)	809 (677 – 2435)
hs-TnT (ng/L)	5112.0 (1679 – 367500)	5014 (1628 – 496750)	6446 (2709 – 9310)
Creatinine (mg/dL)	0.9 (0.8 – 1.0)	0.9 (0.8 – 1.0)	1.0 (0.9 – 1.2)
eGFR (mL/min/1.73m^2)	87.3 (76.8 – 100.6)	90.6 (80.2 – 104.3)	77.8 (64.8 – 96.2)
ImP metabolism			
ImP (nM)	10.0 (8.4 – 18.2)	9.1 (8.0 – 10.8)	24.1 (19.9 – 37.4)
Urocanate (nM)	125.5 (88.5 – 165.9)	127.4 (88.5 – 171.7)	122.7 (89.4 – 149.0)
Histidine (µM)	66.7 (60.1 – 76.1)	65.2 (60.5 – 75.6)	73.0 (57.3 – 76.2)
Baseline medication			

Aspirin	55/58 (94.8%)	38/40 (95.0%)	17/18 (94.4%)
P2Y12 inhibitor	55/58 (94.8%)	38/40 (95.0%)	17/18 (94.4%)
Beta-blocker	40/58 (69.0%)	29/40 (72.5%)	11/18 (61.1%)
ACE inhibitor/ARB	47/58 (81.0%)	33/40 (82.5%)	14/18 (77.8%)
Vitamin K antagonist/DOAC	7/58 (12.1%)	5/40 (12.5%)	2/18 (11.1%)
Diuretic	14/58 (24.1%)	6/40 (15.0%)	8/18 (44.4%)
Insulin	1/58 (1.7%)	0/40 (0.0%)	1/18 (5.6%)
Oral antidiabetics	3/58 (5.2%)	2/40 (5.0%)	1/18 (5.6%)
Immunosuppressive drugs	0/58 (0.0%)	0/40 (0.0%)	0/18 (0.0%)
PPI	36/58 (62.1%)	25/40 (62.5%)	11/18 (61.1%)

Categorical data are shown as numbers and valid percentages (%). Continuous data are presented as median and interquartile range (IQR). ACE, angiotensin-converting enzyme; ARB, angiotensin II receptor blocker; BMI, body mass index; CABG, coronary artery bypass grafting; CAD, coronary artery disease; CMR, cardiac magnetic resonance imaging; DOAC, direct oral anticoagulant; eGFR, estimated glomerular filtration rate; HDL-C, high-density lipoprotein cholesterol; hs-TnT, high-sensitivity troponin T; ImP, imidazole propionate; LDL-C, low-density lipoprotein cholesterol; NT-proBNP, N-terminal pro B-type natriuretic peptide; PCI, percutaneous coronary intervention; PPI, proton pump inhibitor.

Table S8: Treatment characteristics of patients in Swiss CMR cohort

	**All patients (*n* = 60)**	**ImP Quartile 1–3 0–24.4 nM (*n* = 42)**	**ImP Quartile 4 > 24.4 nM (*n* = 18)**
Revascularization strategy during baseline visit			
PCI	60/60 (100.0%)	42/42 (100.0%)	18/18 (100.0%)
Left ventricular ejection fraction (%)			
> 50	20/59 (33.9%)	12/41 (29.3%)	8/18 (44.4%)
50 – 40	25/59 (42.4%)	20/41 (48.8%)	5/18 (27.8%)
40 – 30	11/59 (18.6%)	7/41 (17.1%)	4/18 (22.2%)
< 30	3/59 (5.1%)	2/41 (4.9%)	1/18 (5.6%)
Discharge medication			
Aspirin	59/60 (98.3%)	42/42 (100.0%)	17/18 (94.4%)
P2Y12 inhibitor	60/60 (100.0%)	42/42 (100.0%)	18/18 (100.0%)
Beta-blocker	53/60 (88.3%)	37/42 (88.1%)	16/18 (88.9%)
ACE inhibitor/ARB	57/60 (95.0%)	40/42 (95.2%)	17/18 (94.4%)
Vitamin K antagonist/DOAC	10/60 (16.7%)	7/42 (16.7%)	3/18 (16.7%)
Diuretic	15/60 (25.0%)	9/42 (21.4%)	6/18 (33.3%)
Insulin	1/60 (1.7%)	0/42 (0.0%)	1/18 (5.6%)
Oral antidiabetics	3/60 (5.0%)	2/42 (4.8%)	1/18 (5.6%)
Immunosuppressive drugs	0/60 (0.0%)	0/42 (0.0%)	0/18 (0.0%)
PPI	45/60 (75.0%)	30/42 (71.4%)	15/18 (83.3%)

Categorical data are shown as numbers and valid percentages (%). Continuous data are presented as median and interquartile range (IQR). ACE, angiotensin-converting enzyme; ARB, angiotensin II receptor blocker; CMR, cardiac magnetic resonance imaging; DOAC, direct oral anticoagulant; ImP, imidazole propionate; PCI, percutaneous coronary intervention; PPI, proton pump inhibitor.

Table S9: Baseline characteristics of patients with CCS (German CCS cohort) stratified by imidazole propionate quartiles

	**All Patients (*n* = 577)**	**ImP Quartile 1 < 9.4 nM (*n* = 187)**	**ImP Quartile 2 9.4 – 14.3 nM (*n* = 126)**	**ImP Quartile 3 14.3 – 24.4 nM (*n* = 104)**	**ImP Quartile 4 > 24.4 nM (*n* = 160)**
Age (years)	76 (67–81)	71 (64–78)	75 (66–79)	78 (70–83)	80 (72–84)
Female	124/577 (21.5%)	57/187 (30.5%)	20/126 (15.9%)	21/104 (20.2%)	26/160 (16.2%)
BMI (kg/m²)	26.9 (24.5–30.0)	27.4 (24.7–30.2)	26.5 (24.6–29.8)	26.7 (24.5–30.1)	26.9 (24.4–29.9)
Body surface area (m²)	2.0 (1.8–2.1)	2.0 (1.9–2.1)	2.0 (1.9–2.1)	2.0 (1.8–2.1)	2.0 (1.8–2.1)
Heart rate (bpm)	..	..	..	..	..
Systolic blood pressure (mm Hg)	134 (121–149)	138 (126–152)	134 (122–146)	130 (120–146)	132 (119–149)
Signs and symptoms of heart failure (ACS: Killip class, CCS: NYHA class)					
I	93/555 (16.8%)	47/172 (27.3%)	25/122 (20.5%)	14/102(13.7%)	7/159 (4.4%)
II	294/555 (53.0%)	99/172 (57.6%)	64/122 (52.5%)	60/102 (58.8%)	71/159 (44.7%)
III	166/555 (29.9%)	26/172 (15.1%)	33/122 (27.0%)	27/102 (26.5%)	80/159 (50.3%)
IV	2/555 (0.4%)	0/172 (0.0%)	0/122 (0.0%)	1/102 (1.0%)	1/159 (0.6%)
Smoking	98/577 (17.0%)	33/187 (17.6%)	23/126 (18.3%)	20/104 (19.2%)	22/160 (13.8%)
Medical history					
Diabetes	178/577 (30.8%)	36/187 (19.3%)	30/126 (23.8%)	41/104 (39.4%)	71/160 (44.4%)
Prediabetes[#]	168/577 (29.1%)	59/187 (31.6%)	42/126 (33.3%)	26/104 (25.0%)	41/160 (25.6%)
Hypertension	477/577 (82.7%)	148/187 (79.1%)	102/126 (81.0%)	84/104 (80.8%)	143/160 (89.4%)
Hyperlipoproteinemia	378/577 (65.5%)	136/187 (72.7%)	79/126 (62.7%)	64/104 (61.5%)	99/160 (61.9%)
Myocardial infarction	405/576 (70.3%)	121/187 (64.7%)	88/125 (70.4%)	74/104 (71.2%)	122/160 (76.2%)
Family history of CAD	161/478 (33.7%)	65/152 (42.8%)	32/99 (32.3%)	29/93 (31.2%)	35/134 (26.1%)
Prior PCI	..	..	..	..	..
Prior CABG	62/577 (10.7%)	19/187 (10.2%)	7/126 (5.6%)	15/104 (14.4%)	21/160 (13.1%)

Cerebrovascular disease	74/577 (12.8%)	19/187 (10.2%)	11/126 (8.7%)	16/104 (15.4%)	28/160 (17.5%)
Peripheral artery disease	30/577 (5.2%)	6/187 (3.2%)	8/126 (6.3%)	6/104 (5.8%)	10/160 (6.2%)
Heart failure	..	..	..	..	..
Chronic liver disease	12/577 (2.1%)	1/187 (0.5%)	1/126 (0.8%)	2/104 (1.9%)	8/160 (5.0%)
Dialysis	..	..	..	..	..
Clinical chemistry and haematology					
Leukocyte count (G/L)	7.4 (6.0–8.9)	7.1 (5.8–8.6)	7.5 (6.0–9.2)	7.9 (6.5–9.4)	7.4 (6.0–8.8)
hs-CRP (mg/L)	2 (1–7)	1 (1–4)	2(1–5)	2 (1–5)	5 (1–11)
Total cholesterol (mg/dL)	153 (129–187)	157 (135–196)	152 (126–187)	149 (132–179)	150 (125–174)
LDL-C (mg/dL)	85 (65–115)	95 (71–130)	82 (65–115)	83 (62–116)	77 (61–103)
HDL-C (mg/dL)	46 (38–57)	48 (40–59)	47 (39–57)	47 (39–55)	42 (36–55)
Lipoprotein (a) (mg/dL)	8.3 (2.0–34.9)	8.3 (2.0–42.4)	7.0 (2.0–25.8)	6.3 (2.0–21.9)	11.4 (3.4–40.0)
Triglycerides (mg/dL)	121 (91–176)	128 (96–186)	117 (93–196)	119 (89–171)	119 (89–166)
HbA1c (%)	5.8 (5.5–6.3)	5.7 (5.4–6.0)	5.8 (5.5–6.3)	5.8 (5.5–6.3)	6.0 (5.6–6.6)
Glucose (mmol/L)	5.9 (5.2–7.7)	5.8 (5.0–6.9)	6.1 (5.1–7.6)	5.9 (5.3–8.7)	6.4 (5.4–8.4)
NT-proBNP (ng/L)	427 (134–1238)	135 (52–350)	478 (184–960)	520 (206–1250)	925 (401–4973)
hs-TnT (ng/L)	15 (9–30)	8 (6–17)	12 (8–22)	22 (11–33)	26 (15–38)
Creatinine (mg/dL)	1.0 (0.8–1.2)	0.9 (0.8–1.1)	0.9 (0.8–1.1)	1.1 (0.9–1.3)	1.1 (0.9–1.3)
eGFR (mL/min/1.73m^2)	73 (59–90)	84 (65–93)	80 (65–94)	67 (53–85)	63 (54–79)
ImP metabolism					
ImP (nM)	13 (8–26)	7 (6–8)	12 (11–13)	18.5 (16–21)	43 (32–70)
Urocanate (nM)	47 (33–84)	43 (30–71)	45 (32–78)	54 (33–95)	52 (37–99)
Histidine (µM)	62 (53–70)	62 (53–70)	60 (53–66)	59.5 (54–69)	64 (54–73)
Baseline medication					
Aspirin	381/577 (66.0%)	127/187 (67.9%)	82/126 (65.1%)	69/104 (66.3%)	103/160 (64.4%)
P2Y12 inhibitor	192/577 (33.3%)	66/187 (35.3%)	46/126 (36.5%)	36/104 (34.6%)	44/160 (27.5%)

Beta-blocker	371/577 (64.3%)	98/187 (52.4%)	81/126 (64.3%)	72/104 (69.2%)	120/160 (75.0%)
ACE inhibitor/ARB	447/577 (77.5%)	140/187 (74.9%)	93/126 (73.8%)	78/104 (75.0%)	136/160 (85.0%)
Vitamin K antagonist/DOAC	148/577 (25.6%)	30/187 (16.0%)	35/126 (27.8%)	25/104 (24.0%)	58/160 (36.2%)
Diuretic	247/577 (42.8%)	65/187 (34.8%)	46/126 (36.5%)	48/104 (46.2%)	88/160 (55.0%)
Insulin	57/577 (9.9%)	10/187 (5.3%)	7/126 (5.6%)	11/104 (10.6%)	29/160 (18.1%)
Oral antidiabetics	108/577 (18.7%)	21/187 (11.2%)	21/126 (16.7%)	22/104 (21.2%)	44/160 (27.5%)
Immunosuppressive drugs	..	..	..	..	..
PPI	247/577 (42.8%)	65/187 (34.8%)	61/126 (48.4%)	36/104 (34.6%)	85/160 (53.1%)

Categorical data are shown as numbers and valid percentages (%). Continuous data are presented as median and interquartile range (IQR). ACE, angiotensin-converting enzyme; ACS, acute coronary syndrome; ARB, angiotensin II receptor blocker; BMI, body mass index; CABG, coronary artery bypass grafting; CAD, coronary artery disease; CCS, chronic coronary syndrome; DOAC, direct oral anticoagulant; eGFR, estimated glomerular filtration rate; HbA1c, hemoglobin A1c; HDL-C, high-density lipoprotein cholesterol; hs-CRP, high-sensitivity C-reactive protein; hs-TnT, high-sensitivity troponin T; ImP, imidazole propionate; LDL-C, low-density lipoprotein cholesterol; NT-proBNP, N-terminal pro B-type natriuretic peptide; NYHA, New York Heart Association; PCI, percutaneous coronary intervention; PPI, proton pump inhibitor. *Values refer to the subcohort #defined as HbA1c of 5.7–6.4% in individuals without a history of diabetes.

Table S10: Treatment characteristics of patients with CCS (German CCS cohort) stratified by imidazole propionate quartiles

	**All Patients (*n* = 577)**	**ImP Quartile 1 < 9.4 nM (*n* = 187)**	**ImP Quartile 2 9.4 – 14.3 nM (*n* = 126)**	**ImP Quartile 3 14.3 – 24.4 nM (*n* = 104)**	**ImP Quartile 4 > 24.4 nM (*n* = 160)**
Revascularization strategy during baseline visit					
PCI	350/577 (60.7%)	125/187 (66.8%)	81/126 (64.3%)	55/104 (52.9%)	89/160 (55.6%)
CABG	..	..	..	..	..
Symptom-to-door time (minutes)	..	..	..	..	..
No. of affected vessels†					
One	135/577 (23.4%)	45/187 (24.1%)	29/126 (23.0%)	28/104 (26.9%)	33/160 (20.6%)
Two	194/577 (33.6%)	73/187 (39.0%)	47/126 (37.3%)	24/104 (23.1%)	50/160 (31.2%)
Three	248/577 (43.0%)	69/187 (36.9%)	50/126 (39.7%)	52/104 (50.0%)	77/160 (48.1%)
Left ventricular ejection fraction (%)					
> 50	437/577 (75.7%)	166/187 (88.8%)	93/126 (73.8%)	81/104 (77.9%)	97/160 (60.6%)
50–40	81/577 (14.0%)	19/187 (10.2%)	15/126 (11.9%)	16/104 (15.4%)	31/160 (19.4%)
40–30	38/577 (6.6%)	2/187 (1.1%)	14/126 (11.1%)	4/104 (3.8%)	18/160 (11.2%)
< 30	21/577 (3.6%)	0/187 (0.0%)	4/126 (3.2%)	3/104 (2.9%)	14/160 (8.8%)
Discharge medication					
Aspirin	..	..	..	..	..
P2Y12 inhibitor	..	..	..	..	..
Beta-blocker	..	..	..	..	..
ACE inhibitor/ARB	..	..	..	..	..
Vitamin K antagonist/DOAC	..	..	..	..	..
Diuretic	..	..	..	..	..
Insulin	..	..	..	..	..
Oral antidiabetics	..	..	..	..	..

Immunosuppressive drugs	..	..	..	..	..
PPI	..	..	..	..	..
Outcomes§					
MACE	164/577 (28.42%)	30/187 (16.0%)	26/126 (20.6%)	34/104 (32.7%)	74/160 (46.2%)
Mortality	47/577 (8.15%)	7/187 (3.7%)	5/126 (4.0%)	12/104 (11.5%)	23/160 (14.4%)

Categorical data are shown as numbers and valid percentages (%). Continuous data are presented as median and interquartile range (IQR). ACE, angiotensin-converting enzyme; ACS, acute coronary syndrome; ARB, angiotensin II receptor blocker; CABG, coronary artery bypass grafting; CCS, chronic coronary syndrome; DOAC, direct oral anticoagulant; ImP, imidazole propionate; MACE, major adverse cardiovascular events; PCI, percutaneous coronary intervention; PPI, proton pump inhibitor. †Refers to vessels with significant coronary stenosis. §Refers to 3-year outcomes.

Table S11: Baseline characteristics of patients with ACS (Swiss ACS cohort and German ACS cohort) and CCS (German CCS cohort) in fully adjusted model

	Swiss ACS cohort	German ACS cohort		German CCS cohort
	***n* = 3966**	**Subcohort (*n* = 137)**	**Subjects with incident MACE at 1 year (*n* = 112)**	***n* = 385**
Age (years)	63 (54–72)	68 (57–77)	78 (70–83)	76 (67–82)
Female	801/3966 (20.2%)	33/137 (24.1%)	38/112 (66.1%)	84/385 (21.8%)
BMI (kg/m²)	26.6 (24.2–29.4)	27.1 (24.8–30.6)	27.8 (24.6–30.0)	27.0 (24.4–30.1)
Body surface area (m²)	1.9 (1.8–2.1)	..	..	2.0 (1.8–2.1)
Heart rate (bpm)	75 (66–86)	70 (62–80)	82 (70–92)	..
Systolic blood pressure (mm Hg)	128 (113–143)	149 (133–164)	149 (127–165)	134 (120–148)
Signs and symptoms of heart failure (ACS: Killip class, CCS: NYHA class)				
I	3371/3703 (87.4%)	132/137 (65.4%)	99/112 (88.4%)	61/370 (16.5%)
II	332/3703 (8.6%)	4/137 (2.9%)	9/112 (8.0%)	196/370 (53.0%)
III	78/3703 (2.0%)	0/137 (0.0%)	2/112 (1.8%)	111/370 (30.0%)
IV	75/3703 (1.9%)	1/137 (0.7%)	2/112 (1.8%)	2/370 (0.5%)
Smoking	1511/3915 (38.6%)	41/130 (31.5%)	38/103 (36.9%)	61/385 (15.8%)
Medical history				
Diabetes	686/3966 (17.3%)	26/137 (19.0%)	43/112 (38.4%)	119/385 (30.9%)
Prediabetes#	571/1985 (28.8%)	21/72 (29.2%)	8/41 (19.5%)	112/385 (29.1%)
Hypertension	2212/3964 (55.8%)	107/137 (78.1%)	96/112 (85.7%)	311/385 (80.8%)
Hyperlipoproteinemia	2536/3966 (63.9%)	92/133 (69.2%)	66/106 (62.3%)	255/385 (66.2%)
Myocardial infarction	481/3958 (12.2%)	32/133 (24.1%)	37/108 (34.3%)	266/385 (69.1%)
Family history of CAD	975/3928 (24.8%)	52/129 (40.3%)	20/102 (19.6%)	121/335 (36.1%)
Prior PCI	590/3965 (14.9%)	51/133 (38.4%)	43/105 (41.0%)	..
Prior CABG	158/3966 (4.0%)	12/134 (9.0%)	20/107 (18.7%)	48/385 (12.5%)

Cerebrovascular disease	144/3966 (3.6%)	..	..	54/385 (14.0%)
Peripheral artery disease	226/3966 (5.7%)	..	..	24/385 (6.2%)
Heart failure	53/3964 (1.3%)	17/129 (13.2%)	26/100 (26.0%)	..
Chronic liver disease	24/3964 (0.6%)	3/137 (2.2%)	3/112 (2.7%)	9/385 (2.3%)
Dialysis	19/3966 (0.5%)	..	..	..
Clinical chemistry and haematology				
Leukocyte count (G/L)	9.6 (7.4–12.1)	7.6 (5.6–13.2)	7.9 (6.3–13.4)	7.5 (6.0–8.9)
hs-CRP (mg/L)	3 (1–7)	2.4 (2.0–10.5)	13.6 (2.9–52.8)	2 (1–7)
Total cholesterol (mg/dL)	190 (159–219)	189 (172–206)	188 (164–202)	153 (129–186)
LDL-C (mg/dL)	120 (92–149)	91 (87–109)	95 (83–103)	86 (64–115)
HDL-C (mg/dL)	44 (36–53)	47 (43–50)	44 (42–47)	46 (38–57)
Lipoprotein (a) (mg/dL)	6.7 (2.5–22.1)	..	..	9.4 (2.0–31.7)
Triglycerides (mg/dL)	89 (60–136)	149 (109–185)	148 (119–187)	120 (90–172)
HbA1c (%)	5.8 (5.5–6.3)	5.8 (5.4–6.2)	6.6 (5.8–7.4)	5.8 (5.5–6.3)
Glucose (mmol/L)	6.4 (5.6–7.9)	6.3 (5.4–7.2)	8.0 (6.0–9.5)	6.1 (5.1–7.8)
NT-proBNP (ng/L)	334 (107–1161)	504 (153–2312)	2446 (832–7308)	451 (134–1281)
hs-TnT (ng/L)	197 (58–649)	27 (7–173)	167 (42–903)	15 (9–30)
Creatinine (mg/dL)	0.9 (0.7–1.0)	0.9 (0.8–1.1)	1.1 (0.9–1.5)	1.0 (0.8–1.2)
eGFR (mL/min/1.73m^2)	93 (77–103)	87 (76–97)	73 (64–85)	72 (59–91)
ImP metabolism				
ImP (nM)	14 (9–24)	13 (9.2–19.8)	30 (17–60)	14 (9–30)
Urocanate (nM)	44 (30–66)	128 (81–204)	148 (101–242)	46 (31–75)
Histidine (µM)	66 (57–76)	88 (68–103)	78 (66–94)	62 (53–71)
Baseline medication				
Aspirin	1121/2548 (44.0%)	32/137 (23.4%)	38/112 (33.9%)	246/385 (63.9%)
P2Y12 inhibitor	299/1797 (16.6%)	..	..	112/385 (29.1%)

Beta-blocker	891/2548 (35.0%)	22/137 (16.1%)	10/112 (8.9%)	241/385 (62.6%)
ACE inhibitor/ARB	1345/2531 (53.1%)	98/137 (71.5%)	78/112 (69.6%)	288/385 (74.8%)
Vitamin K antagonist/DOAC	159/2547 (6.2%)	19/137 (13.9%)	14/112 (12.5%)	107/385 (27.8%)
Diuretic	616/2544 (24.2%)	49/137 (35.8%)	37/112 (33.0%)	178/385 (46.2%)
Insulin	198/2547 (7.8%)	35/137 (25.6%)	28/112 (25.0%)	38/385 (9.9%)
Oral antidiabetics	453/2547 (17.8%)	4/137 (2.9%)	6/112 (5.4%)	67/385 (17.4%)
Immunosuppressive drugs	105/2547 (4.1%)	..	..	..
PPI	595/2547 (23.4%)	32/137 (23.4%)	28/112 (25.0%)	164/385 (42.6%)

Categorical data are shown as numbers and valid percentages (%). Continuous data are presented as median and interquartile range (IQR). Data are shown for patients included in the fully adjusted Cox-analyses (complete cases). ACE, angiotensin-converting enzyme; ACS, acute coronary syndrome; ARB, angiotensin II receptor blocker; BMI, body mass index; CABG, coronary artery bypass grafting; CAD, coronary artery disease; CCS, chronic coronary syndrome; DOAC, direct oral anticoagulant; eGFR, estimated glomerular filtration rate; HbA1c, hemoglobin A1c; HDL-C, high-density lipoprotein cholesterol; hs-CRP, high-sensitivity C-reactive protein; hs-TnT, high-sensitivity troponin T; ImP, imidazole propionate; LDL-C, low-density lipoprotein cholesterol; NT-proBNP, N-terminal pro B-type natriuretic peptide; NYHA, New York Heart Association; PCI, percutaneous coronary intervention; PPI, proton pump inhibitor. #defined as HbA1c of 5.7–6.4% in individuals without a history of diabetes.

Table S12: Treatment characteristics of patients with ACS (Swiss ACS cohort and German ACS cohort) and CCS (German CCS cohort) in fully adjusted model

	Swiss ACS cohort	German ACS cohort		German CCS cohort
	***n* = 3966**	**Subcohort (*n* = 137)**	**Subjects with incident MACE at 1 year (*n* = 112)**	***n* = 385**
Revascularization strategy during baseline visit				
PCI	3625/3966 (91.4%)	79/124 (63.7%)	70/96 (72.9%)	227/385 (59.0%)
CABG	116/3966 (2.9%)	..	..	
Symptom-to-door time (minutes)	180 (105–420)	..	..	..
No. of affected vessels†				
One	3086/3755 (82.2%)	20/124 (16.1%)	11/96 (11.5%)	97/385 (25.2%)
Two	520/3755 (13.8%)	29/124 (23.4%)	10/96 (10.4%)	124/385 (32.2%)
Three	149/3755 (4.0%)	63/124 (50.8%)	73/96 (76.0%)	164/385 (42.6%)
Left ventricular ejection fraction (%)				
> 50	1248/2474 (50.4%)	46/132 (34.9%)	10/105 (18.1%)	19/385 (4.9%)
50–40	778/2474 (31.4%)	43/132 (32.6%)	23/105 (21.9%)	26/385 (6.8%)
40–30	287/2474 (11.6%)	30/132 (22.7%)	26/105 (24.8%)	45/385 (11.7%)
< 30	161/2474 (6.5%)	13/132 (9.9%)	37/105 (35.2%)	295/385 (76.6%)
Discharge medication				
Aspirin	3897/3932 (99.1%)	123/136 (90.4%)	96/110 (87.3%)	..
P2Y12 inhibitor	3744/3831 (97.7%)	100/137 (73.0%)	69/111 (62.2%)	..
Beta-blocker	3089/3929 (78.6%)	122/137 (89.1%)	97/111 (87.4%)	..
ACE inhibitor/ARB	3449/3931 (87.7%)	120/137 (87.6%)	97/111 (87.4%)	..
Vitamin K antagonist/DOAC	281/3966 (7.1%)	18/137 (13.1%)	16/111 (14.4%)	..
Diuretic	912/3932 (23.2%)	..	..	..
Insulin	257/3932 (6.5%)	13/137 (9.5%)	10/111 (9.0%)	..

Oral antidiabetics	459/3931 (11.7%)	21/137 (15.3%)	19/111 (17.1%)	..
Immunosuppressive drugs	99/3932 (2.5%)	..	..	..
PPI	1158/3932 (29.5%)	40/137 (29.2%)	31/112 (27.7%)	..
Outcomes§				
MACE	288/3966 (7.3%)	6/137 (4.4%)	112/112 (100%)	113/385 (29.4%)
Mortality	128/3966 (3.2%)	3/137 (2.2%)	71/112 (63.4%)	33/385 (8.6%)

Categorical data are shown as numbers and valid percentages (%). Continuous data are presented as median and interquartile range (IQR). Data are shown for patients included in the fully adjusted Cox-analyses (complete cases). ACE, angiotensin-converting enzyme; ACS, acute coronary syndrome; ARB, angiotensin II receptor blocker; CABG, coronary artery bypass grafting; CCS, chronic coronary syndrome; DOAC, direct oral anticoagulant; MACE, major adverse cardiovascular events; PCI, percutaneous coronary intervention; PPI, proton pump inhibitor.
§Refers to 1-year outcomes for ACS and to 3-year outcomes for CCS included in the analyses.

Table S13: Correlation of imidazole propionate, histidine, and urocanate levels with baseline characteristics in patients with CAD (pooled data from Swiss ACS cohort and German CCS cohort)

ImP	Variable	No. of patients	Partial correlation coefficient	*P-value*	Symbol
Unadjusted	LVEF	3302	-0.110	< 0.001	***
	HbA1c	2426	0.196	< 0.001	***
	Glucose	4456	0.100	< 0.001	***
	BMI	4791	0.043	0.003	**
	hs-CRP	4452	0.172	< 0.001	***
	Leukocyte count	4639	0.069	< 0.001	***
Model 1[†]	LVEF	3302	-0.107	< 0.001	***
	HbA1c	2426	0.159	< 0.001	***
	Glucose	4456	0.085	< 0.001	***
	BMI	4791	0.055	< 0.001	***
	hs-CRP	4452	0.162	< 0.001	***
	Leukocyte count	4639	0.133	< 0.001	***
Model 2[‡]	LVEF	3302	-0.104	< 0.001	***
	HbA1c	2426	0.082	< 0.001	***
	Glucose	4456	0.046	0.003	**
	BMI	4791	0.030	0.036	*
	hs-CRP	4452	0.149	< 0.001	***
	Leukocyte count	4639	0.133	< 0.001	***
Model 3[§]	LVEF	3301	-0.104	< 0.001	***
	HbA1c	2426	0.083	< 0.001	***
	Glucose	4455	0.045	0.003	**
	BMI	4790	0.031	0.034	*
	hs-CRP	4452	0.149	< 0.001	***
	Leukocyte count	4638	0.133	< 0.001	***
**Histidine**	**Variable**	**No. of patients**	**Partial correlation coefficient**	***P-value***	**Symbol**
Unadjusted	LVEF	3302	0.078	< 0.001	***
	HbA1c	2426	-0.009	0.655	
	Glucose	4456	-0.119	< 0.001	***
	BMI	4791	0.027	0.095	
	hs-CRP	4452	-0.172	< 0.001	***
	Leukocyte count	4639	-0.137	< 0.001	***
Model 1[†]	LVEF	3302	0.081	< 0.001	***
	HbA1c	2426	0.011	0.635	
	Glucose	4456	-0.117	< 0.001	***

	BMI	4791	0.012	0.459	
	hs-CRP	4452	-0.159	< 0.001	***
	Leukocyte count	4639	-0.166	< 0.001	***
Model 2‡	LVEF	3302	0.079	< 0.001	***
	HbA1c	2426	0.021	0.364	
	Glucose	4456	-0.113	< 0.001	***
	BMI	4791	0.019	0.275	
	hs-CRP	4452	-0.157	< 0.001	***
	Leukocyte count	4639	-0.165	< 0.001	***
Model 3§	LVEF	3301	0.079	< 0.001	***
	HbA1c	2426	0.021	0.364	
	Glucose	4455	-0.113	< 0.001	***
	BMI	4790	0.018	0.297	
	hs-CRP	4452	-0.156	< 0.001	***
	Leukocyte count	4638	-0.165	< 0.001	***

**Urocanate**	**Variable**	**No. of patients**	**Partial correlation coefficient**	***P-value***	**Symbol**
Unadjusted	LVEF	3302	0.007	0.768	
	HbA1c	2426	-0.058	0.011	*
	Glucose	4456	-0.056	0.001	***
	BMI	4791	-0.049	0.003	**
	hs-CRP	4452	0.012	0.676	
	Leukocyte count	4639	-0.063	< 0.001	***
Model 1†	LVEF	3302	0.006	0.768	
	HbA1c	2426	-0.068	0.003	**
	Glucose	4456	-0.058	0.001	**
	BMI	4791	-0.043	0.008	**
	hs-CRP	4452	0.005	0.768	
	Leukocyte count	4639	-0.052	< 0.001	***
Model 2‡	LVEF	3302	0.005	0.768	
	HbA1c	2426	-0.052	0.018	*
	Glucose	4456	-0.054	0.001	**
	BMI	4791	-0.039	0.016	*
	hs-CRP	4452	0.007	0.768	
	Leukocyte count	4639	-0.052	< 0.001	***
Model 3§	LVEF	3301	0.006	0.768	
	HbA1c	2426	-0.051	0.021	*
	Glucose	4455	-0.054	0.001	**
	BMI	4790	-0.038	0.017	*

	hs-CRP	4452	0.007	0.768	
	Leukocyte count	4638	-0.052	< 0.001	***

Correlation analyses were performed using the Spearman rank-correlation method. * False discovery rate (FDR) p< 0.05, ** FDR adjustment for p< 0.01, *** FDR adjustment for p< 0.001. †Model 1 was adjusted for age and sex. ‡Model 2 was adjusted for age, sex, and diabetes. §Model 3 was adjusted for age, sex, diabetes, and hypercholesterolaemia. ACS, acute coronary syndrome; BMI, body mass index; CAD, coronary artery disease; CCS, chronic coronary syndrome; HbA1c, haemoglobin A1c; hs-CRP, high-sensitivity C-reactive protein; LVEF, left ventricular ejection fraction.

Table S14: Correlation of imidazole propionate, histidine, and urocanate levels with coronary microvascular obstruction at 30 days after primary percutaneous coronary intervention

ImP	Variable	No. of patients	Partial correlation coefficient	*P-value*	Symbol
Unadjusted	Coronary MVO at 30 days, g	60	0.267	0.040	*
	Coronary MVO at 30 day, % of LV mass	59	0.282	0.040	*
Model 1†	Coronary MVO at 30 days, g	60	0.283	0.040	*
	Coronary MVO at 30 day, % of LV mass	59	0.301	0.040	*
Model 2‡	Coronary MVO at 30 days, g	48	0.319	0.040	*
	Coronary MVO at 30 day, % of LV mass	48	0.320	0.040	*
Model 3§	Coronary MVO at 30 days, g	48	0.338	0.040	*
	Coronary MVO at 30 day, % of LV mass	48	0.333	0.040	*
**Histidine**	**Variable**	**No. of patients**	**Partial correlation coefficient**	***P-value***	**Symbol**
Unadjusted	Coronary MVO at 30 days, g	60	-0.169	0.523	
	Coronary MVO at 30 day, % of LV mass	59	-0.216	0.523	
Model 1†	Coronary MVO at 30 days, g	60	-0.126	0.350	
	Coronary MVO at 30 day, % of LV mass	59	-0.166	0.222	
Model 2‡	Coronary MVO at 30 days, g	48	-0.111	0.485	
	Coronary MVO at 30 day, % of LV mass	48	-0.155	0.328	
Model 3§	Coronary MVO at 30 days, g	48	-0.082	0.613	
	Coronary MVO at 30 day, % of LV mass	48	-0.139	0.392	
**Urocanate**	**Variable**	**No. of patients**	**Partial correlation coefficient**	***P-value***	**Symbol**
Unadjusted	Coronary MVO at 30 days, g	60	0.016	0.984	
	Coronary MVO at 30 day, % of LV mass	59	-0.041	0.984	
Model 1†	Coronary MVO at 30 days, g	60	0.003	0.984	
	Coronary MVO at 30 day, % of LV mass	59	-0.050	0.717	
Model 2‡	Coronary MVO at 30 days, g	48	0.105	0.507	
	Coronary MVO at 30 day, % of LV mass	48	0.069	0.663	
Model 3§	Coronary MVO at 30 days, g	48	0.097	0.550	
	Coronary MVO at 30 day, % of LV mass	48	0.063	0.700	

Correlation analyses were performed using the Spearman rank-correlation method. P-values were false discovery rate-adjusted using Benjamini-Hochberg correction. * $P < 0.05$, ** $P < 0.01$, *** $P < 0.001$. †Model 1 was adjusted for age, sex, and diabetes status. ‡Model 2 was adjusted for age, sex, diabetes status, hypertension, low-density lipoprotein cholesterol, and glucose. §Model 3 was adjusted for age, sex, diabetes status, hypertension, low-density lipoprotein cholesterol, glucose, and pre-procedural TIMI-flow. ImP, imidazole propionate; LV, left ventricular; MVO, microvascular obstruction; TIMI, thrombolysis in myocardial infarction.

Table S15: Association of circulating imidazole propionate with MACE

**Swiss ACS cohort**	**No. of patients**	**No. of events**	**HR for MACE at 1 year**	**95% CI**	***P-value***
ImP, continuous*					
Crude	4318	314	1.50	1.38 – 1.62	< 0.001
Sex-, age- and diabetes-adjusted	4318	314	1.31	1.20 – 1.43	< 0.001
Fully adjusted	3966	288	1.22	1.10 – 1.35	< 0.001
Fully adjusted + histidine	3966	288	1.22	1.11 – 1.35	< 0.001
Fully adjusted + urocanate	3966	288	1.21	1.09 – 1.34	< 0.001
Fully adjusted + histidine and urocanate	3966	288	1.21	1.10 – 1.34	< 0.001
Fully adjusted + lipoprotein (a)	1253	78	1.33	1.09 – 1.62	0.004
Fully adjusted + history of chronic liver disease	3966	288	1.22	1.10 – 1.35	< 0.001
Fully adjusted + leucocyte count	3759	270	1.21	1.09 – 1.34	< 0.001
ImP, quartile§					
Crude	4318	314	2.19	1.75 – 2.74	< 0.001
Sex-, age- and diabetes-adjusted	4318	314	1.57	1.24 – 1.98	< 0.001
Fully adjusted	3966	288	1.33	1.03 – 1.72	0.032
Fully adjusted + histidine	3966	288	1.33	1.03 – 1.72	0.030
Fully adjusted + urocanate	3966	288	1.30	1.01 – 1.69	0.044
Fully adjusted + histidine and urocanate	3966	288	1.31	1.01 – 1.69	0.042
Fully adjusted + lipoprotein (a)	1253	78	1.76	1.09 – 2.85	0.020
Fully adjusted + history of chronic liver disease	3966	288	1.32	1.02 – 1.71	0.034
Fully adjusted + leucocyte count	3759	270	1.32	1.01 – 1.72	0.043
**German ACS cohort**	**No. of observations**	**No. of events**	**HR for MACE at 1 year**	**95% CI**	***P-value***
ImP, continuous*					
Crude	256	117	2.58	1.84 – 3.63	< 0.001
Sex-, age- and diabetes-adjusted	250	112	2.51	1.73 – 3.64	< 0.001
Fully adjusted	249	112	2.34	1.46 – 3.76	< 0.001
Fully adjusted + histidine	249	112	2.34	1.44 – 3.80	0.001
Fully adjusted + urocanate	249	112	2.33	1.44 – 3.76	0.001
Fully adjusted + histidine and urocanate	249	112	2.33	1.43 – 3.80	0.001
Fully adjusted + history of chronic liver disease	249	112	2.39	1.48 – 3.87	< 0.001
Fully adjusted + leucocyte count	249	112	2.33	1.47 – 3.70	< 0.001
ImP, quartile§					
Crude	256	117	13.24	5.36 – 32.67	< 0.001
Sex-, age- and diabetes-adjusted	250	112	12.23	4.79 – 31.21	< 0.001

Fully adjusted	249	112	7.97	2.70 – 23.52	< 0.001
Fully adjusted + histidine	249	112	7.88	2.68 – 23.18	< 0.001
Fully adjusted + urocanate	249	112	7.93	2.59 – 24.33	< 0.001
Fully adjusted + histidine and urocanate	249	112	7.83	2.56 – 23.94	< 0.001
Fully adjusted + history of chronic liver disease	249	112	8.14	2.78 – 23.84	< 0.001
Fully adjusted + leucocyte count	248	111	7.96	2.70 – 23.47	< 0.001
**German CCS cohort**	**No. of patients**	**No. of events**	**HR for MACE at 3 years**	**95% CI**	***P-value***
ImP, continuous*					
Crude	577	164	1.38	1.26 – 1.53	< 0.001
Sex-, age- and diabetes-adjusted	577	164	1.28	1.15 – 1.42	< 0.001
Fully adjusted	385	113	1.32	1.13 – 1.53	< 0.001
Fully adjusted + histidine	385	113	1.31	1.13 – 1.52	< 0.001
Fully adjusted + urocanate	385	113	1.30	1.12 – 1.51	0.001
Fully adjusted + histidine and urocanate	385	113	1.30	1.11 – 1.51	0.001
Fully adjusted + lipoprotein (a)	383	113	1.31	1.13 – 1.52	< 0.001
Fully adjusted + history of chronic liver disease	385	113	1.32	1.14 – 1.54	< 0.001
Fully adjusted + leucocyte count	384	113	1.30	1.12 – 1.52	0.001
Fully adjusted + ethnicity	385	113	1.32	1.13 – 1.53	< 0.001
ImP, quartile§					
Crude	577	164	2.52	1.85 – 3.43	< 0.001
Sex-, age- and diabetes-adjusted	577	164	1.95	1.41 – 2.69	< 0.001
Fully adjusted	385	113	1.83	1.22 – 2.74	0.004
Fully adjusted + histidine	385	113	1.79	1.19 – 2.70	0.005
Fully adjusted + urocanate	385	113	1.76	1.17 – 2.65	0.007
Fully adjusted + histidine and urocanate	385	113	1.75	1.16 – 2.64	0.008
Fully adjusted + lipoprotein (a)	383	113	1.80	1.20 – 2.71	0.004
Fully adjusted + history of chronic liver disease	385	113	1.84	1.22 – 2.77	0.003
Fully adjusted + leucocyte count	384	113	1.80	1.20 – 2.71	0.005
Fully adjusted + ethnicity	385	113	1.83	1.22 – 2.75	0.004

*Hazard ratios refer to log2 increases in continuous ImP level; i.e., values can be interpreted as per doubling in ImP level. §quartile 4 (>24.4 nM) vs. quartile 1 – 3 (0–24.4 nM). ACS, acute coronary syndrome; CCS, chronic coronary syndrome; CI, confidence interval; HR, hazard ratio; ImP, imidazole propionate; MACE, major adverse cardiovascular events.

Table S16: Association of circulating imidazole propionate with mortality

**Swiss ACS cohort**	**No. of patients**	**No. of events**	**HR for mortality at 1 year**	**95% CI**	***P-value***
ImP, continuous*					
Crude	4318	148	1.68	1.53 – 1.85	< 0.001
Sex-, age- and diabetes-adjusted	4318	148	1.43	1.28 – 1.6	< 0.001
Fully adjusted	3966	128	1.34	1.17 – 1.54	< 0.001
Fully adjusted + GRACE 2.0	3532	109	1.24	1.06 – 1.46	0.007
Fully adjusted + histidine	3966	128	1.34	1.16 – 1.53	< 0.001
Fully adjusted + urocanate	3966	128	1.34	1.17 – 1.53	< 0.001
Fully adjusted + histidine and urocanate	3966	128	1.33	1.16 – 1.53	< 0.001
Fully adjusted + lipoprotein (a)	1253	35	1.59	1.19 – 2.13	0.002
Fully adjusted + history of chronic liver disease	3966	128	1.34	1.17 – 1.54	< 0.001
Fully adjusted + leucocyte count	3759	119	1.34	1.16 – 1.54	< 0.001
ImP, quartile§					
Crude	4318	148	3.74	2.7 – 5.16	< 0.001
Sex-, age- and diabetes-adjusted	4318	148	2.41	1.72 – 3.38	< 0.001
Fully adjusted	3966	128	1.86	1.28 – 2.72	0.001
Fully adjusted + GRACE 2.0	3532	109	1.47	0.96 – 2.26	0.076
Fully adjusted + histidine	3966	128	1.85	1.26 – 2.70	0.002
Fully adjusted + urocanate	3966	128	1.86	1.27 – 2.72	0.001
Fully adjusted + histidine and urocanate	3966	128	1.84	1.26 – 2.70	0.002
Fully adjusted + lipoprotein (a)	1253	35	2.82	1.36 – 5.87	0.005
Fully adjusted + history of chronic liver disease	3966	128	1.86	1.27 – 2.72	0.001
Fully adjusted + leucocyte count	3759	119	1.92	1.29 – 2.85	0.001
**German ACS cohort**	**No. of observations**	**No. of events**	**HR for mortality at 1 year**	**95% CI**	***P-value***
ImP, continuous*					
Crude	214	75	2.63	1.67 – 4.14	< 0.001
Sex-, age- and diabetes-adjusted	209	71	2.67	1.68 – 4.23	< 0.001
Fully adjusted	208	71	2.38	1.48 – 3.82	< 0.001
Fully adjusted + GRACE 2.0	208	71	2.32	1.46 – 3.41	< 0.001
Fully adjusted + histidine	208	71	2.37	1.47 – 3.82	< 0.001
Fully adjusted + urocanate	208	71	2.39	1.48 – 3.84	< 0.001
Fully adjusted + histidine and urocanate	208	71	2.39	1.48 – 3.86	< 0.001
Fully adjusted + history of chronic liver disease	208	71	2.43	1.48 – 4.00	< 0.001
Fully adjusted + leucocyte count	207	70	2.29	1.51 – 3.49	< 0.001
ImP, quartile§					

Crude	214	75	13.28	4.52 – 39.03	< 0.001
Sex-, age- and diabetes-adjusted	209	71	13.19	4.34 – 40.08	< 0.001
Fully adjusted	208	71	6.02	1.64 – 22.18	0.007
Fully adjusted + GRACE 2.0	208	71	4.84	1.42 – 16.47	0.012
Fully adjusted + histidine	208	71	5.80	1.55 – 21.64	0.009
Fully adjusted + urocanate	208	71	5.98	1.60 – 22.29	0.008
Fully adjusted + histidine and urocanate	208	71	5.78	1.53 – 21.84	0.010
Fully adjusted + history of chronic liver disease	208	71	6.02	1.53 – 23.61	0.010
Fully adjusted + leucocyte count	207	70	5.94	1.71 – 20.71	0.005
**German CCS cohort**	**No. of patients**	**No. of events**	**HR for mortality at 3 years**	**95% CI**	***P-value***
ImP, continuous*					
Crude	577	47	1.46	1.23 – 1.74	0
Sex-, age- and diabetes-adjusted	577	47	1.28	1.05 – 1.55	0.013
Fully adjusted	385	33	1.50	1.14 – 1.98	0.004
Fully adjusted + histidine	385	33	1.51	1.15 – 2.00	0.003
Fully adjusted + urocanate	385	33	1.49	1.13 – 1.98	0.005
Fully adjusted + histidine and urocanate	385	33	1.50	1.13 – 1.99	0.005
Fully adjusted + lipoprotein (a)	383	33	1.52	1.16 – 2.00	0.003
Fully adjusted + history of chronic liver disease	385	33	1.48	1.12 – 1.96	0.006
Fully adjusted + leucocyte count	384	33	1.54	1.16 – 2.04	0.003
Fully adjusted + ethnicity	385	33	1.50	1.14 – 1.97	0.004
ImP, quartile§					
Crude	577	47	2.62	1.48 – 4.65	0.001
Sex-, age- and diabetes-adjusted	577	47	1.66	0.91 – 3.04	0.099
Fully adjusted	385	33	2.01	0.96 – 4.19	0.063
Fully adjusted + histidine	385	33	2.04	0.97 – 4.27	0.060
Fully adjusted + urocanate	385	33	1.96	0.93 – 4.11	0.077
Fully adjusted + histidine and urocanate	385	33	1.99	0.94 – 4.19	0.072
Fully adjusted + lipoprotein (a)	383	33	2.10	1.01 – 4.37	0.047
Fully adjusted + history of chronic liver disease	385	33	1.94	0.93 – 4.05	0.076
Fully adjusted + leucocyte count	384	33	2.02	0.97 – 4.23	0.061
Fully adjusted + ethnicity	385	33	2.00	0.96 – 4.16	0.066

*Hazard ratios refer to log2 increases in continuous ImP level; i.e., values can be interpreted as per doubling in ImP level. §quartile 4 (>24.4 nM) vs. quartile 1 – 3 (0–24.4 nM). ACS, acute coronary syndrome; CCS, chronic coronary syndrome; CI, confidence interval; GRACE, Global Registry of Acute Coronary Events score, HR, hazard ratio; ImP, imidazole propionate.

Table S17: Association of circulating imidazole propionate with MACE in subgroups

Swiss ACS cohort	No. of patients	No. of events	HR for MACE at 1 year	95% CI	*P-value*
ImP, continuous*					
Crude					
Male	3437	238	1.50	1.37 – 1.66	<0.001
Female	881	76	1.47	1.28 – 1.68	<0.001
Age ≤ 70	3010	146	1.40	1.22 – 1.60	<0.001
Age > 70	1308	168	1.40	1.26 – 1.56	<0.001
No diabetes	3565	213	1.44	1.29 – 1.61	<0.001
Diabetes	753	101	1.41	1.25 – 1.59	<0.001
No PPI use	2134	179	1.41	1.26 – 1.58	<0.001
PPI use	643	66	1.49	1.28 – 1.72	<0.001
Sex-, age- and diabetes-adjusted					
Male	3437	238	1.28	1.15 – 1.42	<0.001
Female	881	76	1.39	1.20 – 1.62	<0.001
Age ≤ 70	3010	146	1.34	1.17 – 1.53	<0.001
Age > 70	1308	168	1.35	1.21 – 1.51	<0.001
No diabetes	3565	213	1.30	1.16 – 1.46	<0.001
Diabetes	753	101	1.34	1.18 – 1.52	<0.001
No PPI use	2134	179	1.26	1.11 – 1.41	<0.001
PPI use	643	66	1.35	1.15 – 1.58	<0.001
Fully adjusted					
Male	3165	217	1.17	1.04 – 1.32	0.011
Female	801	71	1.33	1.10 – 1.60	0.003
Age ≤ 70	2787	135	1.22	1.04 – 1.42	0.012
Age > 70	1179	153	1.24	1.09 – 1.42	0.001
No diabetes	3280	194	1.16	1.01 – 1.32	0.031
Diabetes	686	94	1.30	1.12 – 1.52	0.001
No PPI use	1952	163	1.16	1.00 – 1.33	0.043
PPI use	595	62	1.34	1.10 – 1.63	0.003
**German CCS cohort**	**No. of patients**	**No. of events**	**HR for MACE at 3 years**	**95% CI**	***P-value***
ImP, continuous*					
Crude					
Male	453	130	1.45	1.30 – 1.62	<0.001
Female	124	34	1.21	0.99 – 1.49	0.060
Age ≤ 70	198	35	1.50	1.20 – 1.86	<0.001

Age > 70	379	129	1.31	1.17 – 1.47	<0.001
No diabetes	399	84	1.42	1.24 – 1.63	<0.001
Diabetes	178	80	1.23	1.05 – 1.43	0.010
No PPI use	330	87	1.37	1.19 – 1.58	<0.001
PPI use	247	77	1.39	1.21 – 1.60	<0.001
Low fruit and vegetable consumption	387	110	1.39	1.22 – 1.59	<0.001
High fruit and vegetable consumption	133	39	1.52	1.24 – 1.85	<0.001
Sex-, age- and diabetes-adjusted					
Male	453	130	1.34	1.19 – 1.51	<0.001
Female	124	34	1.11	0.89 – 1.39	0.358
Age ≤ 70	198	35	1.47	1.16 – 1.86	0.001
Age > 70	379	129	1.26	1.12 – 1.41	<0.001
No diabetes	399	84	1.37	1.18 – 1.58	<0.001
Diabetes	178	80	1.19	1.01 – 1.39	0.036
No PPI use	330	87	1.20	1.02 – 1.41	0.027
PPI use	247	77	1.33	1.15 – 1.55	<0.001
Low fruit and vegetable consumption	387	110	1.32	1.14 – 1.53	<0.001
High fruit and vegetable consumption	133	39	1.54	1.19 – 2.00	0.001
Fully adjusted					
Male	301	95	1.41	1.18 – 1.68	<0.001
Female	84	18	1.08	0.81 – 1.43	0.615
Age ≤ 70	129	22	1.45	1.05 – 2.01	0.024
Age > 70	256	91	1.31	1.11 – 1.55	0.002
No diabetes	266	60	1.46	1.17 – 1.81	0.001
Diabetes	119	53	1.28	1.01 – 1.61	0.039
No PPI use	221	57	1.28	1.02 – 1.60	0.031
PPI use	164	56	1.41	1.14 – 1.75	0.002
Low fruit and vegetable consumption	259	74	1.35	1.10 – 1.66	0.004
High fruit and vegetable consumption	85	28	1.68	1.21 – 2.33	0.002

*Hazard ratios refer to log2 increases in continuous ImP level; i.e., values can be interpreted as per doubling in ImP level. ACS, acute coronary syndrome; CCS, chronic coronary syndrome; CI, confidence interval; HR, hazard ratio; ImP, imidazole propionate; MACE, major adverse cardiovascular events; PPI, proton pump inhibitor.

Table S18: Association of circulating imidazole propionate with mortality in subgroups

**Swiss ACS cohort**	**No. of patients**	**No. of events**	**HR for mortality at 1 year**	**95% CI**	***P-value***
ImP, continuous*					
Crude					
Male	3437	108	1.68	1.50 – 1.88	<0.001
Female	881	40	1.65	1.39 – 1.96	<0.001
Age ≤ 70	3010	51	1.80	1.48 – 2.18	<0.001
Age > 70	1308	97	1.44	1.28 – 1.63	<0.001
No diabetes	3565	96	1.83	1.58 – 2.13	<0.001
Diabetes	753	52	1.41	1.22 – 1.64	<0.001
No PPI use	2134	83	1.69	1.45 – 1.96	<0.001
PPI use	643	37	1.42	1.20 – 1.69	<0.001
Sex-, age- and diabetes-adjusted					
Male	3437	108	1.39	1.21 – 1.58	<0.001
Female	881	40	1.53	1.26 – 1.87	<0.001
Age ≤ 70	3010	51	1.67	1.37 – 2.03	<0.001
Age > 70	1308	97	1.42	1.25 – 1.61	<0.001
No diabetes	3565	96	1.59	1.36 – 1.87	<0.001
Diabetes	753	52	1.33	1.13 – 1.55	<0.001
No PPI use	2134	83	1.42	1.21 – 1.66	<0.001
PPI use	643	37	1.37	1.12 – 1.67	0.002
Fully adjusted					
Male	3165	92	1.29	1.08 – 1.53	0.004
Female	801	36	1.47	1.14 – 1.89	0.003
Age ≤ 70	2787	41	1.48	1.15 – 1.91	0.002
Age > 70	1179	87	1.30	1.11 – 1.52	0.001
No diabetes	3280	81	1.36	1.12 – 1.64	0.002
Diabetes	686	47	1.34	1.09 – 1.64	0.005
No PPI use	1952	70	1.27	1.04 – 1.56	0.021
PPI use	595	35	1.46	1.14 – 1.86	0.002
Fully adjusted + GRACE 2.0					
Male	2831	78	1.18	0.97 – 1.44	0.104
Female	701	31	1.30	0.98 – 1.72	0.065
Age ≤ 70	2504	37	1.46	1.10 – 1.94	0.009
Age > 70	1028	72	1.15	0.95 – 1.39	0.143
No diabetes	2931	66	1.22	0.98 – 1.52	0.077
Diabetes	601	43	1.30	1.03 – 1.65	0.030

No PPI use	1761	63	1.20	0.97 – 1.49	0.098
PPI use	518	27	1.37	1.01 – 1.84	0.041
**German CCS cohort**	**No. of patients**	**No. of events**	**HR for mortality at 3 years**	**95% CI**	***P-value***
ImP, continuous*					
Crude					
Male	453	38	1.46	1.19 – 1.79	<0.001
Female	124	9	1.49	1.06 – 2.10	0.023
Age ≤ 70	198	5	2.08	1.28 – 3.39	0.003
Age > 70	379	42	1.32	1.08 – 1.60	0.006
No diabetes	399	25	1.65	1.32 – 2.07	<0.001
Diabetes	178	22	1.15	0.85 – 1.55	0.356
No PPI use	330	30	1.44	1.14 – 1.82	0.002
PPI use	247	17	1.59	1.20 – 2.10	0.001
Low fruit and vegetable consumption	387	33	1.44	1.14 – 1.82	0.002
High fruit and vegetable consumption	133	13	1.80	1.30 – 2.48	<0.001
Sex-, age- and diabetes-adjusted					
Male	453	38	1.26	0.99 – 1.59	0.059
Female	124	9	1.30	0.91 – 1.84	0.145
Age ≤ 70	198	5	2.14	1.31 – 3.47	0.002
Age > 70	379	42	1.28	1.04 – 1.57	0.020
No diabetes	399	25	1.54	1.20 – 1.97	0.001
Diabetes	178	22	0.98	0.70 – 1.37	0.900
No PPI use	330	30	1.14	0.87 – 1.49	0.347
PPI use	247	17	1.51	1.12 – 2.04	0.007
Low fruit and vegetable consumption	387	33	1.37	1.04 – 1.81	0.023
High fruit and vegetable consumption	133	13	2.04	1.32 – 3.15	0.001
Fully adjusted					
Male	301	29	1.40	1.02 – 1.92	0.036
Female	84	4	1.67	0.94 – 3.00	0.083
Age ≤ 70	129	3	1.62	0.72 – 3.62	0.242
Age > 70	256	30	1.52	1.13 – 2.05	0.005
No diabetes	266	18	2.01	1.37 – 2.95	<0.001
Diabetes	119	15	1.09	0.69 – 1.73	0.715
No PPI use	221	20	1.49	1.02 – 2.17	0.040
PPI use	164	13	1.60	1.05 – 2.43	0.030
Low fruit and vegetable consumption	259	22	1.36	0.94 – 1.95	0.101

High fruit and vegetable consumption	85	10	3.63	1.74 – 7.58	0.001

*Hazard ratios refer to log2 increases in continuous ImP level; i.e., values can be interpreted as per doubling in ImP level. ACS, acute coronary syndrome; CCS, chronic coronary syndrome; CI, confidence interval; GRACE, Global Registry of Acute Coronary Events score; HR, hazard ratio; ImP, imidazole propionate; PPI, proton pump inhibitor.

Table S19: Sensitivity analysis using cohort-specific quartile thresholds

**German ACS cohort**	**No. of observations**	**No. of events**	**HR for MACE at 1 year**	**95% CI**	***P-value***
ImP, quartile§					
Crude	256	117	14.10	5.60 – 35.50	< 0.001
Sex-, age- and diabetes-adjusted	250	112	13.86	5.24 – 36.68	< 0.001
Fully adjusted	249	112	12.07	3.51 – 41.56	< 0.001
Fully adjusted + histidine	249	112	12.64	3.57 – 44.79	< 0.001
Fully adjusted + urocanate	249	112	12.11	3.44 – 42.63	< 0.001
Fully adjusted + histidine and urocanate	249	112	12.57	3.48 – 45.40	< 0.001
Fully adjusted + history of chronic liver disease	249	112	12.59	3.61 – 43.90	< 0.001
Fully adjusted + leucocyte count	248	111	12.50	3.66 – 42.71	< 0.001
**German ACS cohort**	**No. of observations**	**No. of events**	**HR for mortality at 1 year**	**95% CI**	***P-value***
ImP, quartile§					
Crude	214	75	19.57	6.65 – 57.31	< 0.001
Sex-, age- and diabetes-adjusted	209	71	23.38	7.17 – 76.23	< 0.001
Fully adjusted	208	71	14.90	2.81 – 79.05	0.002
Fully adjusted + GRACE 2.0	208	71	10.95	2.40 – 49.90	0.002
Fully adjusted + histidine	208	71	15.19	2.86 – 80.60	0.001
Fully adjusted + urocanate	208	71	14.77	2.77 – 78.62	0.002
Fully adjusted + histidine and urocanate	208	71	15.37	2.79 – 84.76	0.002
Fully adjusted + history of chronic liver disease	208	71	15.94	2.60 – 97.61	0.003
Fully adjusted + leucocyte count	207	70	15.13	2.97 – 77.15	0.001
**German CCS cohort**	**No. of patients**	**No. of events**	**HR for MACE at 3 years**	**95% CI**	***P-value***
ImP, quartile#					
Crude	577	164	2.29	1.68 – 3.14	< 0.001
Sex-, age- and diabetes-adjusted	577	164	1.75	1.26 – 2.43	0.001
Fully adjusted	385	113	1.69	1.13 – 2.55	0.012
Fully adjusted + histidine	385	113	1.67	1.10 – 2.52	0.015
Fully adjusted + urocanate	385	113	1.64	1.09 – 2.48	0.018
Fully adjusted + histidine and urocanate	385	113	1.63	1.07 – 2.46	0.022
Fully adjusted + lipoprotein (a)	383	113	1.67	1.11 – 2.53	0.014
Fully adjusted + History of chronic liver disease	385	113	1.71	1.13 – 2.58	0.011
Fully adjusted + leucocyte count	384	113	1.67	1.11 – 2.52	0.015

Fully adjusted + ethnicity	385	113	1.70	1.12 – 2.56	0.012
**German CCS cohort**	**No. of patients**	**No. of events**	**HR for mortality at 3 years**	**95% CI**	***P-value***
ImP, quartile#					
Crude	577	47	2.57	1.45 – 4.57	0.001
Sex-, age- and diabetes-adjusted	577	47	1.61	0.87 – 2.96	0.127
Fully adjusted	385	33	2.14	1.02 – 4.48	0.043
Fully adjusted + histidine	385	33	2.16	1.03 – 4.54	0.042
Fully adjusted + urocanate	385	33	2.10	1.00 – 4.41	0.051
Fully adjusted + histidine and urocanate	385	33	2.12	1.00 – 4.47	0.049
Fully adjusted + lipoprotein (a)	383	33	2.20	1.06 – 4.58	0.035
Fully adjusted + history of chronic liver disease	385	33	2.06	0.98 – 4.31	0.056
Fully adjusted + leukocyte count	384	33	2.16	1.03 – 4.53	0.041
Fully adjusted + ethnicity	385	33	2.13	1.02 – 4.46	0.045

§Cohort-specific quartile of the German ACS cohort: quartile 4 (ImP >20.0 nM) vs. quartile 1 – 3 (ImP 0–20.0 nM). #Cohort-specific quartile of the German CCS cohort: quartile 4 (ImP >26.0 nM) vs. quartile 1 – 3 (ImP 0–26.0 nM). ACS, acute coronary syndrome; CCS, chronic coronary syndrome; CI, confidence interval; GRACE, Global Registry of Acute Coronary Events score; HR, hazard ratio; ImP, imidazole propionate; MACE, major adverse cardiovascular events.

Table S20: Summary of missing data according to study cohort

	Swiss ACS cohort	Swiss CMR cohort	German ACS cohort		German CCS cohort
	(*n* = 4318)	(*n* = 60)	(Subcohort; *n* = 139)	(Patients with MACE at 1 year; *n* = 117)	(*n* = 577)
Predictors					
Sex	4318/4318 (100.0%)	60/60 (100.0%)	139/139 (100.0%)	117/117 (100.0%)	577/577 (100.0%)
Age	4318/4318 (100.0%)	60/60 (100.0%)	139/139 (100.0%)	117/117 (100.0%)	577/577 (100.0%)
Ethnicity	..	..	..	..	577/577 (100.0%)
Diabetes	4318/4318 (100.0%)	60/60 (100.0%)	138/139 (99.3%)	112/117 (95.7%)	577/577 (100.0%)
Hypertension	4316/4318 (100.0%)	60/60 (100.0%)	139/139 (100.0%)	117/117 (100.0%)	577/577 (100.0%)
Hypercholesterolaemia	4317/4318 (100.0%)	60/60 (100.0%)	135/139 (97.1%)	107/117 (91.5%)	577/577 (100.0%)
Chronic liver disease	4318/4318 (100.0%)	60/60 (100.0%)	139/139 (100.0%)	117/117 (100.0%)	577/577 (100.0%)
BMI	4253/4318 (98.5%)	60/60 (100.0%)	102/139 (73.4%)	78/117 (66.7%)	538/577 (93.2%)
ACS type	4318/4318 (100.0%)	60/60 (100.0%)	139/139 (100.0%)	117/117 (100.0%)	..
Glucose	4002/4318 (92.7%)	49/60 (81.7%)	138/139 (99.3%)	117/117 (100.0%)	454/577 (78.7%)
HbA1c	1849/4318 (42.8%)	..	72/139 (51.8%)	41/117 (35.0%)	577/577 (100.0%)
Leukocytes	4076/4318 (94.4%)	58/60 (96.7%)	139/139 (100.0%)	116/117 (99.1%)	563/577 (97.6%)
hs-TnT	4046/4318 (93.7%)	56/60 (93.3%)	139/139 (100.0%)	117/117 (100.0%)	220/577 (38.1%)
hs-CRP	4040/4318 (93.6%)	..	138/139 (99.3)	117/117 (100.0%)	412/577 (71.4%)
eGFR	4304/4318 (99.7%)	57/60 (95.0%)	139/139 (100.0%)	117/117 (100.0%)	577/577 (100.0%)
LVEF	2725/4318 (63.1%)	59/60 (98.3%)	138/139 (99.3)	117/117 (100.0%)	577/577 (100.0%)
PPI use	2777/4318 (64.3%)	58/60 (96.7%)	139/139 (100.0%)	117/117 (100.0%)	577/577 (100.0%)
Dietary habits	..	..	..	..	520/577 (90.1%)
Revascularization strategy during baseline visit	4318/4318 (100.0%)	60/60 (100.0%)	126/139 (90.6)	101/117 (86.3%)	577/577 (100.0%)
Pre-procedural TIMI-flow	3454/4318 (80.0%)	60/60 (100.0%)	..	..	..

Affected vessel	4095/4318 (94.8%)	60/60 (100.0%)	126/139 (90.6)	101/117 (86.3%)	577/577 (100.0%)
Outcomes					
MACE	4318/4318 (100.0%)	..	139/139 (100.0%)	139/139 (100.0%)	577/577 (100.0%)
Mortality	4318/4318 (100.0%)	..	139/139 (100.0%)	139/139 (100.0%)	577/577 (100.0%)
Coronary MVO at 30 days, g	..	60/60 (100.0%)	..	..	..
Coronary MVO at 30 day, % of LV mass	..	59/60 (98.3%)	..	..	..

Note that the subcohort includes 6 subjects with incident MACE and 3 subjects with incident death (see Supplementary table 4). ACS, acute coronary syndrome; BMI, body mass index; CCS, chronic coronary syndrome; CMR, cardiac magnetic resonance imaging; eGFR, estimated glomerular filtration rate; HbA1c, haemoglobin A1c; hs-CRP, high-sensitivity C-reactive protein; hs-TnT, high-sensitivity troponin T; LV, left ventricular; LVEF, left ventricular ejection fraction; MACE, major adverse cardiovascular events; MVO, microvascular obstruction; PPI, proton pump inhibitor; TIMI, thrombolysis in myocardial infarction.

Table S21: Association of imidazole propionate with MACE beyond TMAO

**Swiss ACS cohort**	**No. of patients**	**No. of events**	**HR for MACE at 1 year**	**95% CI**	***P-value***
ImP, continuous*					
Crude	1457	75	1.43	1.21 – 1.70	< 0.001
Sex-, age- and diabetes-adjusted	1457	75	1.32	1.10 – 1.58	0.003
Fully adjusted + TMAO	1281	72	1.30	1.05 – 1.61	0.014
TMAO, continuous*					
Crude	1457	75	1.35	1.12 – 1.62	0.002
Sex-, age- and diabetes-adjusted	1457	75	1.27	1.04 – 1.55	0.017
Fully adjusted + ImP	1281	72	1.21	0.95 – 1.53	0.117
**German CCS cohort**	**No. of patients**	**No. of events**	**HR for MACE at 3 years**	**95% CI**	***P-value***
ImP, continuous*					
Crude	577	164	1.30	1.18 – 1.44	< 0.001
Sex-, age- and diabetes-adjusted	577	164	1.23	1.10 – 1.37	< 0.001
Fully adjusted + TMAO	385	113	1.31	1.12 – 1.53	0.001
TMAO, continuous*					
Crude	577	164	1.26	1.13 – 1.40	< 0.001
Sex-, age- and diabetes-adjusted	577	164	1.21	1.08 – 1.36	0.001
Fully adjusted + ImP	385	113	1.35	1.18 – 1.55	< 0.001

*Hazard ratios refer to log2 increases in continuous ImP level; i.e., values can be interpreted as per doubling in ImP level. ACS, acute coronary syndrome; CCS, chronic coronary syndrome; CI, confidence interval; HR, hazard ratio; ImP, imidazole propionate; MACE, major adverse cardiovascular events; TMAO, trimethylamine N-oxide.

Table S22: Association of imidazole propionate with mortality beyond TMAO

**Swiss ACS cohort**	**No. of patients**	**No. of events**	**HR for mortality at 1 year**	**95% CI**	***P-value***
ImP, continuous*					
Crude	1457	34	1.56	1.24 – 1.96	< 0.001
Sex-, age- and diabetes-adjusted	1457	34	1.53	1.18 – 1.98	0.001
Fully adjusted + TMAO	1281	32	1.59	1.17 – 2.17	0.003
TMAO, continuous*					
Crude	1457	34	1.51	1.18 – 1.93	0.001
Sex-, age- and diabetes-adjusted	1457	34	1.49	1.14 – 1.95	0.003
Fully adjusted + ImP	1281	32	1.59	1.17 – 2.17	0.003
**German CCS cohort**	**No. of patients**	**No. of events**	**HR for mortality at 3 years**	**95% CI**	***P-value***
ImP, continuous*					
Crude	577	47	1.36	1.13 – 1.64	0.001
Sex-, age- and diabetes-adjusted	577	47	1.23	1.01 – 1.51	0.038
Fully adjusted + TMAO	385	33	1.48	1.12 – 1.96	0.006
TMAO, continuous*					
Crude	577	47	1.28	1.06 – 1.56	0.012
Sex-, age- and diabetes-adjusted	577	47	1.17	0.95 – 1.43	0.134
Fully adjusted + ImP	385	33	1.25	0.97 – 1.61	0.079

*Hazard ratios refer to log2 increases in continuous ImP level; i.e., values can be interpreted as per doubling in ImP level. ACS, acute coronary syndrome; CCS, chronic coronary syndrome; CI, confidence interval; HR, hazard ratio; ImP, imidazole propionate; TMAO, trimethylamine N-oxide.

Figure S1: Study flow chart

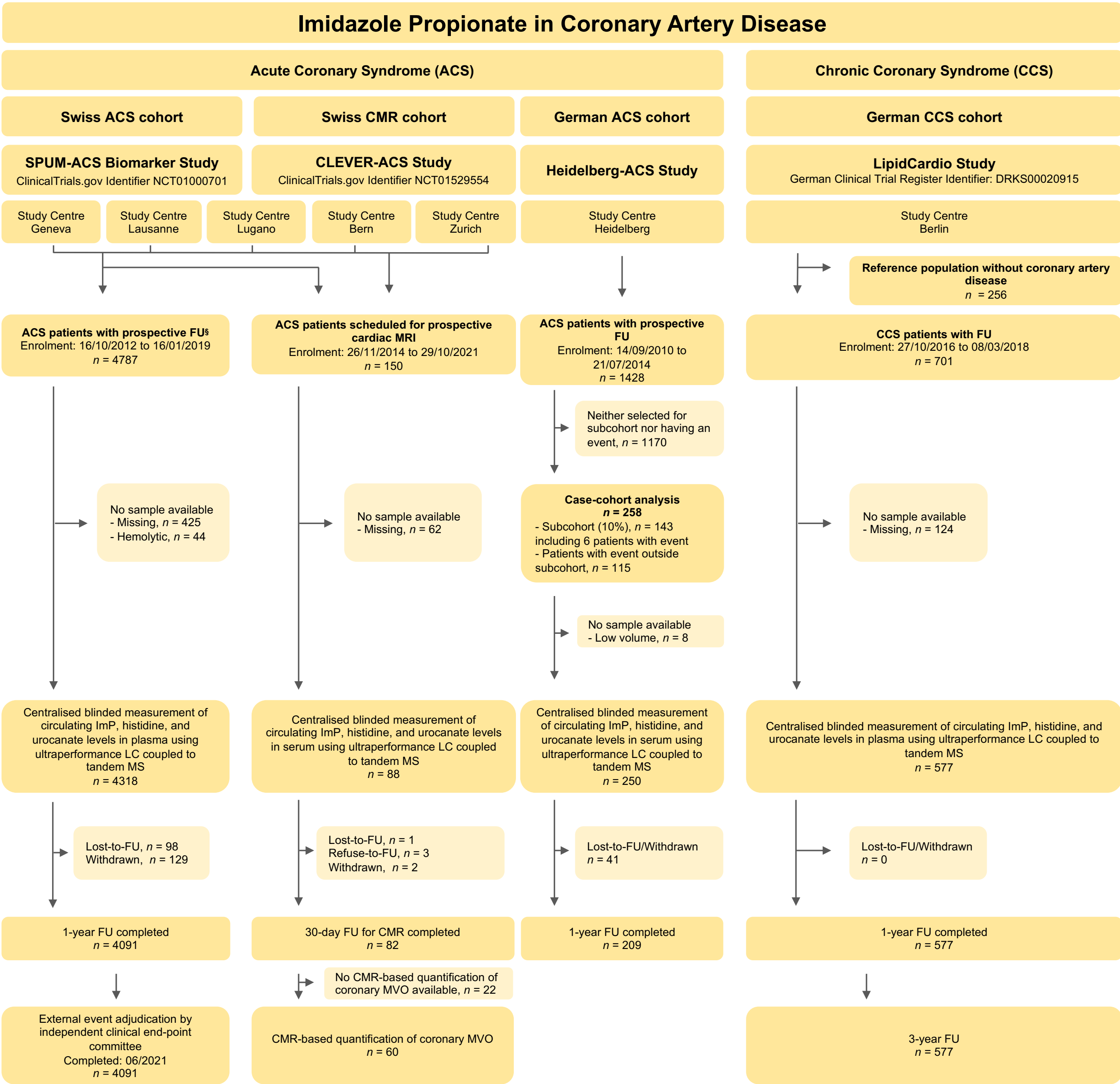

Figure S2: Analytical flow chart

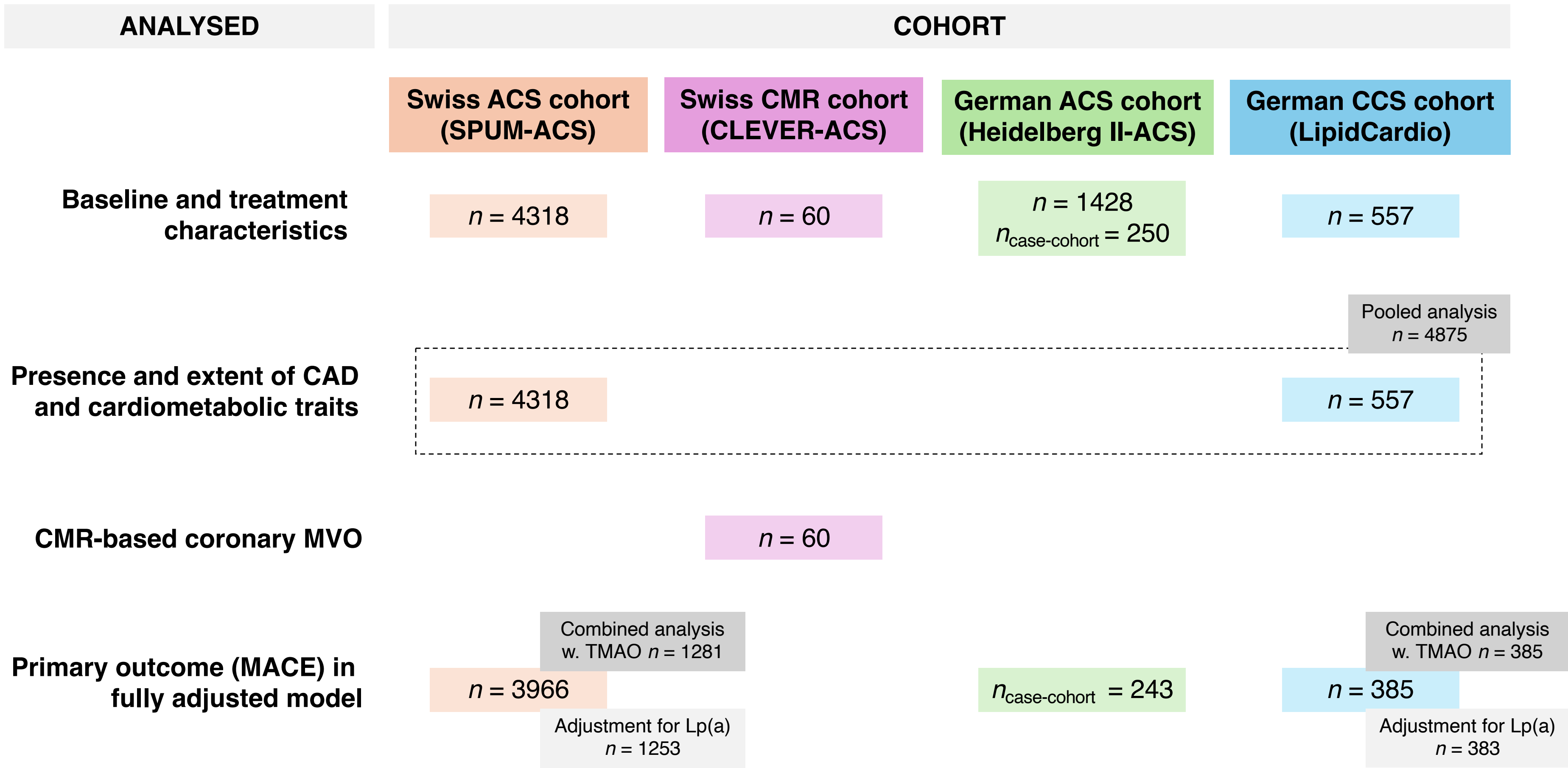

ACS, acute coronary syndrome; CAD, coronary artery disease; CCS, chronic coronary syndrome; CMR, cardiac magnetic resonance; Lp(a), lipoprotein(a); MACE, major adverse cardiovascular events; MVO, microvascular obstruction; TMAO, trimethylamine N-oxide

Figure S3: Imidazole propionate levels across different times of the day in Swiss ACS cohort

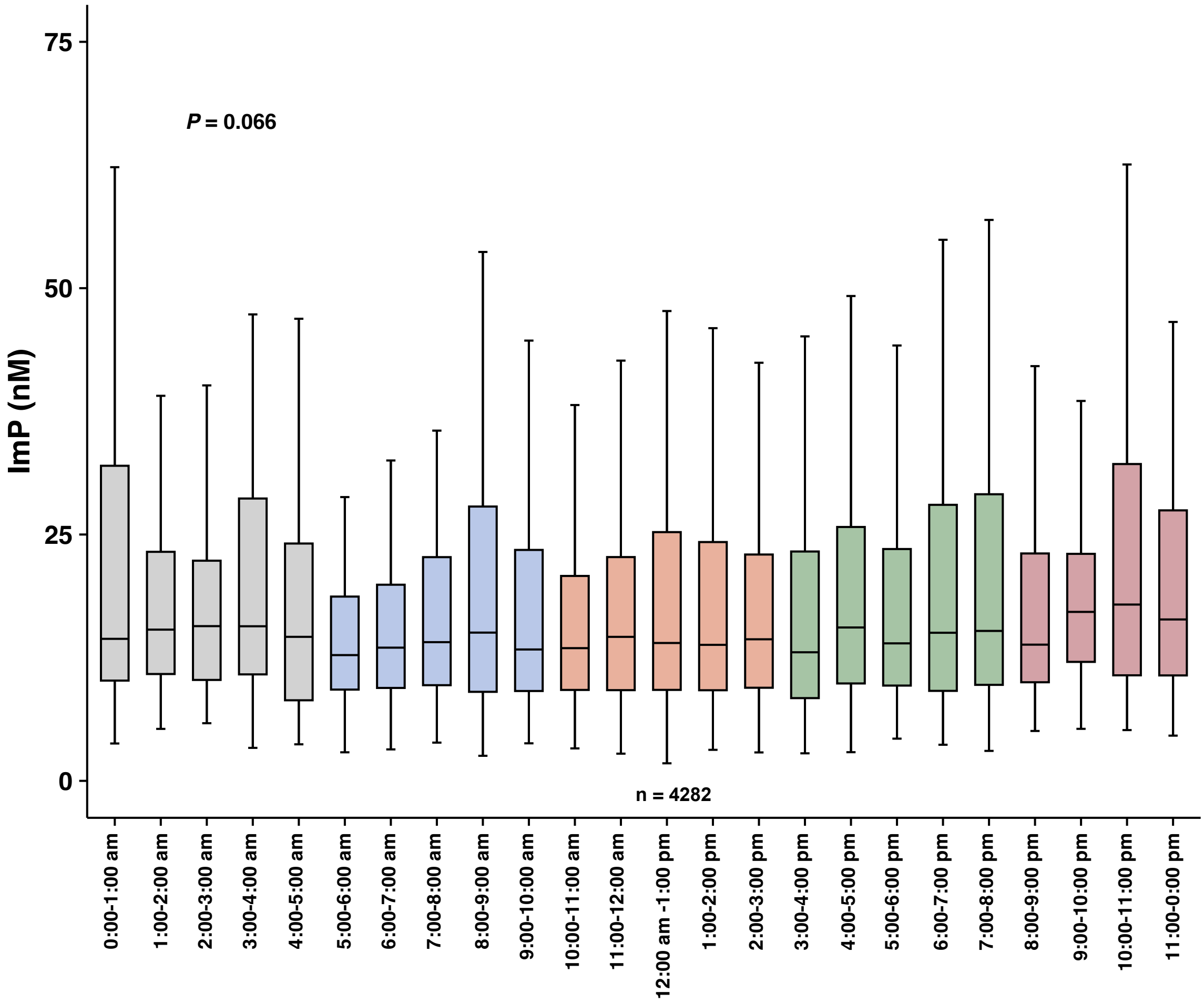

ACS, acute coronary syndrome; ImP, imidazole propionate.

Figure S4: Imidazole propionate levels across different times of the day in German CCS cohort

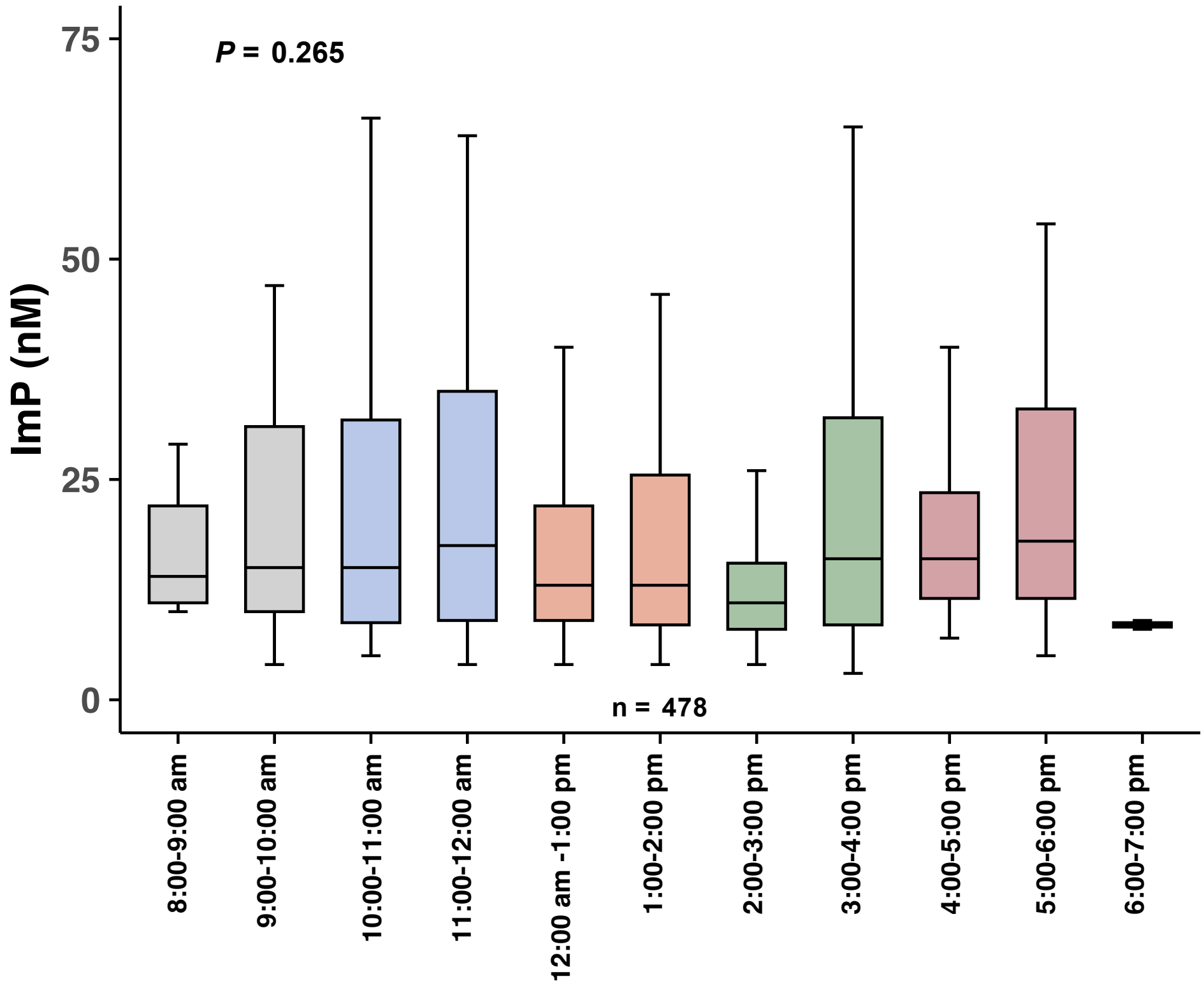

CCS, chronic coronary syndrome; ImP, imidazole propionate.

Figure S5: Imidazole propionate distribution stratified by clinical subgroup

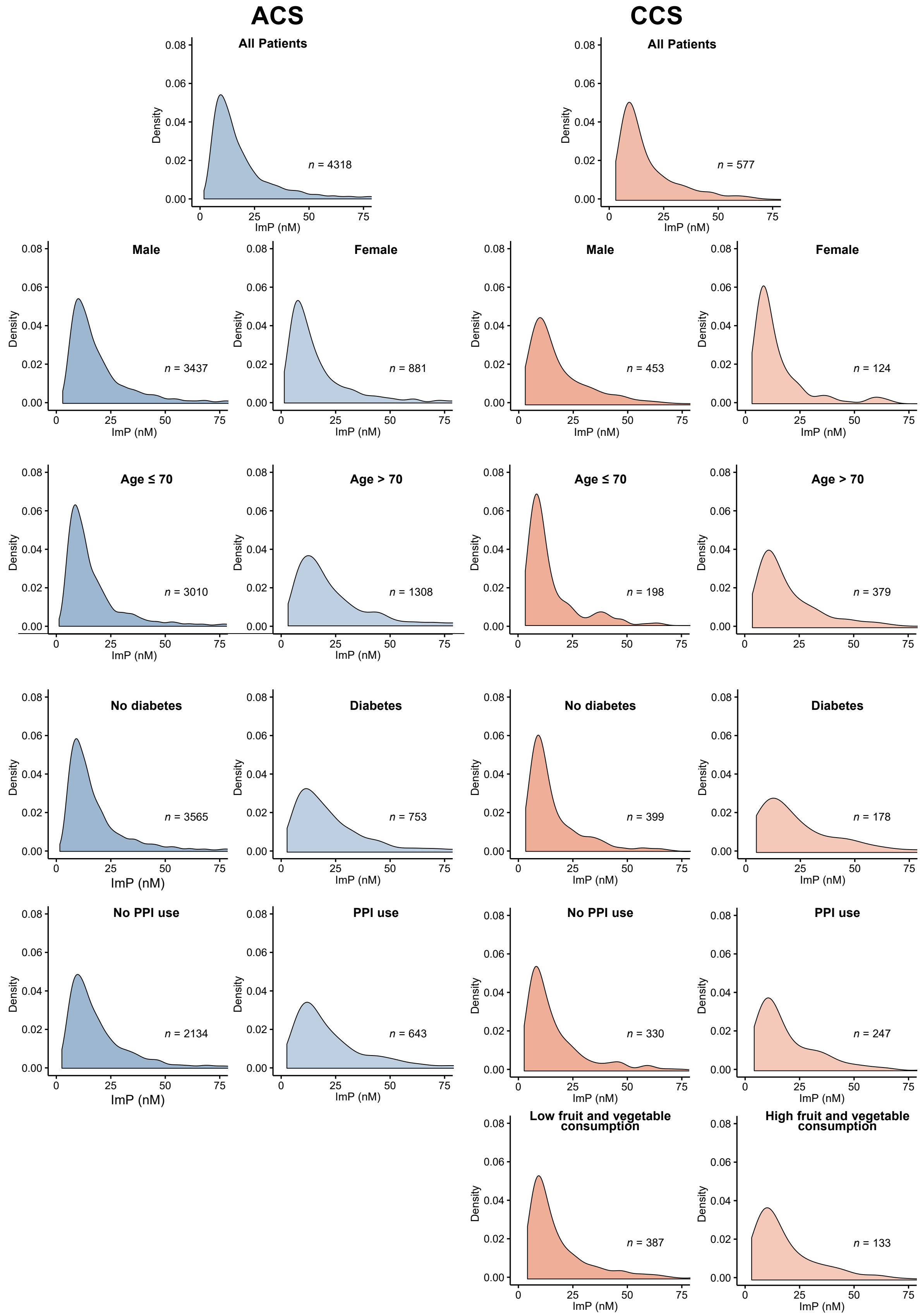

Population density plots of ImP levels in clinical subgroups of patients with ACS and CCS. ACS, acute coronary syndrome; CCS, chronic coronary syndrome; ImP, imidazole propionate; PPI, proton pump inhibitor.

Figure S6: Histidine and urocanate levels in patients with CAD (pooled data from Swiss ACS cohort and German CCS cohort)

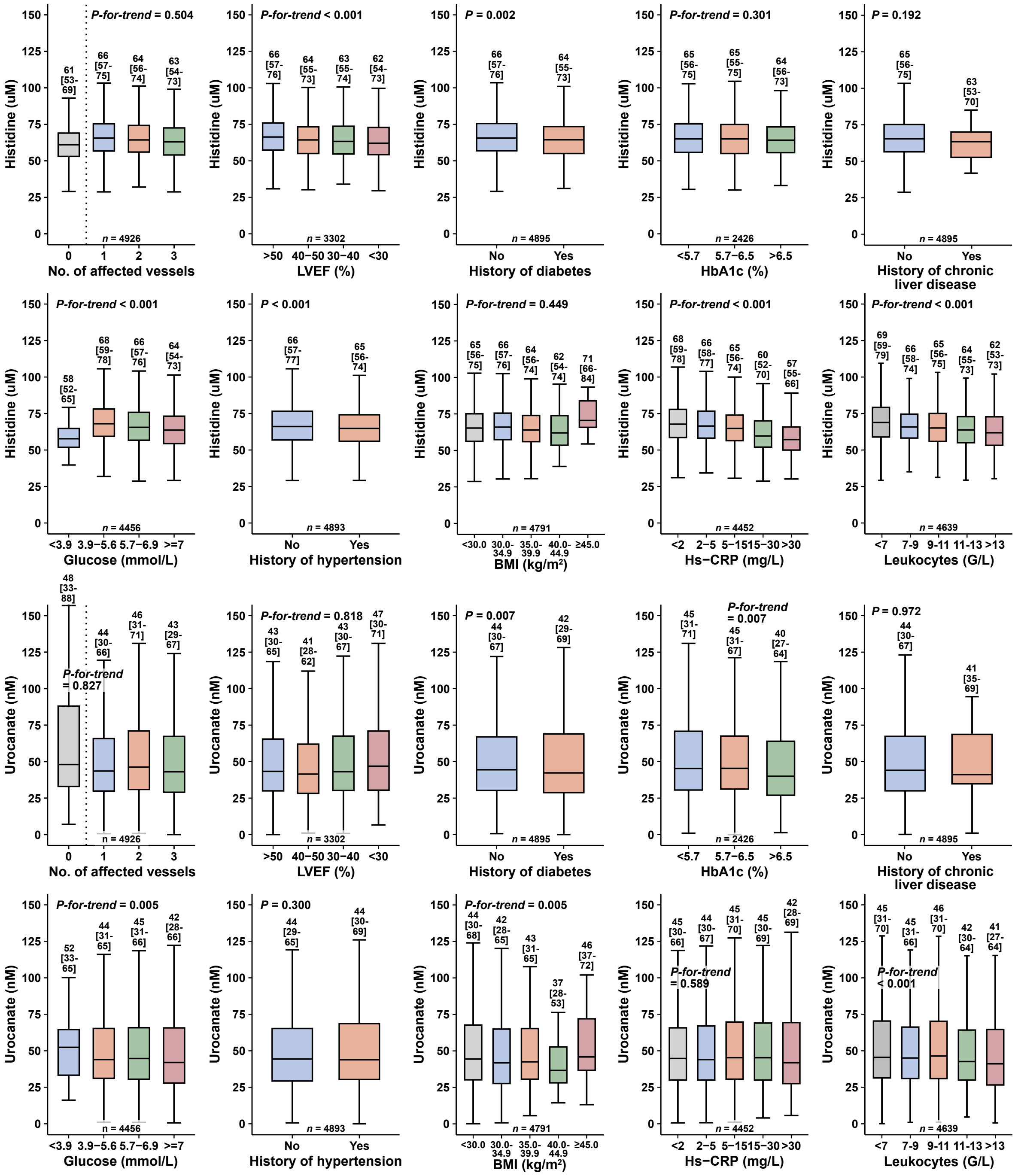

Values in the group with 0 affected vessels refer to the reference population without coronary artery disease. Medians with interquartile ranges are displayed on top of each box. Results are based on pooled data from the Swiss ACS cohort and the German CCS cohort. ACS, acute coronary syndrome; BMI, body mass index; CAD, coronary artery disease; CCS, chronic coronary syndrome; HbA1c, haemoglobin A1c; hs-CRP, high-sensitivity C-reactive protein; LVEF, left ventricular ejection fraction.

Figure S7: Imidazole propionate levels in patients with ACS (Swiss ACS cohort)

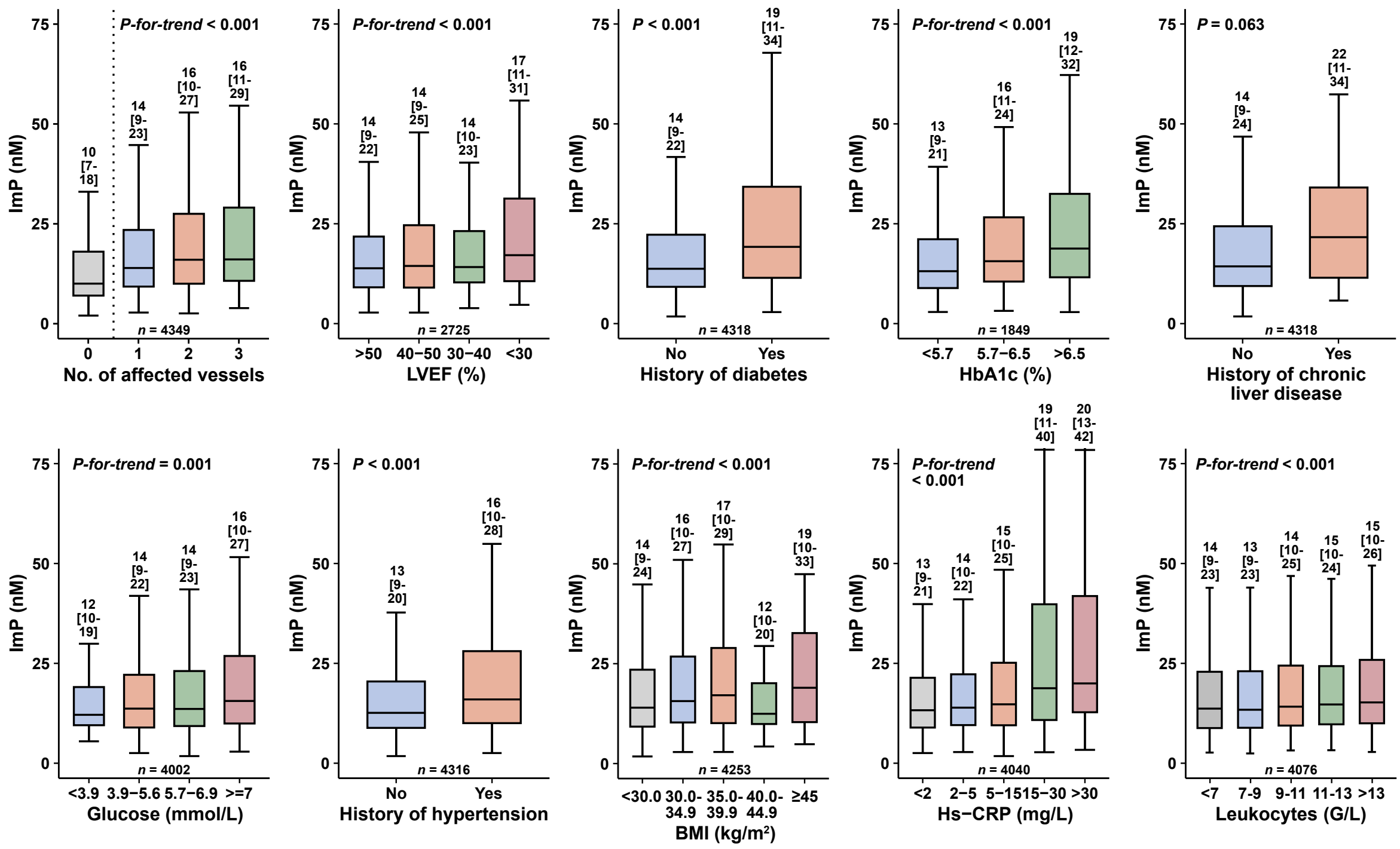

Values in the group with 0 affected vessels refer to the reference population without coronary artery disease. Medians with interquartile ranges are displayed on top of each box. ACS, acute coronary syndrome; BMI, body mass index; HbA1c, haemoglobin A1c; hs-CRP, high-sensitivity C-reactive protein; LVEF, left ventricular ejection fraction; ImP, imidazole propionate.

Figure S8: Histidine and urocanate levels in patients with ACS (Swiss ACS cohort)

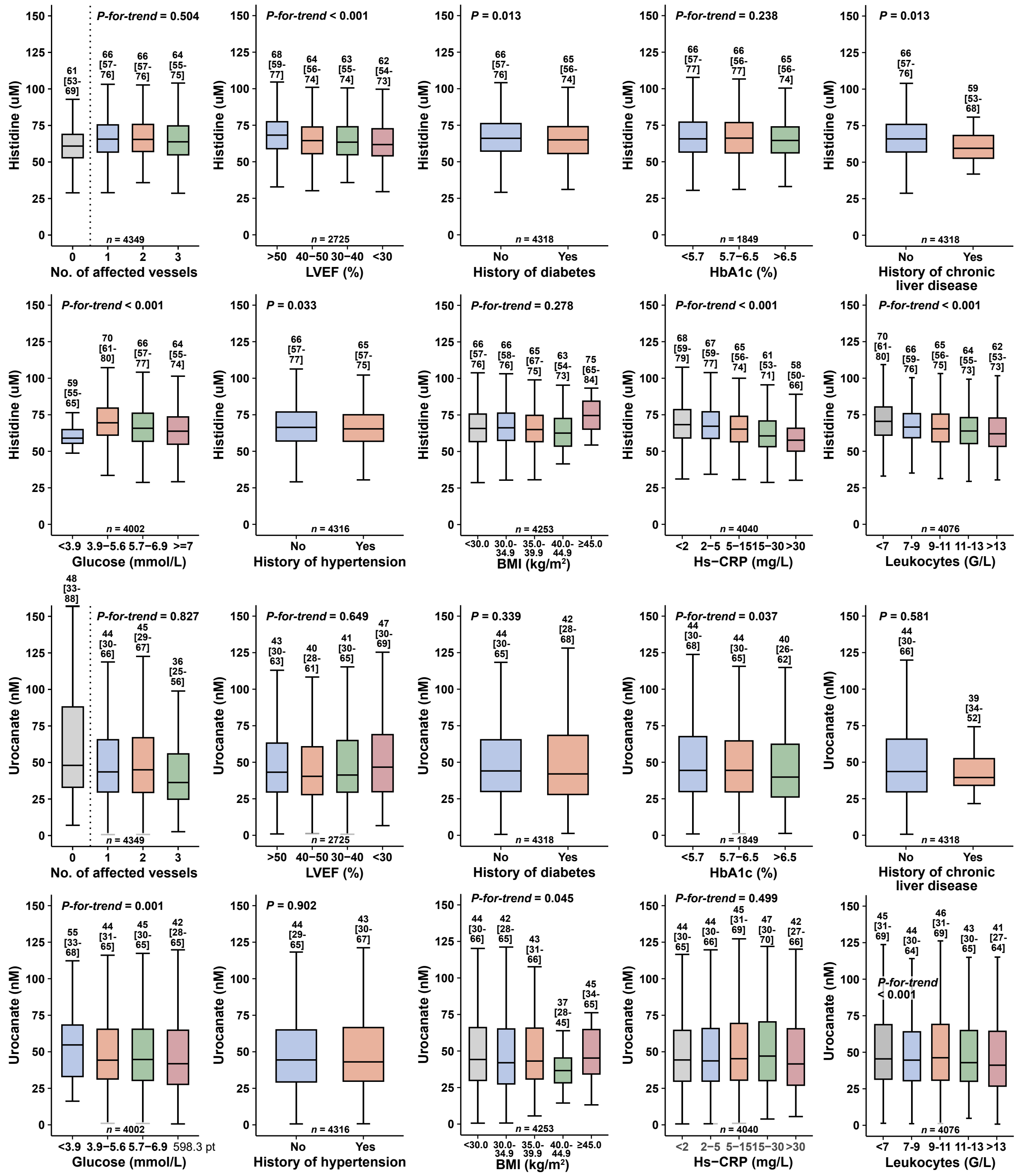

Values in the group with 0 affected vessels refer to the reference population without coronary artery disease. Medians with interquartile ranges are displayed on top of each box. ACS, acute coronary syndrome; BMI, body mass index; HbA1c, haemoglobin A1c; hs-CRP, high-sensitivity C-reactive protein; LVEF, left ventricular ejection fraction.

Figure S9: Imidazole propionate levels in patients with CCS (German CCS cohort)

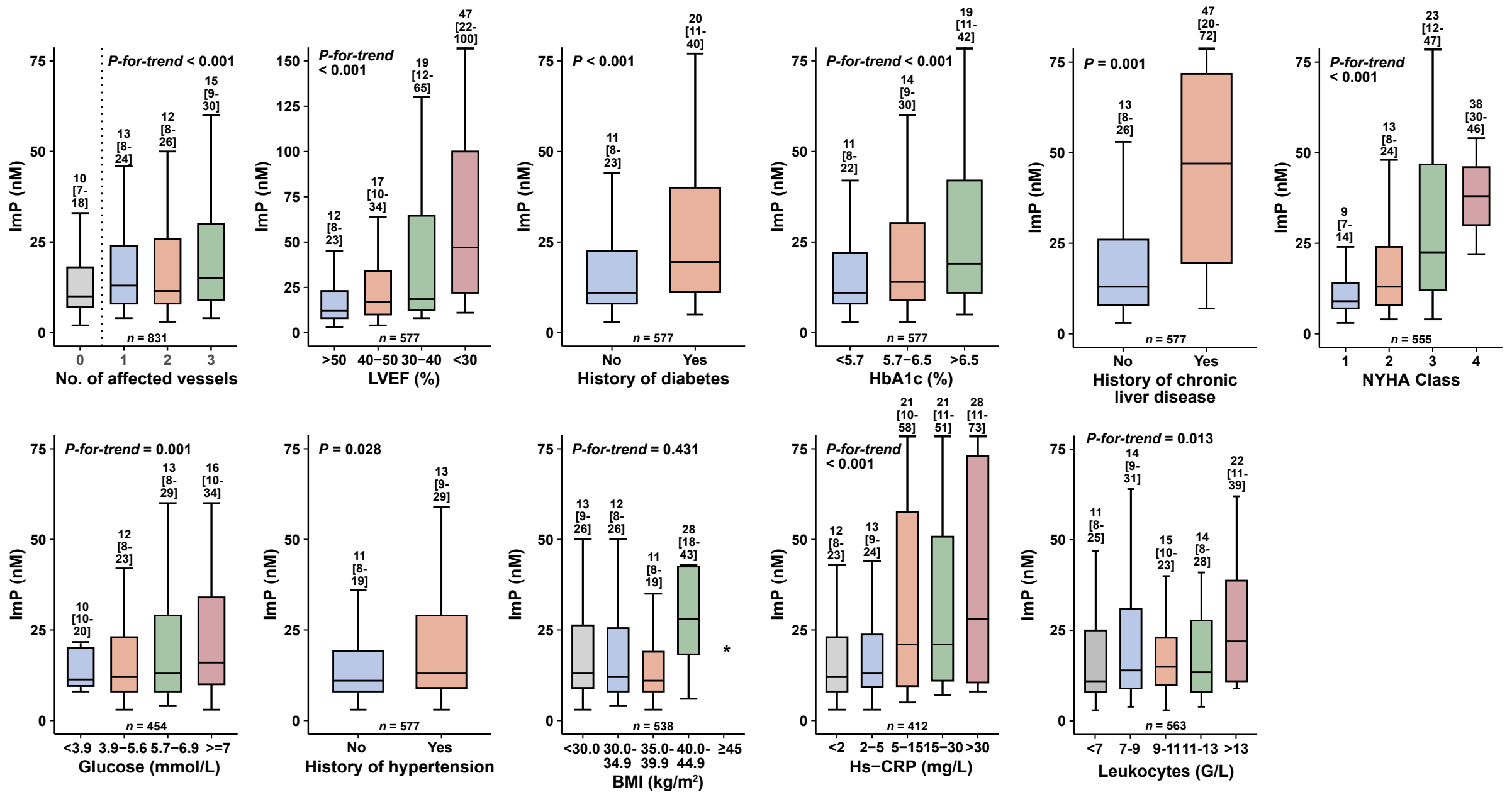

Values in the group with 0 affected vessels refer to the reference population without coronary artery disease. Medians with interquartile ranges are displayed on top of each box. BMI, body mass index; CCS, chronic coronary syndrome; HbA1c, haemoglobin A1c; hs-CRP, high-sensitivity C-reactive protein; LVEF, left ventricular ejection fraction; ImP, imidazole propionate; NYHA, New York Heart Association. *box plot omitted (n=1)

Figure S10: Histidine and urocanate levels in patients with CCS (German CCS cohort)

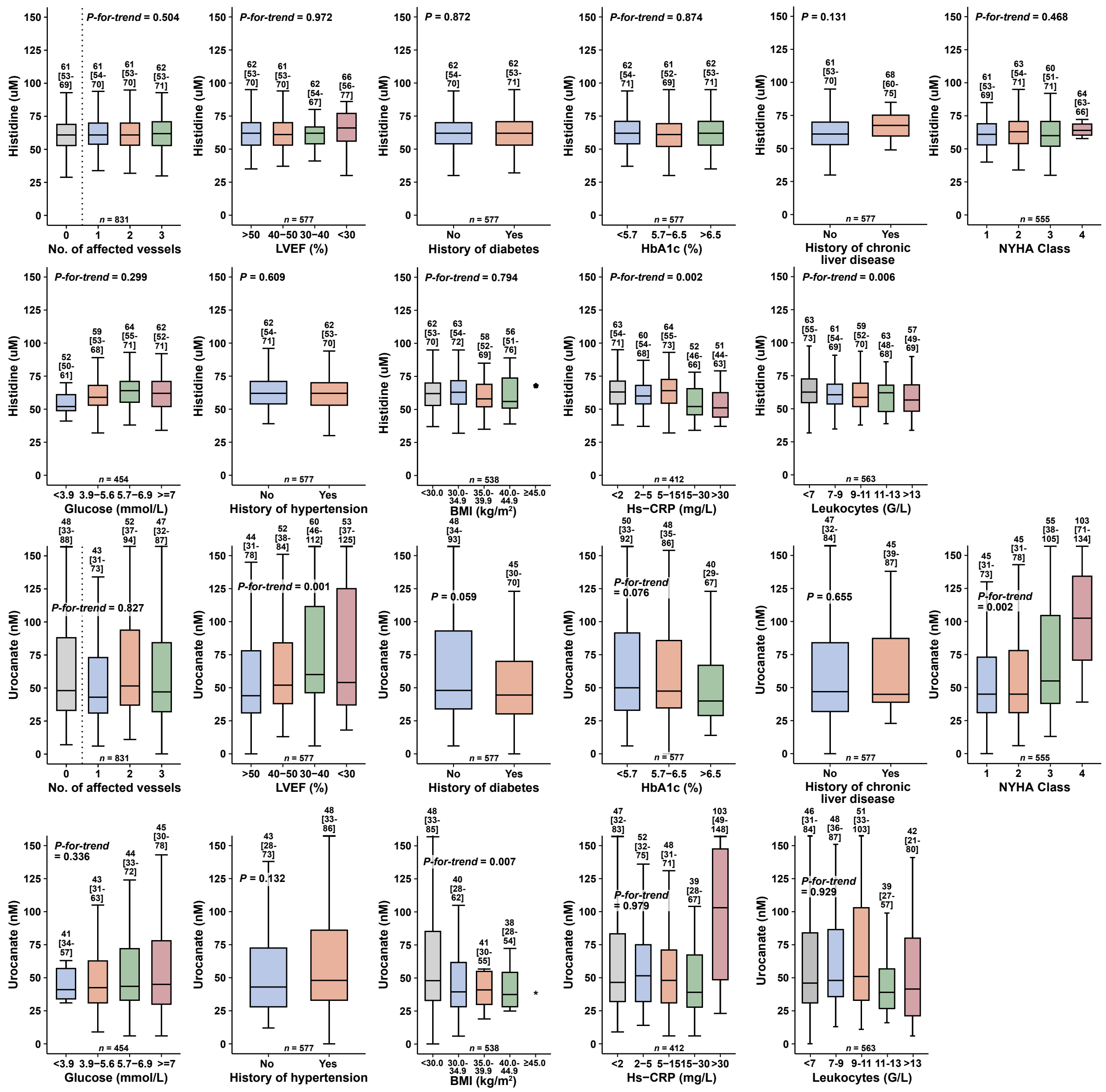

Values in the group with 0 affected vessels refer to the reference population without coronary artery disease. Medians with interquartile ranges are displayed on top of each box. BMI, body mass index; CCS, chronic coronary syndrome; HbA1c, haemoglobin A1c; hs-CRP, high-sensitivity C-reactive protein; LVEF, left ventricular ejection fraction; NYHA, New York Heart Association.*box plot omitted (n=1)

Figure S11: Association of imidazole propionate levels with differential blood count in patients with ACS (Swiss ACS cohort)

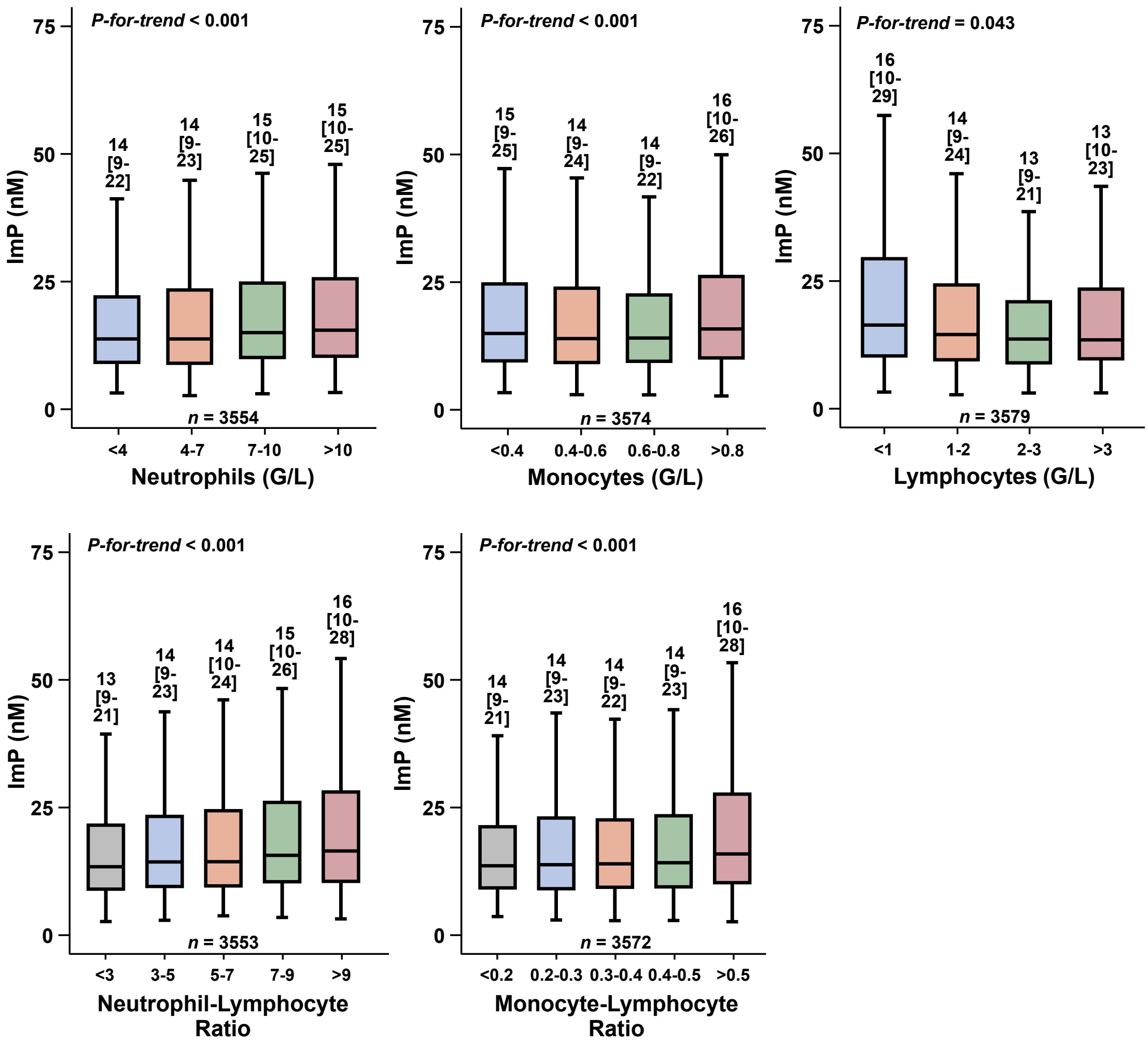

Medians with interquartile ranges are displayed on top of each box. ACS, acute coronary syndrome. ImP, imidazole propionate.

Figure S12: Association of histidine and urocanate levels and coronary microvascular obstruction in patients with ACS

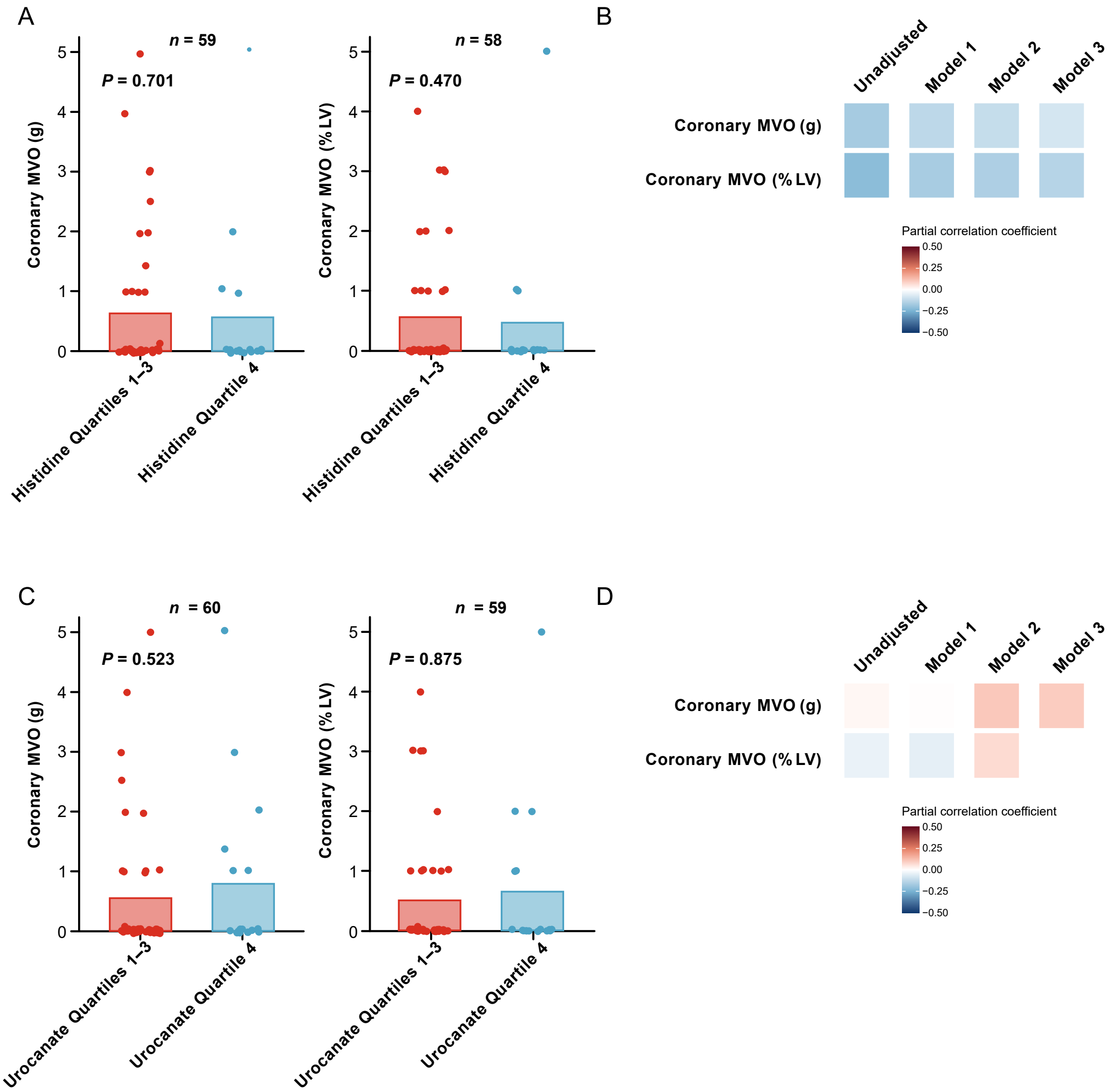

(A) Coronary MVO (g) and coronary MVO (% LV) at 30 days in patients with histidine quartile 4 versus quartiles 1–3. (B) Correlation matrix for histidine and coronary MVO (g), and coronary MVO (% LV). Spearman partial correlation coefficients and P-values were calculated using partial correlations adjusted for Model 1: age, sex, and diabetes status. Model 2: Model 1 plus hypertension, low-density lipoprotein cholesterol, and glucose, Model 3: Model 2 plus pre-procedural TIMI-flow. All false discovery rate (FDR)-adjusted *P-values* using Benjamini–Hochberg correction are ≥ 0.05. (C) Coronary MVO (g) and coronary MVO (% LV) at 30 days in patients with urocanate quartile 4 versus quartiles 1–3. (D) Correlation matrix for urocanate and coronary MVO (g), and coronary MVO (% LV). Bars are mean values. Spearman partial correlation coefficients and P-values were calculated using partial correlations adjusted for Model 1: age, sex, and diabetes status. Model 2: Model 1 plus hypertension, low-density lipoprotein cholesterol, and glucose, Model 3: Model 2 plus pre-procedural TIMI-flow. All FDR-adjusted *P-values* using Benjamini–Hochberg correction are ≥ 0.05. LV, left ventricular; MVO, microvascular obstruction; TIMI, thrombolysis in myocardial infarction. The cut-offs between 3rd and 4th quartile were 75.71 µmol/L for histidine and 65.68 nM for urocanate. Results are based on Swiss CMR cohort.

Figure S13: Combined association of imidazole propionate and TMAO with mortality in patients with ACS (Swiss ACS cohort) and in patients with CCS (German CCS cohort)

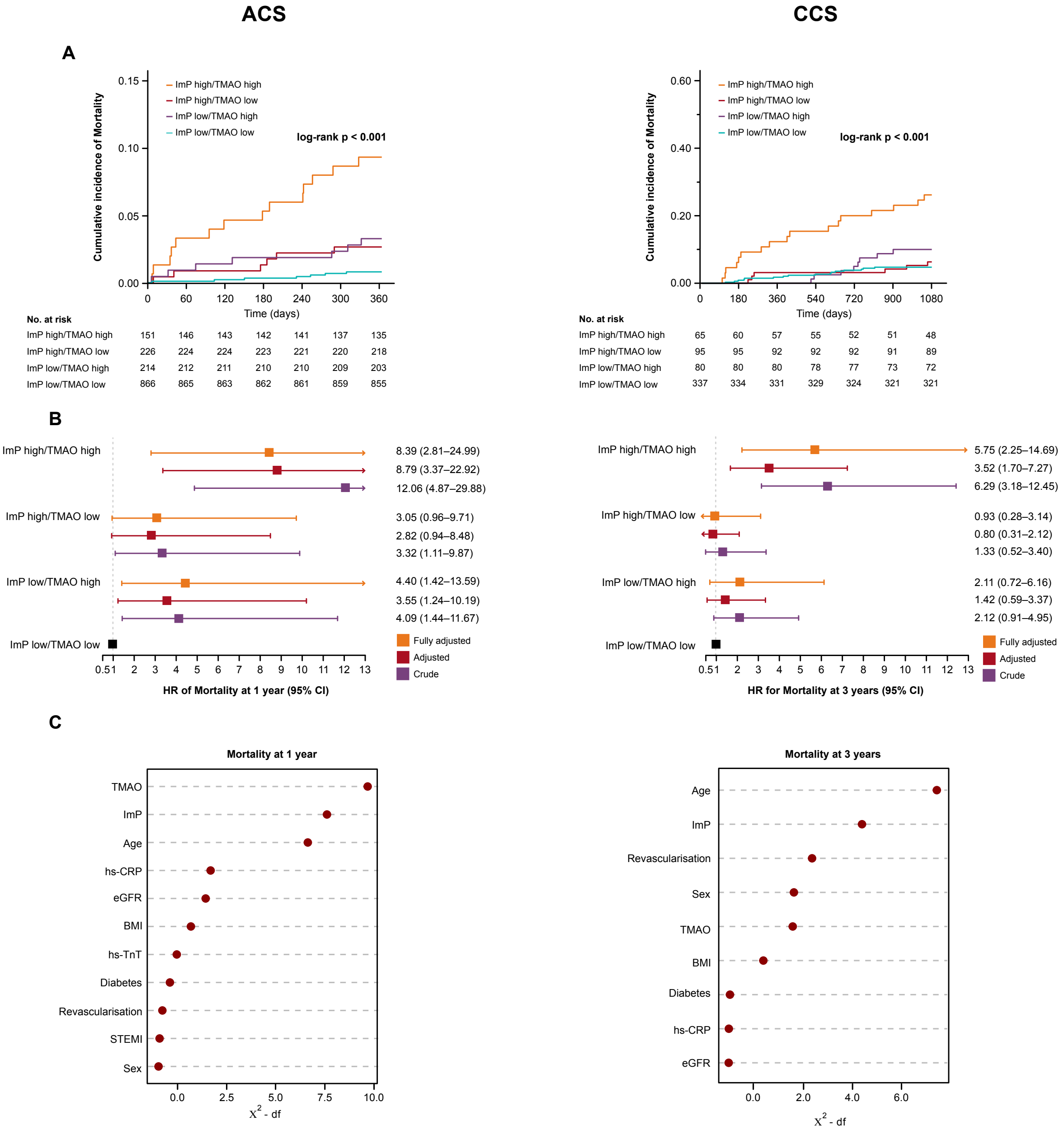

(A) Cumulative deaths at 1 year and at 3 years in patients presenting with acute coronary syndrome (ACS) and with chronic coronary syndrome (CCS), respectively, according to joint groups of high (≥ 75th percentile; ImP Q4: > 24.4 nM) *vs.* low (< 75th percentile; Q1-3: 0–24.4 nM) levels of imidazole propionate (ImP) and trimethylamine N-oxide (TMAO). (B) Crude and adjusted HR for 1-year and 3-year mortality in patients with ACS (n=1457) and CCS (n=577), respectively. Squares indicate point estimates of the HR and line lengths equal corresponding 95% CIs. (C) Variable importance in the fully adjusted Cox model for the prediction of mortality, measured by partial Wald χ^2 minus degrees of freedom. ACS, acute coronary syndrome; BMI, body mass index; CCS, chronic coronary syndrome; CI, confidence interval; eGFR, estimated glomerular filtration rate; HR, hazard ratio; hs-CRP, high-sensitivity C-reactive protein; hs-TnT, high-sensitivity troponin T; ImP, imidazole propionate; STEMI, ST-segment elevation myocardial infarction; TMAO, trimethylamine N-oxide.

Figure S14: Rehospitalisations according to imidazole propionate levels

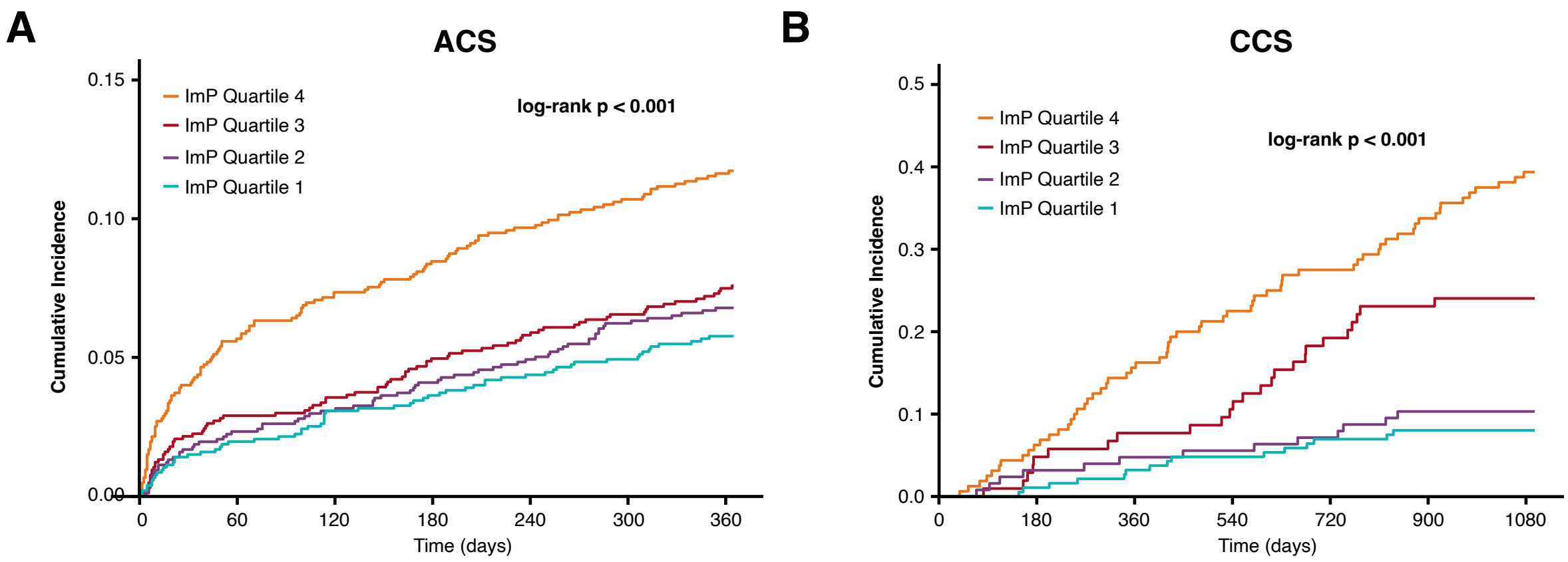

No. at risk (A)

	0	60	120	180	240	300	360
ImP Quartile 4	1080	1016	997	985	971	960	934
ImP Quartile 3	1076	1040	1032	1017	1008	1000	974
ImP Quartile 2	1082	1054	1046	1035	1025	1011	994
ImP Quartile 1	1080	1059	1047	1040	1032	1025	1003

No. at risk (B)

	0	180	360	540	720	900	1080
ImP Quartile 4	160	150	135	124	116	106	97
ImP Quartile 3	104	99	96	93	84	80	79
ImP Quartile 2	126	122	120	119	117	113	113
ImP Quartile 1	187	185	181	178	174	172	172

(A) Cumulative incidence of ischaemia-driven revascularisation or death according to imidazole propionate level in the Swiss ACS cohort. (B) Cumulative incidence of rehospitalisation for heart failure or death according to imidazole propionate level in the German CCS cohort. No data were available on ischaemia-driven revascularisation in CCS or on rehospitalisation for heart failure in ACS. To address competing risks, both non-fatal outcomes were analysed as composite endpoints incorporating death. ACS, acute coronary syndrome; CCS, chronic coronary syndrome; ImP, imidazole propionate.

Figure S15: STROBE Statement

	Item No	Recommendation	Page No
**Title and abstract**	1	(*a*) Indicate the study's design with a commonly used term in the title or the abstract	1, 2
		(*b*) Provide in the abstract an informative and balanced summary of what was done and what was found	2
**Introduction**			
Background/rationale	2	Explain the scientific background and rationale for the investigation being reported	4
Objectives	3	State specific objectives, including any prespecified hypotheses	4
**Methods**			
Study design	4	Present key elements of study design early in the paper	5
Setting	5	Describe the setting, locations, and relevant dates, including periods of recruitment, exposure, follow-up, and data collection	5-6, Suppl.
Participants	6	(*a*) Give the eligibility criteria, and the sources and methods of selection of participants. Describe methods of follow-up	5-8, Suppl.
		(*b*) For matched studies, give matching criteria and number of exposed and unexposed	5-7, Suppl.
Variables	7	Clearly define all outcomes, exposures, predictors, potential confounders, and effect modifiers. Give diagnostic criteria, if applicable	7-8, Suppl.
Data sources/ measurement	8*	For each variable of interest, give sources of data and details of methods of assessment (measurement). Describe comparability of assessment methods if there is more than one group	8-10, Suppl.
Bias	9	Describe any efforts to address potential sources of bias	8-10, Suppl.
Study size	10	Explain how the study size was arrived at	5-6, Suppl. figure 1
Quantitative variables	11	Explain how quantitative variables were handled in the analyses. If applicable, describe which groupings were chosen and why	8, Suppl.
Statistical methods	12	(*a*) Describe all statistical methods, including those used to control for confounding	8-10, Suppl.
		(*b*) Describe any methods used to examine subgroups and interactions	9, Suppl.
		(*c*) Explain how missing data were addressed	Suppl.
		(*d*) If applicable, explain how loss to follow-up was addressed	Suppl.
		(*e*) Describe any sensitivity analyses	Suppl.
**Results**			
Participants	13*	(a) Report numbers of individuals at each stage of study—eg numbers potentially eligible, examined for eligibility, confirmed eligible, included in the study, completing follow-up, and analysed	10, Suppl. figure 1
		(b) Give reasons for non-participation at each stage	Suppl.
		(c) Consider use of a flow diagram	Suppl. figure 1
Descriptive data	14*	(a) Give characteristics of study participants (eg demographic, clinical, social) and information on exposures and potential confounders	Table 1, Table 2, Suppl.
		(b) Indicate number of participants with missing data for each variable of interest	Suppl.
		(c) Summarise follow-up time (eg, average and total amount)	7-8, Suppl.
Outcome data	15*	Report numbers of outcome events or summary measures over time	Table 2, Suppl.

Main results	16	(*a*) Give unadjusted estimates and, if applicable, confounder-adjusted estimates and their precision (eg, 95% confidence interval). Make clear which confounders were adjusted for and why they were included<br>(*b*) Report category boundaries when continuous variables were categorised<br>(*c*) If relevant, consider translating estimates of relative risk into absolute risk for a meaningful time period	10-12, Suppl.<br><br>Suppl. Figure 4, Suppl.
Other analyses	17	Report other analyses done—eg analyses of subgroups and interactions, and sensitivity analyses	10-12, Suppl.
**Discussion**			
Key results	18	Summarise key results with reference to study objectives	12-13
Limitations	19	Discuss limitations of the study, taking into account sources of potential bias or imprecision. Discuss both direction and magnitude of any potential bias	15-16
Interpretation	20	Give a cautious overall interpretation of results considering objectives, limitations, multiplicity of analyses, results from similar studies, and other relevant evidence	13-14
Generalisability	21	Discuss the generalisability (external validity) of the study results	16
**Other information**			
Funding	22	Give the source of funding and the role of the funders for the present study and, if applicable, for the original study on which the present article is based	20-21

References

1. Wenzl FA, Bruno F, Kraler S, Klingenberg R, Akhmedov A, Ministrini S*, et al.* Dipeptidyl peptidase 3 plasma levels predict cardiogenic shock and mortality in acute coronary syndromes. *Eur Heart J* 2023;**44**:3859-3871. doi: 10.1093/eurheartj/ehad545
2. Kraler S, Wenzl FA, Georgiopoulos G, Obeid S, Liberale L, von Eckardstein A*, et al.* Soluble lectin-like oxidized low-density lipoprotein receptor-1 predicts premature death in acute coronary syndromes. *Eur Heart J* 2022;**43**:1849-1860. doi: 10.1093/eurheartj/ehac143
3. Stähli BE, Klingenberg R, Heg D, Branca M, Manka R, Kapos I*, et al.* Mammalian Target of Rapamycin Inhibition in Patients With ST-Segment Elevation Myocardial Infarction. *J Am Coll Cardiol* 2022;**80**:1802-1814. doi: 10.1016/j.jacc.2022.08.747
4. Klingenberg R, Stähli BE, Heg D, Denegri A, Manka R, Kapos I*, et al.* Controlled-Level EVERolimus in Acute Coronary Syndrome (CLEVER-ACS) - A phase II, randomized, double-blind, multi-center, placebo-controlled trial. *Am Heart J* 2022;**247**:33-41. doi: 10.1016/j.ahj.2022.01.010
5. Stamatelopoulos K, Mueller-Hennessen M, Georgiopoulos G, Lopez-Ayala P, Sachse M, Vlachogiannis NI*, et al.* Cathepsin S Levels and Survival Among Patients With Non-ST-Segment Elevation Acute Coronary Syndromes. *J Am Coll Cardiol* 2022;**80**:998-1010. doi: 10.1016/j.jacc.2022.05.055
6. Thygesen K, Alpert JS, Jaffe AS, Chaitman BR, Bax JJ, Morrow DA*, et al.* Fourth universal definition of myocardial infarction (2018). *European Heart Journal* 2019;**40**:237-269. doi: 10.1093/eurheartj/ehy462
7. König M, Joshi S, Leistner DM, Landmesser U, Sinning D, Steinhagen-Thiessen E*, et al.* Cohort profile: role of lipoproteins in cardiovascular disease-the LipidCardio study. *BMJ Open* 2019;**9**:e030097. doi: 10.1136/bmjopen-2019-030097
8. Li XS, Obeid S, Klingenberg R, Gencer B, Mach F, Raber L*, et al.* Gut microbiota-dependent trimethylamine N-oxide in acute coronary syndromes: a prognostic marker for incident cardiovascular events beyond traditional risk factors. *Eur Heart J* 2017;**38**:814-824. doi: 10.1093/eurheartj/ehw582
9. Witkowski M, Witkowski M, Friebel J, Buffa JA, Li XS, Wang Z*, et al.* Vascular endothelial tissue factor contributes to trimethylamine N-oxide-enhanced arterial thrombosis. *Cardiovasc Res* 2022;**118**:2367-2384. doi: 10.1093/cvr/cvab263
10. Narula S, Yusuf S, Chong M, Ramasundarahettige C, Rangarajan S, Bangdiwala SI*, et al.* Plasma ACE2 and risk of death or cardiometabolic diseases: a case-cohort analysis. *Lancet* 2020;**396**:968-976. doi: 10.1016/s0140-6736(20)31964-4
11. O'Brien KM, Lawrence KG, Keil AP. The Case for Case-Cohort: An Applied Epidemiologist's Guide to Reframing Case-Cohort Studies to Improve Usability and Flexibility. *Epidemiology* 2022;**33**:354-361. doi: 10.1097/ede.0000000000001469
12. Qiu X, Yang J, Hu X, Li J, Zhao M, Ren F*, et al.* Association between hearing ability and cortical morphology in the elderly: multiparametric mapping, cognitive relevance, and neurobiological underpinnings. *EBioMedicine* 2024;**104**:105160. doi: 10.1016/j.ebiom.2024.105160
13. Du Bois D, Du Bois EF. A formula to estimate the approximate surface area if height and weight be known. 1916. *Nutrition* 1989;**5**:303-311; discussion 312-303. doi:
14. Wenzl FA, Wang P, Arrigo M, Parenica J, Jones DJL, Bruno F*, et al.* Proenkephalin Improves Cardio-Renal Risk Prediction in Acute Coronary Syndromes: The KID-ACS Score. *Eur Heart J* 2024. doi: 10.1093/eurheartj/ehae602
15. Grund B, Sabin C. Analysis of biomarker data: logs, odds ratios, and receiver operating characteristic curves. *Curr Opin HIV AIDS* 2010;**5**:473-479. doi: 10.1097/COH.0b013e32833ed742
16. Molinaro A, Bel Lassen P, Henricsson M, Wu H, Adriouch S, Belda E*, et al.* Imidazole propionate is increased in diabetes and associated with dietary patterns and altered microbial ecology. *Nat Commun* 2020;**11**:5881. doi: 10.1038/s41467-020-19589-w
17. Fox KA, Fitzgerald G, Puymirat E, Huang W, Carruthers K, Simon T*, et al.* Should patients with acute coronary disease be stratified for management according to their risk? Derivation, external validation and outcomes using the updated GRACE risk score. *BMJ Open* 2014;**4**:e004425. doi: 10.1136/bmjopen-2013-004425
18. Molinaro A, Nemet I, Bel Lassen P, Chakaroun R, Nielsen T, Aron-Wisnewsky J*, et al.* Microbially Produced Imidazole Propionate Is Associated With Heart Failure and Mortality. *JACC Heart Fail* 2023;**11**:810-821. doi: 10.1016/j.jchf.2023.03.008
